# Gradual evolution of allopolyploidy in *Arabidopsis suecica*

**DOI:** 10.1038/s41559-021-01525-w

**Published:** 2021-08-19

**Authors:** Robin Burns, Terezie Mandáková, Joanna Gunis, Luz Mayela Soto-Jiménez, Chang Liu, Martin A. Lysak, Polina Yu. Novikova, Magnus Nordborg

**Affiliations:** 1 Gregor Mendel Institute, Austrian Academy of Sciences, Vienna BioCenter, Vienna, Austria; 2 CEITEC - Central European Institute of Technology, and Faculty of Science, Masaryk University, Brno, Czech Republic; 3 Institute of Biology, University of Hohenheim, Stuttgart, Germany; 4 VIB-UGent Center for Plant Systems Biology, Ghent, Belgium; 5 Department of Chromosome Biology, Max Planck Institute for Plant Breeding Research, Cologne, Germany

**Keywords:** Speciation, Evolutionary genetics

## Abstract

Most diploid organisms have polyploid ancestors. The evolutionary process of polyploidization is poorly understood but has frequently been conjectured to involve some form of ‘genome shock’, such as genome reorganization and subgenome expression dominance. Here we study polyploidization in *Arabidopsis suecica*, a post-glacial allopolyploid species formed via hybridization of *Arabidopsis thaliana* and *Arabidopsis arenosa*. We generated a chromosome-level genome assembly of *A. suecica* and complemented it with polymorphism and transcriptome data from all species. Despite a divergence around 6 million years ago (Ma) between the ancestral species and differences in their genome composition, we see no evidence of a genome shock: the *A. suecica* genome is colinear with the ancestral genomes; there is no subgenome dominance in expression; and transposon dynamics appear stable. However, we find changes suggesting gradual adaptation to polyploidy. In particular, the *A. thaliana* subgenome shows upregulation of meiosis-related genes, possibly to prevent aneuploidy and undesirable homeologous exchanges that are observed in synthetic *A. suecica*, and the *A. arenosa* subgenome shows upregulation of cyto-nuclear processes, possibly in response to the new cytoplasmic environment of *A. suecica*, with plastids maternally inherited from *A. thaliana*. These changes are not seen in synthetic hybrids, and thus are likely to represent subsequent evolution.

## Abstract (web summary)

*Arabidopsis suecica* is a natural allotetraploid species formed via hybridization of *Arabidopsis thaliana* and *Arabidopsis arenosa*. Comparative analysis of genome and transcriptome data shows no evidence for major genomic changes linked to structural and functional alterations in *A. suecica* but reveals changes to the meiotic machinery and cyto-nuclear processes.

## Main

Ancient polyploidization or whole-genome duplication is a hallmark of most higher-organism genomes^1,2^, including our own^3,4^. While most of these organisms are now diploid and show only traces of polyploidy, there are many examples of recent polyploidization, especially among flowering plants^5–9^. These examples are important because they allow us to study the process of polyploidization.

Widespread naturally occurring polyploid hybrids (that is, allopolyploids) show that natural polyploid species can quickly become successful^10–18^ and even invasive^19^. However, new allopolyploid species face numerous challenges such as population bottlenecks^13,20^, competition with their diploid progenitors^21^, chromosome segregation^22–24^, changes to genome structure^25^ and genome regulation^26,27^—potentially leading to a ‘genome shock’^28^. In agreement with this, genomic and transcriptomic changes tied to the hybridization of diverged genomes have been reported in resynthesized polyploids of wheat^29–35^, *Brassica napus*^36–38^ and cotton^39–42^, although exceptions exist^43^.

The long-term importance of such changes is unclear. Evidence of the transcription and mobilization of transposable elements (TEs) in resynthesized wheat^33,44–46^ is not observed in cultivated wheat^47^. However, other cultivated crop genomes, like cotton, show evidence consistent with dramatic changes following allopolyploidy^5,48–53^. Strawberry^6^, peanut^8^ and the mesopolyploids *Brassica rapa*^54^ and maize^55^ show evidence of subgenome dominance, whereas wheat^56^, cotton^51^ and *B. napus*^57^ do not. The reasons for these differences are not understood.

Whether allopolyploid crops are representative of natural polyploidization is unclear. Domestication is frequently associated with very strong ‘artificial’ selection, which can markedly alter the fitness landscape^58–62^, and structural variants have been linked to favourable agronomic traits^63–65^. In addition, polyploid crops are generally evolutionarily recent.

Genomic changes have also been reported in natural allopolyploids such as *Tragopogon miscellus*^66,67^, *Mimulus pergrinus*^17^ and *Spartina anglica*^68^; however, these examples resemble resynthesized allopolyploids in being extremely recent (around 100 years). More-established allopolyploids generally do not show signs of genomic changes^12–14,16,69–71^.

**Fig. 1:**
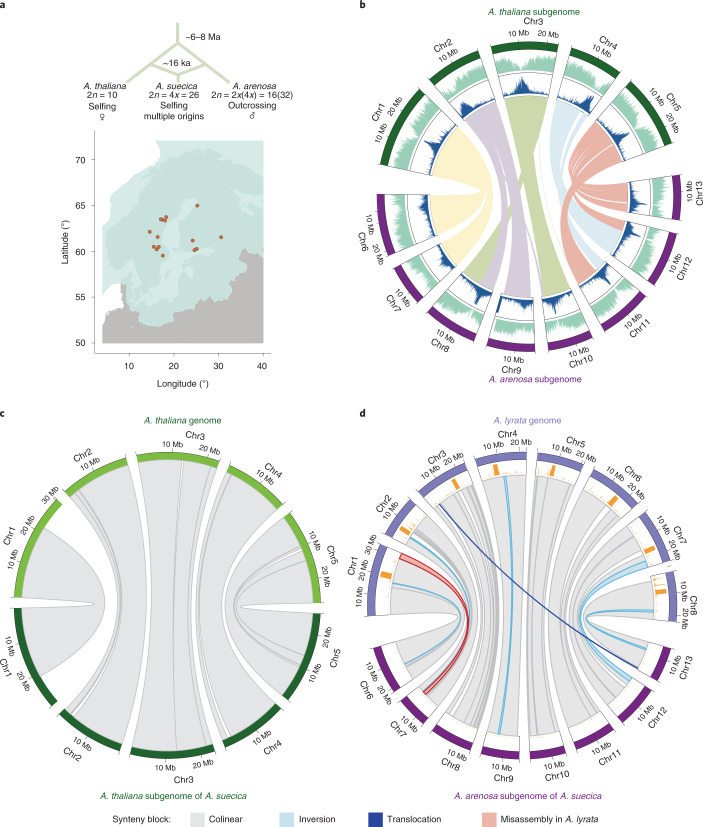
The genome of *A. suecica* is largely colinear with the ancestral genomes. **a**, Schematic depicting the origin of *A. suecica* and its current distribution in relation to the ice cover at the Last Glacial Maximum. ka, thousand years ago. Ice cover data are from Natural Resource Canada (https://open.canada.ca/data/en/dataset/a384bada-a787-5b49-9799-f5d589e97bd3). **b**, Chromosome-level assembly of the *A. suecica* genome with inner links depicting syntenic blocks between the *A. thaliana* and *A. arenosa* subgenomes of *A. suecica*. Histograms show the distribution of TEs (in blue) and protein-coding genes (in green) along the chromosomes. **c**, Synteny of the *A. thaliana* subgenome of *A. suecica* to the *A. thaliana* TAIR10 reference. In total 13 colinear synteny blocks were found. **d**, Synteny of the *A. arenosa* subgenome to *A. lyrata*. In total 40 synteny blocks were found, 33 of which were colinear. Of the remaining seven blocks, five represent inversions in the *A. arenosa* subgenome of *A. suecica* relative to *A. lyrata*, one is a translocation and one corresponds to a previously reported misassembly in the *A. lyrata* genome^77^. Orange bars show the density of missing regions (‘N’ bases) in the *A. lyrata* genome.

Here, we focus on an allopolyploid comparable in age to these examples: the highly selfing^72^ *A. suecica*, which was formed through the hybridization of *A. thaliana* and *A. arenosa* around 16 thousand years ago, during the Last Glacial Maximum^20^, and which is currently found in northern Fennoscandia (Fig. 1a). The ancestral species diverged around 6 Ma (ref. ^73^), and based on organelle sequences, *A. thaliana* is the maternal and *A. arenosa* is the paternal parent^74^. This scenario is supported by *A. arenosa* being a ploidy-variable species, such that *A. suecica* could readily be generated by the fusion of an unreduced *A. thaliana* egg cell and an autotetraploid *A. arenosa* sperm cell^20,75^. Despite a severe genetic bottleneck^20^, most of the genetic variation in *A. suecica* is shared with the ancestral species, ruling out a unique origin. To study genomic change in *A. suecica*, we used long-read sequencing to generate a chromosome-level genome sequence, complemented by a partial assembly of a tetraploid *A. arenosa*, and by short-read genome and transcriptome sequencing data from populations of all three species—including ‘synthetic’ *A. suecica*. Our main goal was to look for evidence of genome changes (particularly the kind of dramatic changes discussed above) in *A. suecica* relative to the ancestral species.

## Results and analysis

### The genome is conserved

**Extended Data Fig. 1:**
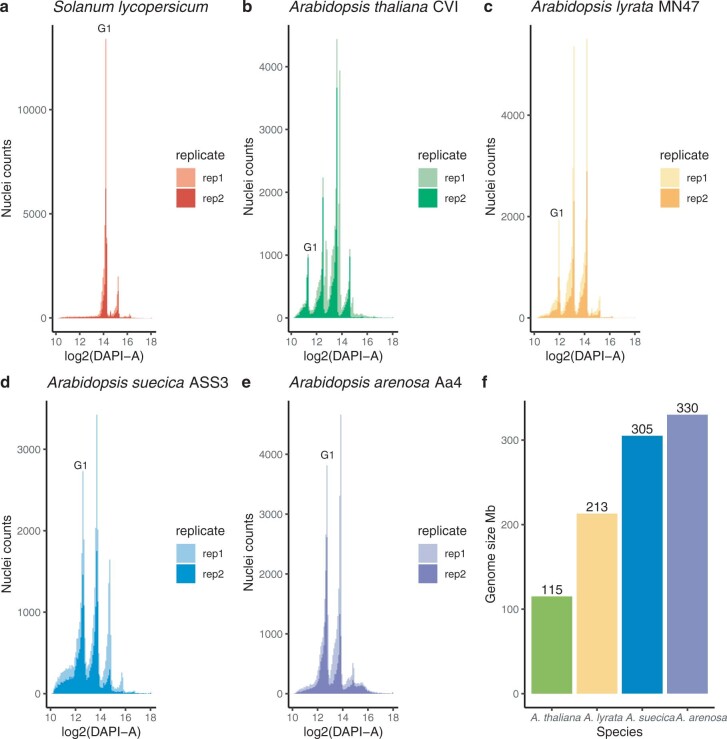
Measuring genome sizes of *Arabidopsis* species using flow cytometry. **a**, FACS sorting of *Solanum lycopersicum* cells from 3-week-old leaf tissue for two replicates. G1 represents the peak denoting the G1 phase of the cell cycle. Cells in the G1 phase have 2 C DNA content (that is a 2 N genome). **b**, *A. thaliana* ‘CVI’ accession **c**, *A. lyrata* ‘MN47’ (the reference accession) **d**, *A. suecica* ‘ASS3’ (the reference accession) **e** autopolyploid *A. arenosa* accession ‘Aa4’ **f**, Bar chart shows calculated genome sizes (rounded to the nearest whole number) for each species using *Solanum lycopersicum* as the standard.

**Extended Data Fig. 2:**
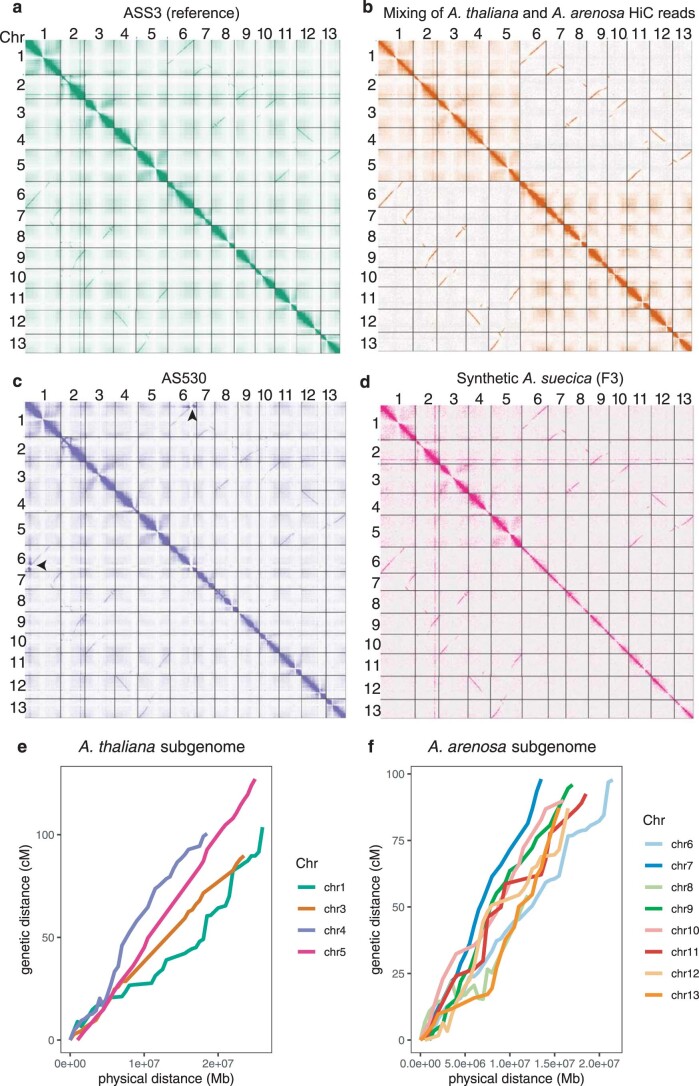
Hi-C and a genetic map analysis for the A. suecica genome. **a**, Hi-C contact map for the genome of *A. suecica*. **b**, Mixing of *A. thaliana* and *A. arenosa* Hi-C reads suggest interchromosomal contacts between homeologous chromosomes is a result of mis-mapping for Hi-C reads. **c**, Accession ‘AS530’ with the region of HE highlighted with an arrow (Fig. 6), no other rearrangements were observed. **d**, Hi-C of synthetic *A. suecica* (third selfed generation). **e** and **f** physical distance (Mb) vs genetic distance (cM) is plotted for the *A. thaliana* and *A. arenosa* subgenome, respectively. Chromosome 2 is not plotted as there are too few SNPs on this chromosome in our cross, due to the recent bottleneck in *A. suecica*.

We assembled a reference genome from a naturally inbred^20,72^ *A. suecica* accession (ASS3), using 50× long-read PacBio sequencing (PacBio RSII). The absence of heterozygosity and the substantial (around 11.6%) divergence between the subgenomes facilitated the assembly. By contrast, assembling *A. arenosa* is complicated by high heterozygosity (around 3.5% nucleotide diversity^76^) and high repeat content. Our assembly of a tetraploid *A. arenosa* individual, included in this study, is fragmented: 3,629 contigs; N50 of 331 kb. The *A. suecica* assembly, however, has an N50 contig size of 9.02 Mb. Contigs totalled 276 Mb (around 90% and around 88% of genome size estimated by flow cytometry and *k*-mer analysis, respectively; see Extended Data Fig. 1 and Methods). Contigs were placed into scaffolds using chromatin conformation capture (Hi-C) data and using the reference genomes of *A. thaliana* and *Arabidopsis lyrata* (as substitute for *A. arenosa*) as guides. This resulted in 13 chromosome-scale scaffolds (Extended Data Fig. 2). The placement and orientation of each contig within a scaffold was confirmed and corrected using a genetic map for *A. suecica* (see Methods and Extended Data Fig. 2). The final assembly (Fig. 1b) contains 262 Mb and has an N50 scaffold size of 19.59 Mb. The 5 + 8 chromosomes of the *A. thaliana* and *A. arenosa* subgenomes sum to 119 Mb and 143 Mb, respectively.

Of the *A. thaliana* and *A. arenosa* subgenomes of *A. suecica*, 108 Mb and 135 Mb are in large blocks syntenic to the genomes of the ancestral species: 13 and 40 blocks, respectively (Fig. 1c,d). The majority of these syntenic blocks are colinear, with the exception of five small-scale inversions (around 4.5 Mb) and one translocation (around 244 kb) on the *A. arenosa* subgenome—which may reflect differences between *A. lyrata* and *A. arenosa*, two highly polymorphic species separated by about a million years^73,76^. We also corrected the described^77^ misassembly in the *A. lyrata* reference genome using our genetic map. Overall we find that approximately 93% of the *A. suecica* genome is syntenic to the ancestral genomes (Fig. 1c,d). This highlights the conservation of the *A. suecica* genome and contrasts with the major rearrangements that have been observed in several resynthesized polyploids^29,32,34,36^ and some crops^48,50,78^. Notably, major rearrangements have also been observed in synthetic *A. suecica*^79^.

**Extended Data Fig. 3:**
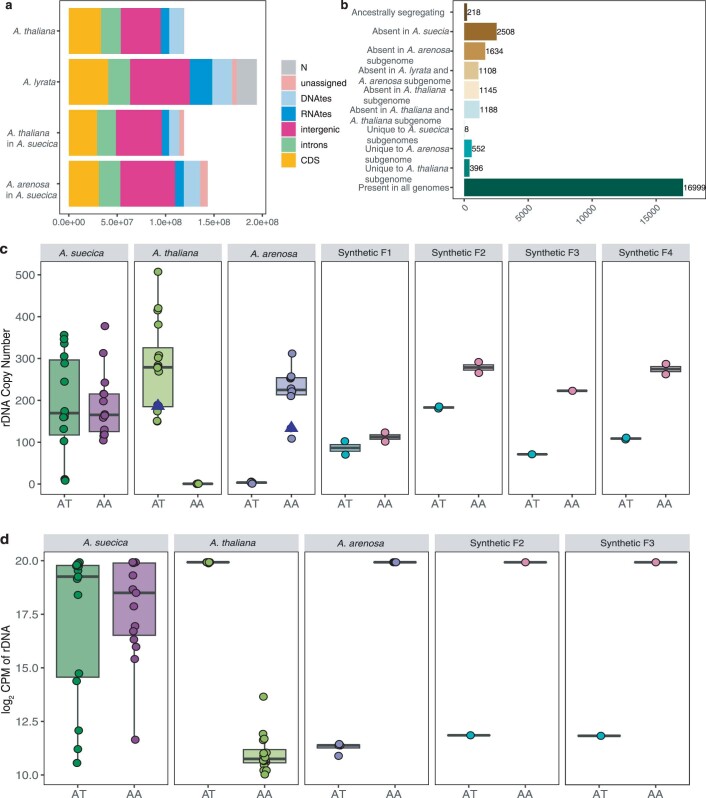
Genome composition and analysis of orthologues and the rDNA. **a**, Genome composition of the *A. suecica* subgenomes and the ancestral genomes of *A. thaliana* and *A. lyrata* (here a substitute reference for *A. arenosa* because it is annotated). **b**, Counts of orthologous genes between the subgenomes of the reference *A. suecica* genome and the reference *A. thaliana* and *A. lyrata* genome. **c**, Copy number of *A. thaliana* and *A. arenosa* rDNA in natural *A. suecica*, ancestral species and synthetic lines. Blue triangles represent the *A. thaliana* and *A. arenosa* parent lines of the synthetic *A. suecica* cross. AT represents results when mapping to the *A. thaliana* consensus sequence and AA to the *A. arenosa* consensus sequences for the 45S rRNA **b**, Expression (log_2_(CPM)) of *A. thaliana* and *A. arenosa* rDNA in natural *A. suecica*, ancestral species and synthetic lines. Accessions with log_2_(CPM) of > =15 was taken as evidence for expression for the *A. thaliana* and *A. arenosa* 45S rRNA in *A. suecica*, as this CPM value was above the maximum level of mis-mapping observed in the ancestral species (*A. thaliana* mapping to the *A. arenosa* 45S rRNA).

A total of 45,585 protein-coding genes were annotated for the *A. suecica* reference, of which 22,232 and 23,353 are located on the *A. thaliana* and *A. arenosa* subgenomes, respectively. We assessed completeness of the genome assembly and annotation with the BUSCO set for eudicots and found 2,088 (98.4%) complete genes for both the *A. thaliana* and *A. arenosa* subgenomes. Of the protein-coding genes, 18,023 had a one-to-one orthology between the subgenomes of *A. suecica* and 16,999 genes were conserved single-copy orthologues for each (sub-)genome of *A. suecica* and the ancestral species (Supplementary Data 2 and Extended Data Fig. 3). We annotated lineage-specific genes in *A. suecica* (that is, genes in *A. suecica* with no orthologue) using InterPro. We found significant enrichment in the *A. thaliana* subgenome for two gene ontology (GO) terms (GO:0008234 and GO:0015074) that are associated with repeat content (Supplementary Data 2). Ancestral genes missing in our annotation were overrepresented for functional categories of defence response. Examining DNA-sequencing coverage for these genes in the ancestral genomes did not confirm any gene loss, suggesting rather misassembly or misannotation, likely because of the repetitive and highly polymorphic nature of resistance genes (R-genes).

### The ribosomal DNA clusters are highly variable

In eukaryotic genomes, genes encoding ribosomal RNA (rRNA) occur as tandem arrays in rDNA clusters. The 45S rDNA clusters are massive, containing hundreds or thousands of copies and spanning millions of base pairs^80^. The site of pre-ribosome assembly (nucleolus), forms at these clusters if they are actively transcribed. In inter-specific hybrids it was previously observed that the rDNA of only one parent tended to be involved in nucleolus formation, a phenomenon known as ‘nucleolar dominance’^81–84^. In *A. suecica*, it was observed that the rDNA clusters inherited from *A. thaliana* were silenced^81–87^, and structural changes associated with these clusters were also suggested^88^.

**Fig. 2:**
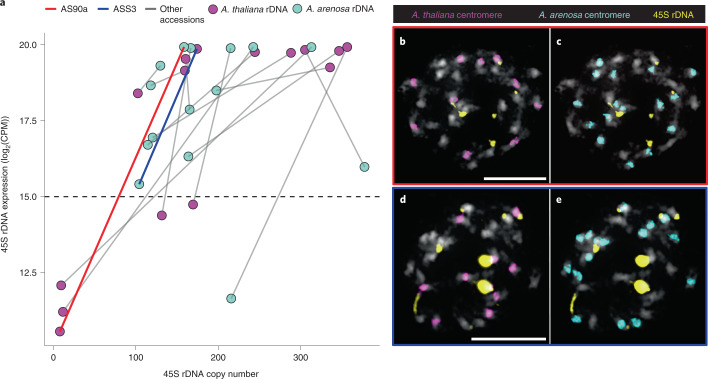
Expression and copy number variation of 45S rDNA in *A. suecica*. **a**, The relationship between expression levels (log_2_(CPM)) and copy number of 45S rDNA shows extensive variation of 45S rDNA copy number and varying direction of ‘nucleolar dominance’. Grey lines connect subgenomes of the same accession. Values above the dashed line are taken as evidence for expression of a particular 45S rDNA allele, as this is above the maximum level of mis-mapping seen in the ancestral species (see Extended Data Fig. 3). **b**,**c**, FISH results of a natural *A. suecica* accession AS90a that has largely lost the rDNA cluster of the *A.thaliana* subgenome (8 copies calculated for the *A. thaliana* 45S rDNA and 159 copies of the *A. arenosa* 45S rDNA). **d**,**e**, FISH results of a natural accession ASS3 that has maintained both ancestral rDNA loci (174 copies calculated for the *A. thaliana* 45S rDNA and 104 copies of the *A. arenosa* 45S rDNA). Scale bars, 10 μm (**b**, **d**).

Given this, we examined the composition and transcription of 45S rDNA repeats. Although the large and highly repetitive 45S rDNA clusters are missing from the genome assembly, we can measure the copy number of *A. thaliana* and *A. arenosa* 45S rRNA genes using sequencing coverage (see Methods). We find that three accessions have experienced massive loss of the *A. thaliana* rDNA loci (Fig. 2a), which was confirmed for one of the accessions (AS90a) by fluorescence in situ hybridization (FISH) analysis (Fig. 2b,c). However, there is massive copy number variation for 45S rRNA genes in *A. suecica* (Fig. 2a), and some accessions (ASS3) have a higher 45S rRNA copy number in *A. thaliana* than in *A. arenosa* (Fig. 2d,e).

We find nucleolar dominance to be variable in *A. suecica* (see Methods and Extended Data Fig. 3). The majority of accessions express both 45S rRNA alleles, five exclusively express *A. arenosa* 45S rRNA and one exclusively expresses *A. thaliana* 45S rRNA (Fig. 2a).

This extensive variation in 45S cluster size and expression is consistent with the intraspecific variation seen in *A. thaliana*^89,90^, and previous observations made in natural *A. suecica*^91^. This suggests that nucleolar dominance may partly be explained by retained ancestral variation. However, the large decrease in rDNA cluster size observed in some accessions may be a consequence of allopolyploidization, as synthetic *A. suecica* sometimes shows loss of 45S rDNA (even as early as the F_1_ stage) that varies between siblings and generations (Extended Data Fig. 3). Elimination of rDNA loci has also been previously observed in synthetic wheat^92^, and loss of rDNA sites has been reported in strawberry^93^.

### No evidence for abnormal transposon activity

The possibility that allopolyploidization leads to a ‘genome shock’ in the form of increased transposon activity has been discussed^27,28,94,95^. Evidence for TE proliferation following hybridization has been found for *Ty3/Gypsy* retrotransposons in hybrid sunflower species^96^, although this may be due to environmental change^97,98^. Analysis of TE expression in F_1_ hybrids between *A. thaliana* and *A. lyrata* found strong correlation to the parent species, and little alteration of repressive chromatin marks^99^—although the F_1_ generation may be too early to study TE misregulation. Here we examine TE dynamics in *A. suecica*.

In *A. suecica* there are almost twice as many annotated transposons in the *A. arenosa* compared to the *A. thaliana* subgenome (66,722 versus 33,420). The difference is likely to be greater given that the *A. arenosa* subgenome assembly is less complete and TE annotation is biased towards *A. thaliana*. Whether the combination of these two genomes has led to increased transposon activity is unknown.

**Fig. 3:**
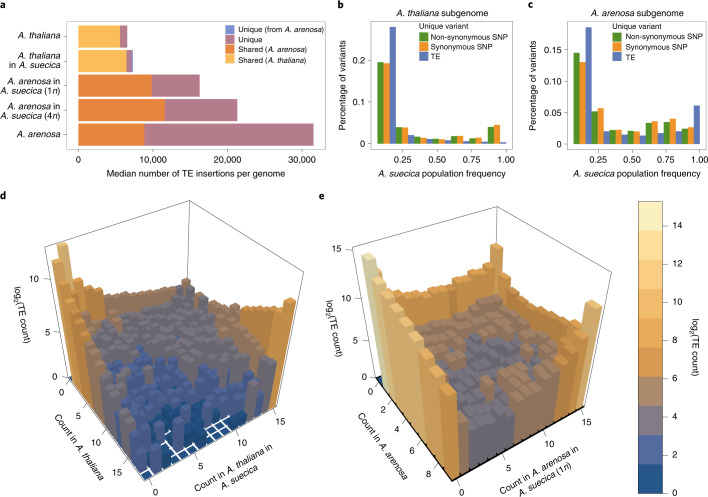
TE dynamics in *A. suecica* reveal no evidence for abnormal transposon activity. **a**, Median TE insertions per genome. As the *A. arenosa* population is an autotetraploid outcrosser, four randomly chosen haploid *A. arenosa* subgenomes of *A. suecica* were combined to make a 4*n* *A. suecica*. *A. suecica* does not show an increase in private TE insertions compared with the ancestral species for either subgenome, and shared TEs constitute a higher fraction of TEs in *A. suecica*, reflecting the strong population bottleneck at its origin. **b**,**c**, Site-frequency spectra of non-synonymous SNPs, synonymous SNPs and TEs in the *A. thaliana* (**b**) and *A. arenosa* (**c**) subgenomes of *A. suecica* suggest that TEs are under purifying selection on both subgenomes. **d**, Three-dimensional histogram of a joint TE frequency spectrum for *A. thaliana* on the *x* axis and the *A. thaliana* subgenome of *A. suecica* on the *y* axis. **e**, Three-dimensional histogram of a joint TE frequency spectrum for *A. arenosa* on the *x* axis and the *A. arenosa* subgenome of *A. suecica* on the *y* axis. **d** and **e** show stable dynamics of private TEs in *A. suecica* and a bottleneck effect on the ancestral TEs (shared) at the origin of the *A. suecica* species.

**Extended Data Fig. 4:**
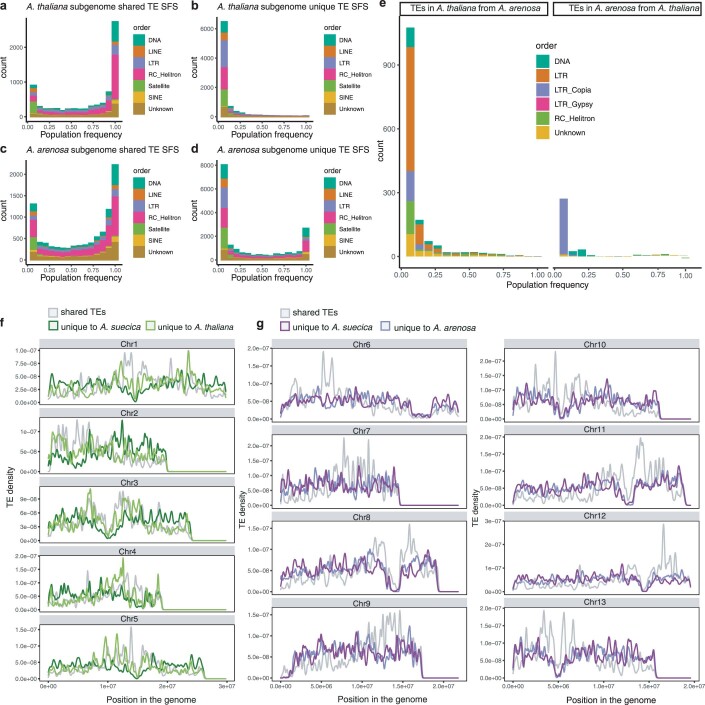
Population frequency and genomic location of transposon polymorphisms. Shared TE SFS for the **a**, *A. thaliana* and **b**, *A. arenosa* subgenome. Private TE SFS for the **c**, *A. thaliana* and **d**, *A. arenosa* subgenome. **e**, TEs ancestrally from *A. arenosa* that are present in the *A. thaliana* subgenome of *A. suecica* and TEs ancestrally from *A. thaliana* that are present in the *A. arenosa* subgenome of *A. suecica*. **f**, Shared TEs in the population between *A. thaliana* and the *A. thaliana* subgenome of *A. suecica*. Shared TEs are likely older than private TEs and are enriched around the pericentromeric regions in the *A. thaliana* subgenome. Private TEs are enriched in the chromosomal arms for both species, where protein-coding gene density is higher (Fig. 1b). **g** as in **f** but examining TEs in the population of *A. arenosa* and the *A. arenosa* part of *A. suecica*. Note the region between 5 and 10 on chromosome 2 was not included in the analysis as this region shows synteny with an unplaced contig.

The *A. thaliana* subgenome contains around 3,000 more annotated transposons than the TAIR10 *A. thaliana* reference genome but could reflect a greater number of transposons in the *A. thaliana* ancestors rather than increased transposon activity in *A. suecica*. To investigate TE activity, unique TE jumps that occurred after the species separated are needed. We used the software PoPoolationTE2^100^ to call presence–absence variation on a population level using 15 natural *A. suecica* accessions, 18 *A. thaliana* accessions genetically close to *A. suecica*, and 9 *A. arenosa* lines. Of the 24,569 insertion polymorphisms in the *A. thaliana* subgenome, 8,767 were shared between *A. thaliana* and *A. suecica*, 7,196 were unique to *A. thaliana* and 8,606 were unique to *A. suecica*. Of the 115,336 insertion polymorphisms in the *A. arenosa* subgenome, 13,177 were shared with *A. arenosa*, 83,964 were unique to *A. arenosa* and 18,195 were unique to *A. suecica* (Supplementary Data 1a,b and Extended Data Fig. 4). Considering the number of transposons per individual genome (Fig. 3a), most transposon insertions in a typical *A. thaliana* subgenome are also found in *A. thaliana*. The slightly higher transposon load in the *A. thaliana* subgenome is probably due to the population bottleneck. Notably, the number of unique insertions is not higher in the *A. thaliana* subgenome, suggesting no increase in transposon activity.

Turning to the *A. arenosa* subgenome, we see that *A. suecica* contains only about half the number of transposons of *A. arenosa* on average (Fig. 3a). However, *A*. arenosa is an outcrossing tetraploid, therefore four randomly chosen *A. arenosa* subgenomes of *A. suecica* were used in the comparison (‘*A. arenosa* in *A. suecica* (4n)’ in Fig. 3a). This largely accounts for the observed difference, but there are still fewer transposons in *A. suecica*. A population bottleneck is likely to explain the difference, although decreased transposon activity in *A. suecica*, related to its transition to selfing^101^, cannot be ruled out.

**Extended Data Fig. 5:**
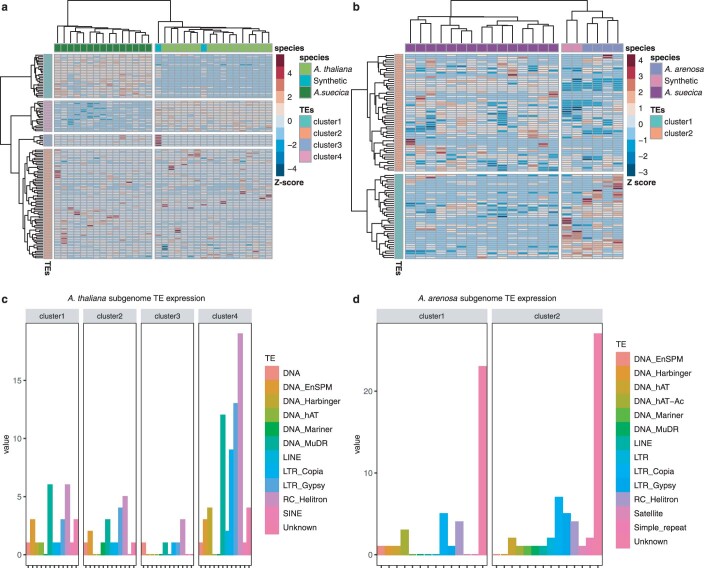
Transposable element expression analysis. Patterns of TE expression in natural and synthetic *A. suecica* show that allopolyploidy is not accompanied by an overall upregulation in TE expression as predicted by the ‘genome shock’ hypothesis. **a**, Heat map of TE expression for the *A. thaliana* subgenome of *A. suecica* (dark green) synthetic *A. suecica* (cyan) and *A. thaliana* (light green). **b**, Heat map of TE expression for the *A. arenosa* subgenome of *A. suecica* (dark purple) synthetic *A. suecica* (pink) and *A. arenosa* (light purple). **c** and **d** the breakdown of TE families expressed in each cluster, with helitrons being the most abundant class on the *A. thaliana* subgenome and TEs of an unknown family being the most abundant in the *A. arenosa* subgenome.

In summary, we see no evidence for a burst of transposon activity in *A. suecica*, a conclusion supported by the analysis of transposon expression for *A. suecica*, which shows no upregulation relative to the ancestor species (Extended Data Fig. 5). The frequency distribution of polymorphic transposon insertions unique to *A. suecica* is heavily skewed towards zero, likely because of purifying selection as the distribution is more similar to that of non-synonymous than synonymous single-nucleotide polymorphisms (SNPs) (Fig. 3b,c). However, for both subgenomes, *A. suecica* also contains a large number of fixed or nearly fixed insertions that are present in the ancestral species at a lower frequency (Fig. 3d,e). These are likely to have reached high frequency as a result of a bottleneck. Shared transposons are enriched in the pericentromeric regions, while unique transposon insertions, generally at low frequency, are more uniformly distributed across the genome, which is consistent with strong selection against transposon insertions in the gene-dense chromosome arms^102,103^ (Extended Data Fig. 4).

An interesting subset of recent transposon insertions unique to *A. suecica* are those that have jumped between the subgenomes. We searched for full-length transposon copies that are present in both subgenomes of *A. suecica* and assigned the transposon sequences to the *A. thaliana* or the *A. arenosa* ancestral genome (see Methods). We were able to assign 15 and 56 transposon sequences as belonging to the *A. thaliana* and *A. arenosa* ancestral genome, respectively. Using these sequences, we searched our transposon polymorphisms and identified 1,515 *A. arenosa* transposon polymorphisms on the *A. thaliana* subgenome, and 496 *A. thaliana* transposon polymorphisms on the *A. arenosa* subgenome. Like other private polymorphisms, these are skewed towards rare frequencies, and are uniformly distributed across the (sub-)genome. Most of the transposons that have jumped into the *A. thaliana* subgenome are helitron and long terminal repeat (LTR) elements (Extended Data Fig. 4). LTR elements also make up most of the *A. thaliana* transposons segregating in the *A. arenosa* subgenome. Three times as many transposon jumps from *A. arenosa* to *A. thaliana* than vice versa is notable, and suggests higher transposon activity in the *A. arenosa* subgenome, but we must consider differences in genome size and transposon number. If no differences in activity exist, we would expect the number of jumps to be proportional to the number of potential source elements and the size of the target genome. As the *A. arenosa* subgenome contains roughly twice as many transposons as the *A. thaliana* subgenome and is about 20% larger, we expect a 1.7-fold difference, not a three-fold one.

In conclusion, transposon activity in *A. suecica* appears to be governed by the same processes as in the ancestral species.

### No global dominance in expression between the subgenomes

Over time the traces of polyploidy are erased through an evolutionary process referred to as fractionation or re-diploidization^104–108^. Analyses of retained homeologues in ancient allopolyploids such as *A. thaliana*^109^, maize^55^, *B. rapa*^54^ and *Gossypium raimondii*^110^ have revealed that one ‘dominant’ subgenome remains more intact, with more highly expressed homeologues compared to the ‘submissive’ genome(s)^109^. This pattern of biased fractionation has not been observed in ancient autopolyploids^111,112^, and is believed to be allopolyploid-specific.

Studying genome expression dominance in allopolyploids is useful for understanding or predicting which of the subgenomes will likely be refractory to, and which will likely experience this fractionation process more, over time^55^. Subgenome dominance in expression has been reported for a number of recent allopolyploids such as strawberry^6^, peanut^8^, *Spartina*^68^*, T. miscellus*^113^, monkeyflower^17^ and synthetic *B. napus*^114^. However, some allopolyploids display even subgenome expression: *Capsella bursa-pastoris*^10,12^, *Trifolium* *repens*^13^, *Arabidopsis kamachatica*^70^ and *Brachypodium hybridum*^14^.

Subgenome dominance is linked to differences in transposon content^6^ and/or large genetic differences between subgenomes^115^. This makes *A. suecica*, with 6 Ma divergence between the gene-dense *A. thaliana* and the transposon-rich *A. arenosa*, a promising candidate to study this phenomenon. Previous reports on subgenome dominance in *A. suecica* are conflicting, suggesting a bias to either the *A. thaliana*^116^ or the *A. arenosa*^117^ subgenome.

**Extended Data Fig. 6:**
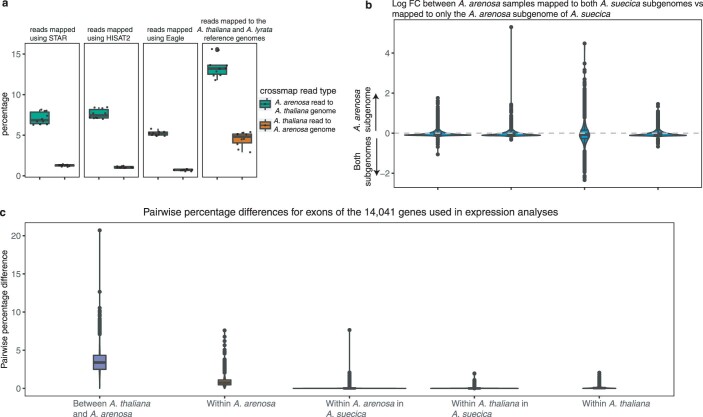
Cross-mapping of RNA-seq short reads. **a**, Box plots of cross-mapping RNA short reads. This was examined by mixing reads in-silico between *A. thaliana* and *A. arenosa*. On average ~6% of *A. arenosa* reads map to *A. thaliana* subgenome instead of the *A. arenosa* subgenome, and ~1% vice versa. Mapping these reads to the combined reference genomes of *A. thaliana* and *A. lyrata* (box plot 4 in **a**) shows that reads map more precisely to the *A. suecica* reference and that cross-mapping is not due to unreported HE. **b**, LogFC of log_2_(CPM) read counts for *A. arenosa* (CPM of *A. arenosa* subgenome genes when reads are mapped only to *A. arenosa* subgenome of *A. suecica*/CPM of *A. arenosa* subgenome genes when reads are mapped to the full genome) show only a small effect of mapping strategy to estimate gene expression on the *A. arenosa* subgenome. **c**, Pairwise percentage differences (π) for each group measured for the exons of the 14,041 genes in the expression analysis. High levels of π in *A. arenosa* overlaps with the distribution of π between *A. thaliana* and *A. arenosa*. This explains why there is more cross-mapping for *A. arenosa* than for *A. thaliana* in **a** Importantly, lower π within *A. suecica* for both subgenomes means that measurements for subgenome dominance are not biased by cross-mapping, as we expect less cross-mapping since the distribution of π overlaps less with π between *A. thaliana* and *A. arenosa*.

To investigate the evolution of gene expression in *A. suecica*, we generated RNA sequencing (RNA-seq) data for 15 natural *A. suecica* accessions, 15 closely related *A. thaliana* accessions, 4 *A. arenosa* individuals, a synthetically generated *A. suecica* from a lab cross (the second and third hybrid generations) and the parental lines of this cross. Each sample had 2–3 biological replicates (Supplementary Data 2). On average, we obtained 10.6 million raw reads per replicate, of which 7.6 million reads were uniquely mapped to the *A. suecica* reference genome and 14,041 homeologous gene pairs (see Methods). On average, around 1% of A. thaliana and around 6% of *A. arenosa* RNA reads cross-mapped between the subgenomes of *A. suecica*. The approximately 6% of cross-mapping in *A. arenosa* is likely to be because of the high level of polymorphism in this outcrossing species. However, diversity within *A. suecica* is massively lower, meaning that transcripts from the *A. arenosa* subgenome will probably map correctly (see Methods and Extended Data Fig. 6).

**Fig. 4:**
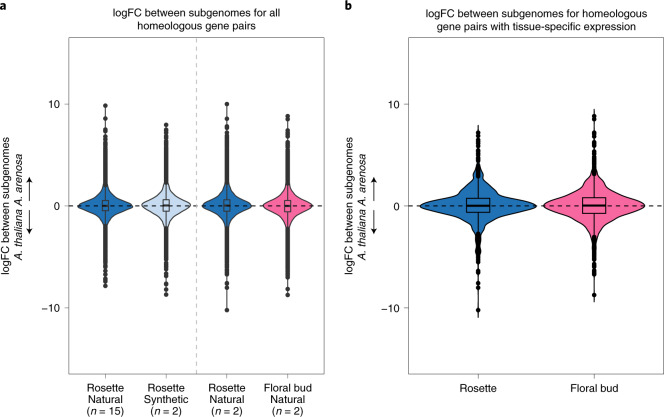
Patterns of gene expression between the subgenomes of *A. suecica* in rosettes and floral buds. **a**, Violin plots of the mean log fold change (logFC) between the subgenomes for the 15 natural *A. suecica* accessions and 2 synthetic lines for whole rosettes. The centre line is the 50th percentile or median. The box limits represent the interquartile range. The whiskers represent the largest and smallest value within 1.5 times the interquartile range above and below the 75th and 25th percentile, respectively. Mean log fold change for the two accessions (ASS3 and AS530) for which transcriptome data for both whole rosettes and flower buds were available. All the distributions are centred around zero, suggesting even subgenome expression. **b**, Violin plots for the mean log fold change between the subgenomes for gene pairs with tissue-specific expression in at least one member of the pair.

**Extended Data Fig. 7:**
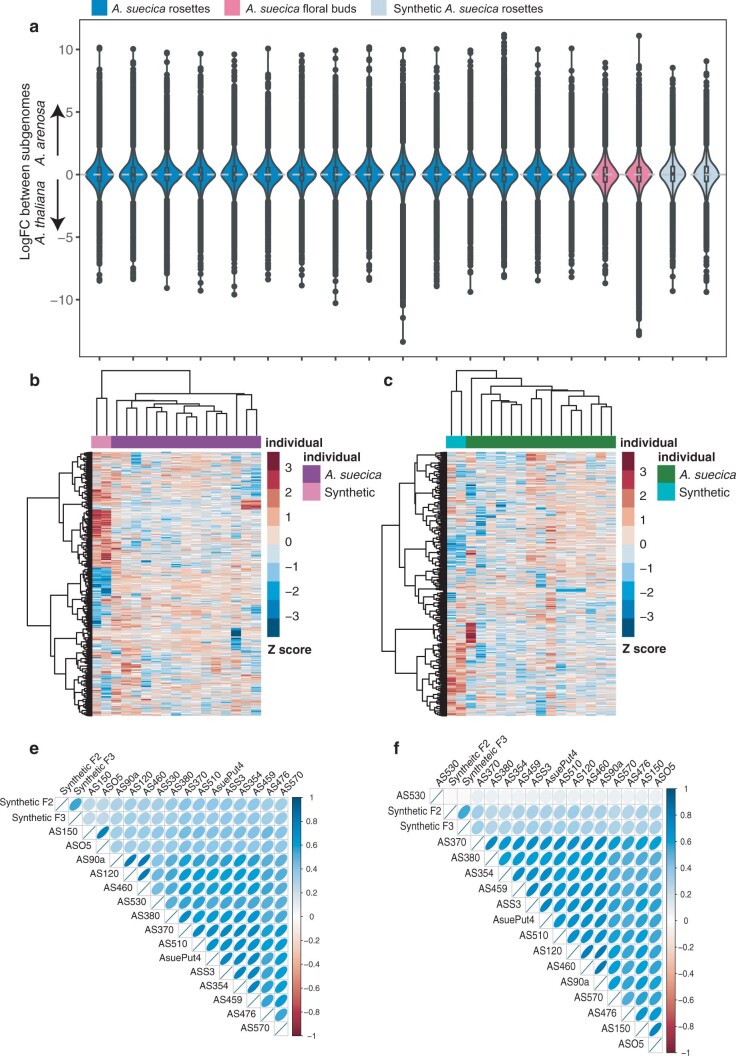
Expression differences between subgenomes in natural and synthetic *A. suecica*. The distribution of expression differences across homeologous gene pairs in natural and synthetic *A. suecica*. **b**, A heat map of expression for genes in the top 5% biased toward the *A. arenosa* subgenome. The gene must be in the 5% quantile for at least 1 accession. **c**, The same as in **b** but for the *A. thaliana* subgenome. Correlations of log fold change for genes in the tails of the distribution (top 5% quantile) for the *A. arenosa* subgenome **d** and the *A. thaliana* subgenome **e**.

Examining expression differences between homeologous gene pairs, we found no general bias towards a subgenome of *A. suecica* (that is, the mean log fold change is 0), for any sample or tissue, including synthetic *A. suecica* (Fig. 4a and Extended Data Fig. 7). This suggests that the expression differences between the subgenomes have not changed systematically through polyploidization, and is in contrast to previous studies, which reported a bias towards the *A. thaliana*^116^ or the *A. arenosa*^117^ subgenome, likely because RNA-seq reads were not mapped to an appropriate reference genome.

The set of genes that show large expression differences between the subgenomes are not biased towards any particular GO category, and are not consistent between accessions and individuals (Fig. 4b and Extended Data Fig. 7). This suggests that many large subgenome expression differences are due to genetic polymorphisms within *A. suecica* rather than fixed differences between the ancestral species.

Levels of expression dominance were reported to vary across tissues in natural *C. bursa-pastoris*^11^ and resynthesized cotton^118^. To test whether expression dominance can vary for tissue-specific genes, we examined homeologous gene pairs in which at least one gene in the pair showed tissue-specific expression, in whole rosettes and floral buds. We do not find evidence for dominance between subgenomes in tissue-specific expression (Fig. 4b). A total of 897 genes with significant expression in whole rosettes for both homeologues showed GO overrepresentation that included both photosynthesis- and chloroplast-related functions (Supplementary Table 1). This result suggests that the *A. arenosa* subgenome has established important cyto-nuclear communication with the chloroplast inherited from *A. thaliana*, rather than being silenced. A total of 2,176 gene pairs with floral-bud-specific expression for both homeologues were overrepresented for GO terms related to responses to auxin and jasmonic acid that may reflect early developmental changes in this young tissue (Supplementary Table 1). Although flowers of selfing *A. thaliana* and *A. suecica* are scentless and are much smaller than those of the outcrosser *A. arenosa*^72^, this result suggests that the ‘selfing syndrome’^119^ has not hugely affected the transcriptome of floral buds in *A. suecica* at this stage of development.

In summary, we find no evidence that one subgenome is dominant and contributes more to the functioning of *A. suecica*. On the contrary, homeologous gene pairs are strongly correlated in expression across tissues.

### Evolving gene expression in *A. suecica*

**Fig. 5:**
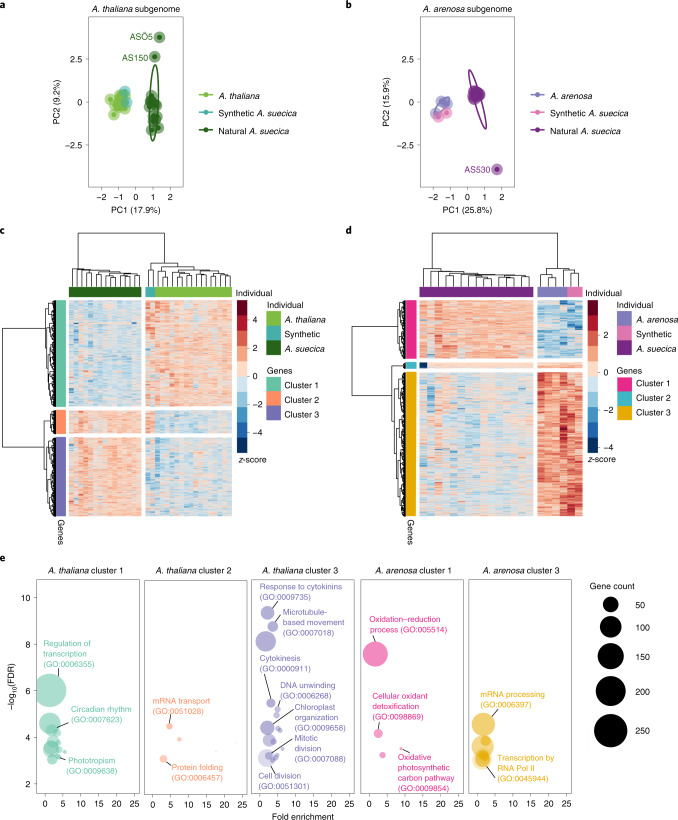
Differential gene expression analysis in *A. suecica*. Patterns of differential gene expression in *A. suecica* support adaptation to the whole-genome duplication for the *A. thaliana* subgenome and adaptation to the new plastid environment for the *A. arenosa* subgenome. **a**, PCA for *A. thaliana* and the *A. thaliana* subgenome of natural and synthetic *A. suecica* lines. Principal component 1 (PC1) separates natural *A. suecica* from the ancestral species and the synthetic lines. **b**, PCA for *A. arenosa* and the *A. arenosa* subgenome of natural and synthetic *A. suecica* lines. PC1 separates natural *A. suecica* from the ancestral species and the synthetic lines, whereas PC2 identifies outlier accessions discussed further below (see Fig. 6). **c**,**d**, Heat map of DEGs for the *A. thaliana* (**c**) and the *A. arenosa* (**d**) subgenome of *A. suecica*. Positive numbers (red colour) indicate higher expression. Genes and individuals have been clustered on the basis of similarity in expression, resulting in the clusters that are discussed in the text. **e**, GO enrichment for each cluster in **c** and **d**. Categories discussed in the text are highlighted. RNA Pol II, RNA polymerase II.

**Extended Data Fig. 8:**
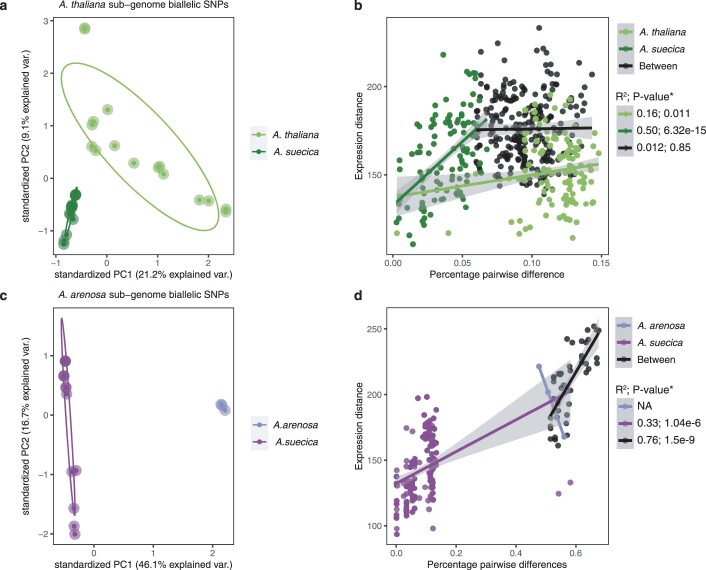
Comparison of genetic and expression distance. **a**, PCA plot of biallelic SNPs in the population of *A. thaliana* and *A. suecica* for the *A. thaliana* subgenome of *A. suecica* (N=345,075 biallelic SNPs), of the analysed 13,647 genes in gene expression in addition to 500 bp up and downstream of each gene sequence **b**, Correlation of 𝜋 (pairwise genetic differences) and expression distance (that is, euclidean distance) for 14,041 genes (*=Bootstrapped 1000 times). **c**, PCA plot of biallelic SNPs in the population of *A. arenosa* (N.B. we had DNA sequencing for only 3 of the 4 accessions used in the expression analysis) and *A. suecica* for the *A. arenosa* subgenome of *A. suecica* (N= 1,761,708 biallelic SNPs), of the analysed 14,041 genes in gene expression in addition to 500 bp up and downstream of each gene sequence **d**, Correlation of Pi (pairwise genetic differences for mapped genomic regions) and expression distance (that is, euclidean distance) for 14,041 genes (*=Bootstrapped 1000 times). *A. arenosa* was too few samples to give reliable correlations and therefore is NA. Grey bars represent the 95 confidence intervals.

The previous section focused on differences in expression between the subgenomes within the same individual. This section will focus on differences between individuals. To provide an overview of expression differences between individuals we performed a principal component analysis (PCA) on gene expression separately for each (sub-)genome. For both subgenomes, the first principal component separates *A. suecica* from the ancestral species and the synthetic hybrid (Fig. 5a,b and Extended Data Fig. 8), suggesting that hybridization does not automatically result in large-scale transcriptional changes, and that gene expression changes in natural *A. suecica* have evolved over time. Given the limited time involved, and the fact that the genes that have changed expression are not random with respect to function (Fig. 5c), we suggest that the first principal component captures *trans*-regulated expression changes in *A. suecica* that are adaptive.

To further characterize expression changes in natural *A. suecica*, we analysed differentially expressed genes (DEGs) on each subgenome compared to the corresponding ancestral species. The total number of DEGs was 4,186 and 4,571 for the *A. thaliana* and *A. arenosa* subgenome, respectively (see Methods and Supplementary Data 2). These genes were clustered on the basis of the pattern of change across individuals (Fig. 5c,d) and GO enrichment analysis was performed for each cluster (Fig. 5e and Supplementary Table 2).

For the *A. thaliana* subgenome, we identified three clusters. Cluster 1 comprised 2,135 genes that showed decreased expression in *A. suecica* compared to *A. thaliana*. These genes are enriched for transcriptional regulation, which may be expected as we are examining DEGs between the species. Enrichments for circadian rhythm function and phototropism may be related to the ecology of *A. suecica* and its post-glacial migration to Fennoscandinavia (Fig. 1a).

**Extended Data Fig. 9:**
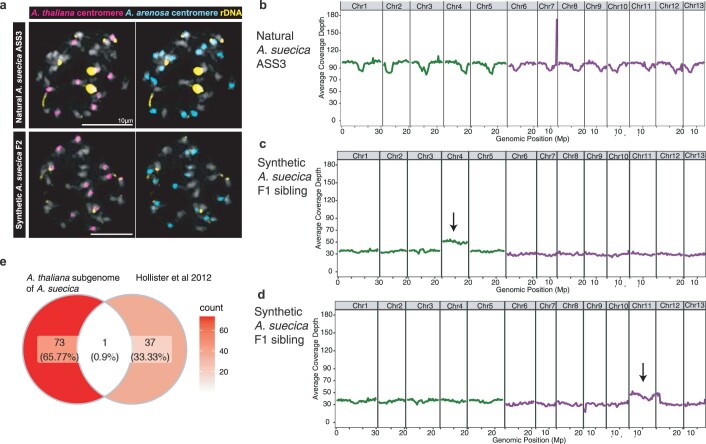
Aneuploidy is frequently observed in synthetic *A. suecica*. **a**, Comparison of FISH analyses of the reference natural *A. suecica* ‘ASS3’ and synthetic *A. suecica*. Synthetic *A. suecica* shows aneuploidy in both subgenomes in the F_2_ generation (gain of one chromosome on the *A. thaliana* subgenome (N=11) and loss of one chromosome on the *A. arenosa* subgenome (N=15)). Natural *A. suecica* shows a stable karyotype **b**, DNA-sequencing coverage in the reference natural *A. suecica* accession ‘ASS3’ **c** and **d**, DNA-sequencing coverage in siblings of F_1_ synthetic *A. suecica* show different cases of aneuploidy (indicated with arrow) in synthetic *A. suecica*, chromosome 4 in **c** and chromosome 11 in **d e** overlap of genes involved in cell division from Fig. 5e and genes previously shown to play a role in the adaptation to autopolyploidy in *A. arenosa*^121^. The little overlap in genes between *A. suecica* and *A. arenosa* highlights that successful meiosis in polyploids is likely a complex trait.

Cluster 2 consisted of 468 genes that are upregulated in both natural and synthetic *A. suecica* relative to *A. thaliana*. These expression changes most likely are an immediate consequence of *trans*-regulation in hybrids. Genes in this cluster are enriched for mRNA transport and protein folding. Adjustment of protein homeostasis has been reported previously in experimentally evolved stable polyploid yeast^120^. Notably, the synthetic lines used in the expression analysis did not show signs of aneuploidy (Extended Data Fig. 9).

Cluster 3 consisted of 1,583 genes that are upregulated in *A. suecica* compared to *A. thaliana*, and several of the enriched GO categories, such as microtubule-based movement, cytokinesis, meiosis and cell division, suggest that the *A. thaliana* subgenome of *A. suecica* is adapting to polyploidy at a cellular level. Selection for this seems likely given that aneuploidy is frequent in synthetic *A. suecica* (Extended Data Fig. 9), while natural *A. suecica* has a stable conserved karyotype. Independent evidence for adaptation to polyploidy via modifications of the meiotic machinery in the other ancestor of *A. suecica*, *A. arenosa*, also exists^23,121,122^, although we see very little overlap in the genes involved (Extended Data Fig. 9). The nature of these changes in the *A. thaliana* subgenome of *A. suecica* will require further investigation, but we note the enrichment (see Methods and Supplementary Data 2) of MYB family transcription factor binding sites^123^ in cluster 3.

For the *A. arenosa* subgenome, we also found three clusters of DEGs (Fig. 5d) with GO enrichment for two clusters (Fig. 5e and Supplementary Table 2). Cluster 1 consisted of 1,278 genes that are upregulated in natural *A. suecica* compared to *A. arenosa* and synthetic *A. suecica*. We find enrichment for plastid-related functions that may be due to selection on the *A. arenosa* subgenome to restore communication with the maternally inherited plastids from *A. thaliana*. Twelve out of a total of 69 genes with structural evidence for direct plastid–nuclear interactions in *A. thaliana* overlap our genes in Cluster 1 using CyMIRA^124^ (*P* = 0.0072; one-sided Fisher’s exact test; Supplementary Data 2). Cluster 3 consists of 3,166 genes that are downregulated in *A. suecica* compared to *A. arenosa* and synthetic *A. suecica*. These genes were enriched for mRNA processing and epigenetic regulation of gene expression (Supplementary Table 2), and for positive regulation of transcription by RNA polymerase II, which suggests differences in the epigenetic regulation of expression between *A. arenosa* and *A. suecica*. Cluster 2 (127 genes), finally, did not have a GO overrepresentation and showed an intriguing pattern that will be discussed in the next section.

### Homeologous exchange contributes to variation in gene expression

**Fig. 6:**
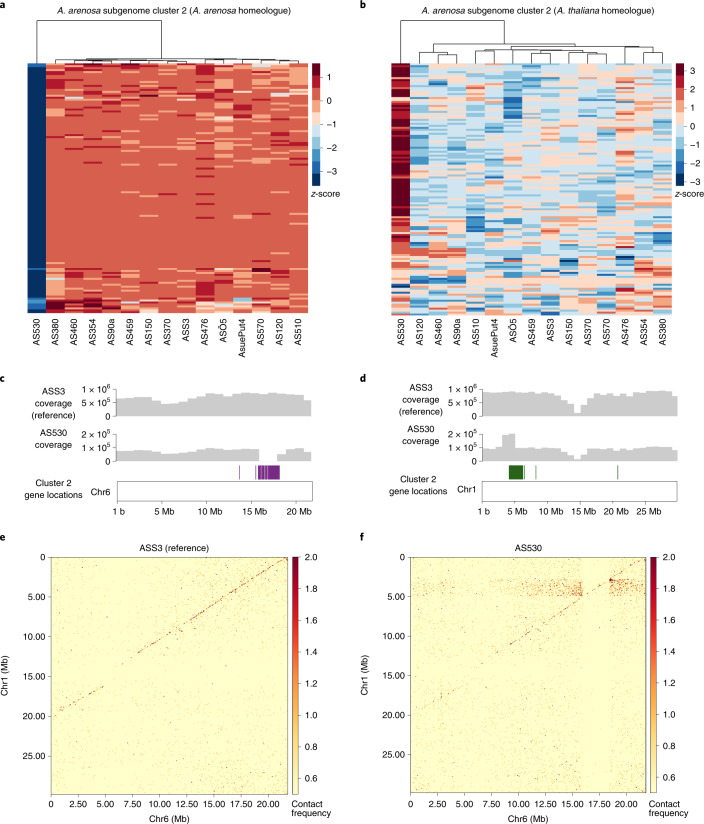
Homeologous exchange contributes to expression variance within *A. suecica*. **a**, Cluster 2 of Fig. 5d explains the outlier accession AS530, which is not expressing a cluster of genes on the *A. arenosa* subgenome. **b**, Homeologous genes of this cluster on the *A. thaliana* subgenome of *A. suecica* show the opposite pattern and are more highly expressed in AS530 compared to the rest of the population. **c**, Of the 122 genes from cluster 3, 97 are close to each other on the reference genome but appear to be deleted in AS530 on the basis of sequencing coverage. **d**, The *A. thaliana* subgenome homeologues have twice the DNA coverage, suggesting that they are duplicated. **e**,**f**, Hi-C data show (spurious) interchromosomal contacts at 25 kb resolution between chromosome 1 and chromosome 6 around the break point of the cluster of 97 genes in AS530 (**f**) but not in the reference accession ASS3 (**e**).

**Extended Data Fig. 10:**
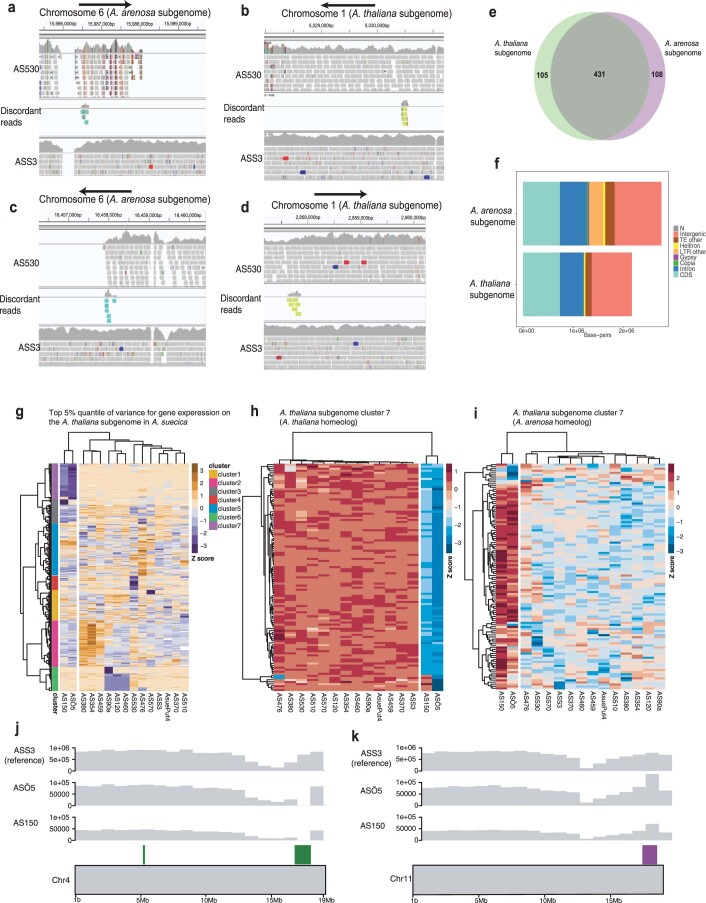
Evidence of HE in *A. suecica*. Reads mapped to the beginning of the HE event in chromosome 6 (~ 15.9 Mb) in ‘AS530’. Arrows point to the direction of the break. Discordant reads map between the *A. arenosa* subgenome on chromosome 6 and the read pair maps to the homeologous chromosome 1 on the *A. thaliana* subgenome (~5 Mb) in **b**. The end of the HE event in chromosome 6 (~18.4 Mb). Discordant reads map between the *A. arenosa* subgenome in **c** and the read pair maps to chromosome 1 (~2.8 Mb) on the *A. thaliana* subgenome in **d**. **e**, Gene counts between the syntenic regions. 431 have a 1:1 relationship, 108 genes are specific to the *A. arenosa* subgenome and 105 genes are specific to the *A. thaliana* subgenome. **f**, Composition of the syntenic regions between the two subgenomes. **g**, The top 5% quantiles (N=702) for variation in gene expression for the *A. thaliana* subgenome shows in cluster 7 (N=111) the two outlier accessions (AS150 and ASÖ5) are expressing genes differently to the rest of the population. **h**, Homeologous genes of this cluster on the *A. thaliana* subgenome of *A. suecica* show that these genes are not expressed in these two accessions while **i** shows they are upregulated in ‘AS150’ and ‘ASÖ5’. **j** and **k** 101/111 genes in cluster 7 are located on chromosome 4 in close proximity to each other on the *A. thaliana* subgenome of the *A. suecica* reference genome and appear to be deleted in AS5Ö5 and AS150. The *A. arenosa* subgenome homeologues (located on chromosome 11) have twice the DNA coverage, suggesting they are duplicated, in agreement with expectations of HE event.

The second principal component for gene expression identified three outlier accessions of *A. suecica*: two for the *A. thaliana* subgenome (Fig. 5a) and one for the *A. arenosa* subgenome (Fig. 5b). While closely examining the latter accession, AS530, we realized that it is responsible for the cluster of genes with distinct expression patterns but no GO enrichment (Fig. 5d, Cluster 2). Genes from this cluster were significantly downregulated on the *A. arenosa* subgenome (Fig. 6a) and upregulated on the *A. thaliana* subgenome (Fig. 6b)—for AS530 only. The observation that 104 of the 127 genes (Extended Data Fig. 10) in the cluster are located in close proximity in the genome pointed to a structural rearrangement. The lack of DNA-sequencing coverage on the *A. arenosa* subgenome around these 104 genes, and the doubled coverage for their homeologues on the *A. thaliana* subgenome, suggested a homeologous exchange (HE) event resulting in AS530 carrying four copies of the *A. thaliana* subgenome and zero copies of the *A. arenosa* subgenome for this approximately 2.5 Mb region of the genome (Fig. 6c). This was further supported by Hi-C data, which showed clear evidence for interchromosomal contacts between *A. thaliana* subgenome chromosome 1 and *A. arenosa* subgenome chromosome 6 around the break points of the putative HE in AS530 (Fig. 6d,e), and by multiple discordant paired-end reads at the break points between the homeologous chromosomes, which independently support the HE event (Extended Data Fig. 10).

We examined the two outlier *A. suecica* accessions for the *A. thaliana* subgenome (Fig. 5a; AS150 and ASÖ5) and found that they probably share a single HE event in the opposite direction (four copies of the *A. arenosa* subgenome and no copies of the *A. thaliana* subgenome for a region of around 1.2 Mb in size; see Extended Data Fig. 10). This demonstrates that HE occurs in *A. suecica* and contributes to the intraspecific variation in gene expression (Fig. 5a,b). HE in allopolyploids is a main source of diversity, causing extensive phenotypic changes^9,125^. However, the majority of HEs are probably deleterious as they will lead to gene loss: although the *A. thaliana* and *A. arenosa* genomes are largely syntenic, AS530 is missing 108 genes (Extended Data Fig. 10) that are only present on the *A. arenosa* subgenome segment that has been replaced by the homeologous segment from the *A. thaliana* subgenome, and AS150 and ASÖ5 are missing 53 genes that were only present on the *A. thaliana* subgenome.

## Discussion

This study has focused on the process of polyploidization in a natural allotetraploid species, *A. suecica*. Its ancestral species, *A. thaliana* and *A. arenosa*, differ substantially in genome size, chromosome number, ploidy, mating system and ecology.

Our main conclusion from this study is that the polyploid speciation leading to *A. suecica* appears to have been a gradual process rather than some kind of ‘event’. We confirmed previous results that genetic polymorphism is largely shared with the ancestral species, demonstrating that *A. suecica* did not originate through a single unique hybridization event, but rather through multiple crosses^20^. We also find no evidence for a ‘genome shock’, in the sense of major genomic changes linked to structural and functional alterations, which has often been suggested to accompany polyploidization and hybridization. Specifically, the genome has not been massively rearranged, transposable elements are not out of control and there is no subgenome dominance in expression. On the contrary, we find evidence of genetic adaptation to ‘stable’ life as a polyploid, in particular changes to the meiotic machinery and in interactions with the plastids. These findings made in natural (but not synthetic) *A. suecica*, together with the observation that experimentally generated *A. suecica* is often unviable and does exhibit evidence of genome rearrangements, similar to the young allopolyploid species in *Tragopogon* and monkeyflower, suggest that the most important bottleneck in polyploid speciation may be selective. If this is true, domesticated polyploids may not always be representative of natural polyploidization. Darwin famously argued that evolution is gradual^126^—we suggest that natural polyploids are no exception from this, but note that many more species will have to be studied before it is possible to draw general conclusions.

## Methods

### PacBio sequencing of *A. suecica*

We used genomic DNA from whole rosettes of one *A. suecica* (ASS3) accession to generate PacBio sequencing data. DNA was extracted using a modified PacBio protocol for preparing *Arabidopsis* genomic DNA for size-selected approximately 20-kb SMRTbell libraries. In brief, whole-genomic DNA was extracted from 32 g of 3–4-week-old plants, grown at 16 °C and subjected to a 2-day dark treatment. This generated 23 μg of purified genomic DNA with a fragment length of more than 40 kb for *A. suecica*. We assessed DNA quality with a Qubit fluorometer and a Nanodrop analysis and ran the DNA on a gel to visualize fragmentation. Genomic libraries and single-molecule real-time (SMRT) sequence data were generated at the Functional Genomics Center Zurich (FGCZ). The PacBio RSII instrument was used with P6-C4 chemistry and an average movie length of 6 hours. A total of 12 SMRT cells were processed, generating 16.3 Gb of DNA bases with an N50 read length of 20 kb and median read length of 14 kb. Using the same genomic library, an additional 3.3 Gb of data was generated by a PacBio Sequel instrument at the Vienna BioCenter Core Facilities (VBCF), with a median read length of 10 kbp.

### *A. suecica* genome assembly

To generate the *A. suecica* assembly we first used FALCON^127^ (v.0.3.0) with a length cut-off for seed reads set to 1 kb in size. The assembly produced 828 contigs with an N50 of 5.81 Mb and a total assembly size of 271 Mb. In addition, we generated a Canu^128^ (v.1.3.0) assembly using default settings, which resulted in 260 contigs with an N50 of 6.65 Mb and a total assembly size of 267 Mb. Then we merged the two assemblies using the software quickmerge^129^. The resulting merged assembly consisted of 929 contigs with an N50 of 9.02 Mb and a total draft assembly size of 276 Mb. We polished the assembly using Arrow^130^ (smrtlink release 5.0.0.6792) and Pilon (v.1.22). For Pilon^131^, 100 bp (with PCR duplicates removed), and a second PCR-free 250 bp, Illumina paired-end reads were used that had been generated from the reference *A. suecica* accession ASS3.

### PacBio sequencing of *A. arenosa*

A natural Swedish autotetraploid *A. arenosa* accession, Aa4, was inbred in a lab for two generations to reduce heterozygosity. We extracted whole-genomic DNA from 64 g of 3-week-old plants in the same way as described for *A. suecica*, generating 50 μg of purified genomic DNA with fragment sizes longer than 40 kb in length. The *A. arenosa* genomic libraries and SMRT sequence data were generated at the VBCF. A PacBio Sequel instrument was used to generate a total of 22 Gb of data from five SMRT cells, with an N50 of 13 kb and median read length of 10 kb. In addition, two runs of Oxford Nanopore sequencing were carried out at the VBCF, producing 750 Mb in 180,000 reads (median 5 kb and 2.6 kb; N50 8.7 and 6.7 kb, respectively).

### Assembly of autotetraploid *A. arenosa*

We assembled a draft contig assembly for the autotetraploid *A. arenosa* accession Aa4 using FALCON (v.0.3.0) as for *A. suecica*. The assembly produced 3,629 contigs with an N50 of 331 kb, a maximum contig size of 2.5 Mb and a total assembly size of 461 Mb. The assembly size is greater than the calculated haploid size of 330 Mb using fluorescence-activated cell sorting (FACS) (see Extended Data Fig. 1), probably because of the high levels of heterozygosity in *A. arenosa*. The resulting assembly was polished as described for *A. suecica*.

### Hi-C tissue fixation and library preparation

To generate physical scaffolds for the *A. suecica* assembly we generated proximity-ligation Hi-C sequencing data. We collected approximately 0.5 g of tissue from 3-week-old seedlings of the same reference *A. suecica* accession. Freshly collected plant tissue was fixed in 1% formaldehyde. Cross-linking was stopped by the addition of 0.15 M glycine. The fixed tissue was ground to a powder in liquid nitrogen and suspended in 10 ml nuclei isolation buffer. Nuclei were digested by adding 50 U DpnII and the digested chromatin was blunt-ended by incubation with 25 μl of 0.4 mM biotin–14-dCTP and 40 U of Klenow enzyme. T4 DNA ligase (20 U) was then added to start proximity ligation. The extracted DNA was sheared by sonication with a Covaris S220 to produce 250–500-bp fragments. This was followed by size fractionation using AMPure XP beads. Biotin was then removed from unligated ends. DNA fragments were blunt-end-repaired and adaptors were ligated to the DNA products following the NEBNext Ultra II RNA Library Prep Kit for Illumina.

To analyse structural rearrangements, we collected tissue for one other natural *A. suecica* (AS530), one *A. thaliana* accession (6978), one *A. arenosa* (Aa6) and one synthetic *A. suecica* (F3). Each sample had two replicates. We collected tissue and prepared libraries in the same manner as described above. Illumina reads (125-bp paired-end) were mapped using HiCUP^132^ (v.0.6.1).

### Reference-guided scaffolding of the *A. suecica* genome with LACHESIS

We sequenced 207 million pairs of 125-bp paired-end Illumina reads from the Hi-C library of the reference accession ASS3. We mapped reads using HiCUP (v.0.6.1) to the draft *A. suecica* contig assembly. This resulted in around 137 million read pairs with a unique alignment.

Setting an assembly threshold of ≥1 kb in size, contigs of the draft *A. suecica* assembly were first assigned to the *A. thaliana* or *A. arenosa* subgenome. To do this, we used nucmer from the software MUMmer^133^ (v.3.23) to perform whole-genome alignments. We aligned the draft *A. suecica* assembly to the *A. thaliana* TAIR10 reference and to our *A. arenosa* draft contig assembly, simultaneously. We used the MUMer command dnadiff to produce 1-to-1 alignments. As the subgenomes are only around 86% identical, the majority of contigs could be conclusively assigned to either subgenome by examining how similar the alignments were. Contigs that could not be assigned to a subgenome on the basis of percentage identity were examined manually, and the length of the alignment was used to determine subgenome assignment.

Finally, we used the software LACHESIS^134^ (v.1.0.0) to scaffold our draft assembly, using the reference genomes of *A. thaliana* and *A. lyrata as* a guide to assist with scaffolding the contigs (we used *A. lyrata* here instead of our draft *A. arenosa* contig assembly, as *A. lyrata* is a chromosome-level assembly). This produced a 13-scaffold chromosome-level assembly for *A. suecica*.

### Construction of the *A. suecica* genetic map

We crossed the natural *A. suecica* accession AS150 with the reference accession ASS3. The cross was uni-directional with AS150 as the maternal and ASS3 as the paternal plant. A total of 192 F_2_ plants were collected. We multiplexed the samples on 96-well plates using 75-bp paired-end reads and generated data of 1–2× coverage per sample. Samples were mapped to the repeat-masked scaffolds of the reference *A. suecica* genome using BWA-MEM^135^ (v.0.7.15). SAMtools^136^ (v.0.1.19) was used to filter reads for proper pairs and a minimum mapping quality of 5 (-F 256 -f 3 -q 5). We called variants directly from SAMtools mpileup giving a total of 590,537 SNPs. We required sites to have non-zero coverage in a minimum of 20 individuals and filtered SNPs to have frequency between 0.45-0.55 in our F_2_ population. We removed F_2_ individuals that did not have genotype calls for more than 90% of the data. This resulted in 183 individuals with genotype calls for 334,257 SNPs.

We applied a hidden Markov model implemented in the R package HMM^137^ to classify SNPs as homozygous or heterozygous for each of our F_2_ lines. We then divided the genome into 500-kb non-overlapping windows, and classified each window as homozygous or heterozygous. This was done per chromosome and the resulting file for each chromosome and their markers were processed in the R package qtl^138^, to generate a genetic map. Markers genotyped in fewer than 100 F_2_ individuals were excluded from the analysis. Linkage groups were assigned with a minimum log of the odds (LOD) score of 8 and a maximum recombination fraction of 0.35. We defined the final marker order by the best LOD score and the lowest number of crossover events.

We corrected the erroneous placement of a contig at the beginning of chromosome 1 of the *A. arenosa* subgenome. The misplaced contig was relocated from chromosome 1 to the pericentromeric region of chromosome 2 of the *A. arenosa* subgenome in *A. suecica*. Chromosome 2 of the *A. thaliana* subgenome of *A. suecica* was previously shown to be largely devoid of intraspecific variation, resulting in few markers for this chromosome. Therefore, this chromosome-scale scaffold was assembled by the manual inspection of 3D-proximity information based on our Hi-C sequencing using the software Juicebox^139^.

### Gene prediction and annotation of the *A. suecica* genome

We combined de novo and evidence-based approaches to predict protein-coding genes. For de novo prediction, we trained AUGUSTUS^140^ on the set of conserved single-copy genes using BUSCO^141^ separately on *A. thaliana* and *A. arenosa* subgenomes of *A. suecica*. The evidence-based approach included both homology to the protein sequences of the ancestral species and the transcriptome of *A. suecica*. We aligned the peptide sequences from the TAIR10 *A. thaliana* assembly to the *A. thaliana* subgenome of *A. suecica*, while the peptides from *A. lyrata* annotation^142^ (Alyrata_384_v2.1) were aligned to the *A. arenosa* subgenome of *A. suecica* using GenomeThreader^143^ (v.1.7.0). We mapped the RNA-seq reads from the reference accession of *A. suecica* (ASS3) from the rosettes and flower bud tissues to the reference genome using TopHat^144^ and generated intron hints from the split reads using the bam2hints extension of AUGUSTUS. We split the alignment into *A. thaliana* and *A. arenosa* subgenomes and assembled the transcriptome of *A. suecica* for each subgenome separately in the genome-guided mode with Trinity^145^ (v.2.6.6). Separately for each of the subgenomes, we filtered the assembled transcripts using a transcripts per million (TPM) cut-off set to 1, collapsed similar transcripts using CD-HIT^146,147^ with sequence identity set to 90%, and chose the longest open reading frame from the six-frame translation. We then aligned the proteins from *A. thaliana* and *A. arenosa* parts of *A. suecica* to the corresponding subgenomes using GenomeThreader (v.1.7.0). We ran AUGUSTUS using retrained parameters from BUSCO and merged hints from all three sources, these being: (1) intron hints from *A. suecica* RNA-seq; (2) homology hints from ancestral proteins; and (3) hints from *A. suecica* proteins.

RepeatModeler^148^ (v.1.0.11) was used to build a de novo TE consensus library for *A. suecica* and identify repetitive elements based on the genome sequence. Genome locations for the identified TE repeats were determined by using RepeatMasker^149^ (v.4.0.7) and filtered for full-length matches using a code described previously^150^. Helitrons are the most abundant TE family in both subgenomes.

### Synthetic *A. suecica* lines

To generate synthetic *A. suecica* we crossed a natural tetraploid *A. thaliana* accession (6978, also known as Wa-1) to a natural Swedish autotetraploid *A. arenosa* (Aa4) accession. Similar to the natural *A. suecica*, *A. thaliana* was the maternal and *A. arenosa* was the paternal plant in this cross. Crosses in the opposite direction were unsuccessful. We managed to obtain very few F_1_ hybrid plants, which after one round of selfing set higher levels of seed. The resulting synthetic line was able to self-fertilize. F_2_ seeds were descended from a common F_1_ and were similar to natural *A. suecica* in appearance. We further continued the synthetic line to F_3_ (selfed third generation).

### Synteny analysis

We performed an all-against-all BLASTP search using CDS sequences for the reference *A. suecica* genome and the ancestral genomes, *A. thaliana* and *A. lyrata*. We used the SynMap tool^151^ from the online CoGe portal^152^. We examined synteny using the default parameters for DAGChainer (maximum distance between two matches = 20 genes; minimum number of aligned pairs = 5 genes).

### Estimating the copy number of rDNA repeats using short DNA reads

To measure the copy number of 45S rRNA repeats in our populations of different species, we aligned short DNA reads to a single reference 45S consensus sequence of *A. thaliana*^153^. An *A. arenosa* 45S rRNA consensus sequence was constructed by finding the best hit using BLAST in our draft *A. arenosa* contig assembly. This hit matched position 1571–8232 bp of the *A. thaliana* consensus sequence, was 6,647 bp in length and is 97% identical to the *A. thaliana* 45S rRNA consensus sequence. The aligned regions of these two 45S rRNA consensus sequences, determined by BLAST, were used in copy number estimates, to ensure that the sizes of the sequences were equal. The relative increase in sequence coverage of these loci, when compared to the mean coverage for the reference genome, was used to estimate copy number.

### Plant material for RNA-seq

Transcriptomic data generated in this study included 15 accessions of *A. suecica*, 16 accessions of *A. thaliana*, 4 accessions of *A. arenosa* and 2 generations of an artificial *A. suecica* line (the second and third selfed generation). The sibling of a paternal *A. arenosa* parent (Aa4) and the maternal tetraploid *A. thaliana* parent (6978, or Wa-1) of our artificial *A. suecica* line were included as part of our samples (Supplementary Data 1). Each accession was replicated three times. Seeds were stratified in the dark for 4 days at 4 °C in 1 ml of sterilized water. Seeds were then transferred to pots in a controlled growth chamber at 21 °C. Humidity was kept constant at 60%. Pots were thinned to two to three seedlings after one week. Pots were re-randomized each week in their trays. Whole rosettes were collected when plants reached the seven-to-nine true-leaf stage of development. Samples were collected between 14:00 and 17:00 and flash-frozen in liquid nitrogen.

### RNA extraction and library preparation

For each accession, two to three whole rosettes in each pot were pooled and total RNA was extracted using the ZR Plant RNA MiniPrep kit. We treated the samples with DNAse and performed purification of mRNA and polyA selection using the AMPure XP magnetic beads and the Poly(A) RNA Selection Kit from Lexogen. RNA quality and degradation were assessed using the RNA Fragment Analyzer (DNF-471 stranded sensitivity RNA analysis kit, 15 nt). The concentration of RNA per sample was measured using the Qubit fluorometer. Library preparation was carried out following the NEBNext Ultra II RNA Library Prep Kit for Illumina. Barcoded adaptors were ligated using NEBNext Multiplex Oligos for Illumina (Index Primers Set 1 and 2). The libraries were PCR-amplified for seven cycles, and 125-bp paired-end sequencing was carried out at the VBCF on Illumina (HiSeq 2500) using multiplexing.

### RNA-seq mapping and gene expression analysis

We mapped 125-bp paired-end reads to the de-novo-assembled *A. suecica* reference using STAR^154^ (v.2.7), and we filtered for primary and uniquely aligned reads using the parameters–outfilterMultimapNmax 1–outSamprimaryFlag OneBestScore. We quantified reads mapped to genes using–quantMode GeneCounts.

To reduce signals that are the result of cross-mapping between the subgenomes of *A. suecica* we used *A. thaliana* and *A. arenosa* as a control. For each gene in the *A. thaliana* subgenome we compared the log fold change of gene counts in our *A. thaliana* population to those in our *A. arenosa* population. We filtered for genes with a log_2_(*A. thaliana*/*A. arenosa*) below 0. We applied the same filters for genes on the *A. arenosa* subgenome. This reduced the number of genes analysed from 22,383 to 21,737 on the *A. thaliana* subgenome, and from 23,353 to 23,221 on the *A. arenosa* subgenome.

Expression analysis was then further restricted to 1:1 unique homeologous gene pairs between the subgenomes of *A. suecica* (17,881 gene pairs). Gene counts were normalized for gene size by calculating the TPM. The effective library sizes were calculated by computing a scaling factor based on the trimmed mean of M-values (TMM) in edgeR^155^, separately for each subgenome. Genes expressed at a low level were removed from the analysis by keeping genes that were expressed in at least three individuals of *A. thaliana* and *A. suecica*, at least one individual of *A. arenosa* and at least one individual of synthetic *A. suecica*. A total of 14,041 homeologous gene pairs satisfied our expression criteria. As *A. suecica* is expressing both subgenomes, to correctly normalize the effective library size in *A. suecica* accessions, the effective library size was calculated as a mean of TPM counts for both subgenomes. The effective library size of *A. thaliana* accessions was calculated for TPM counts using the *A. thaliana* subgenome of the reference genome, as genes from this subgenome will be expressed in *A. thaliana*, and the effective library size of *A. arenosa* lines was calculated using the *A. arenosa* subgenome of the reference *A. suecica* genome. Gene counts were transformed to counts per million (CPM) with a prior count of 1 and were log_2_-transformed. We used the mean of replicates per accession for downstream analyses.

To compare homeologous genes between the subgenomes in *A. suecica* we computed a log fold change using log_2_(*A. arenosa* homeologue/*A. thaliana* homeologue). For tissue-specific genes we took genes that showed a log fold change ≥ 2 in expression between two tissues.

For comparing homologous genes between the (sub-)genomes of *A. suecica* and the ancestral species *A. thaliana* and *A. arenosa*, we performed a Wilcoxon test independently for each of the 14,041 homeologous gene pairs. Using the normalized CPM values, we compared the relative expression level of a gene on the *A. thaliana* subgenome between our populations of *A. thaliana* and *A. suecica*. We performed the same test on the *A. arenosa* subgenome comparing the relative expression of a gene between our populations of *A. arenosa* and *A. suecica*. We filtered for genes with an adjusted *P* value of less than 0.05 (using false discovery rate (FDR) correction). This amounted to 4,186 and 4,571 DEGs for the *A. thaliana* and the *A. arenosa* subgenomes, respectively.

### Cross-mapping of short reads

Cross-mapping of short RNA reads between the subgenomes of *A. suecica* was measured by mixing the RNA reads between *A. thaliana* and *A. arenosa* individuals to generate ‘in-silico’ *A. suecica* individuals. We mapped reads from 10 in-silico *A. suecica* individuals to the *A. suecica* genome. We compared different RNA-seq pipelines to determine cross-mapping error rates. We mapped reads using STAR^154^ (v.2.7), HISAT2^156^ (v.2.1) and EAGLE^157^. Around 1% of *A. thaliana* reads map to the *A. arenosa* subgenome and around 6% of the *A. arenosa* reads map to the *A. thaliana* subgenome, regardless of mapping strategy or pipeline (see Extended Data Fig. 6).

### Expression analysis of rRNA

RNA reads were mapped in a similar manner to DNA reads for the analysis of rDNA copy number. Expression analysis was performed in a similar manner to protein-coding genes, in edgeR. We defined the exclusive expression of a particular 45S rRNA gene by taking a cut-off of 15 for log_2_(CPM) as this was the maximum level of cross-mapping we observed for the ancestral species (see Extended Data Fig. 3).

### Expression analysis of transposable elements

To analyse the expression of transposable elements between species, the annotated TE consensus sequences in *A. suecica* were aligned using BLAST all vs all. Highly similar TE sequences (more than 85% similar for more than 85% of the TE sequence length), were removed, leaving 813 TE families out of 1,213. Filtered *A. suecica* TEs were aligned to annotated *A. thaliana* (TAIR10) and *A. arenosa* (the PacBio contig assembly presented in this study) TE sequences to assign each family to an ancestral species using BLAST. A total of 208 TE families were assigned to the *A. thaliana* parent and 171 TE families were assigned to the *A. arenosa* parent.

RNA reads were mapped to TE sequences using a similar approach as for gene expression analysis using edgeR. TEs that showed expression using a cut-off of log_2_(CPM) > 2 were kept. A total of 121 *A. thaliana* TE sequences and 93 *A. arenosa* TE sequences passed this threshold. We took the mean of replicates per accession for further downstream analyses.

### GO enrichment analysis

We used the R package TopGO^158^ to conduct GO enrichment analysis. We used the ‘weight01’ algorithm when running TopGO, which accounts for the hierarchical structure of GO terms and thus implicitly corrects for multiple testing. GO annotations were based on the *A. thaliana* orthologue of *A. suecica* genes. Gene annotations for *A. thaliana* were obtained using the R package biomaRt^159^ from Ensembl ‘biomaRt::useMart(biomart = ’plants_mart’, dataset = ’athaliana_eg_gene’, host = 'plants.ensembl.org').

### Genome size measurements

We measured genome size for the reference *A. suecica* accession ASS3 and the *A. arenosa* accession used for PacBio (Aa4), using *Solanum lycopersicum* cv. Stupicke (2C = 1.96 pg DNA) as the standard. The reference *A. lyrata* accession MN47 and the *A. thaliana* accession CVI were used as additional controls. Each sample had two replicates.

In brief, the leaves from three-week-old fresh tissue were chopped using a razor blade in 500 µl of UV Precise P extraction buffer with 10 µl mercaptoethanol per ml (kit PARTEC CyStain PI Absolute P no. 05- 5022) to isolate nuclei. Instead of the Partec UV Precise P staining buffer, however, 1 ml of a 5 mg DAPI solution was used, as DAPI provides DNA content histograms with high resolution. The suspension was then passed through a 30-µm filter (Partec CellTrics no. 04-0042-2316) and incubated for 15 minutes on ice before FACS.

Genome size was measured using flow cytometry and a FACS Aria III sorter with near UV 375-nm laser for DAPI. Debris was excluded by selecting peaks when plotting DAPI-W against DAPI-A for 20,000 events.

The data were analysed using the flowCore^160^ package in R. Genome size was estimated by comparing the mean G1 of the standard *Solanum lycopersicum* to that of each sample to calculate the 2C DNA content of that sample using the equation:

$$\begin{array}{l}{\rm{Sample}}\,{\rm{2C}}\,{\rm{DNA}}\,{\rm{content}} = [({\rm{sample}}\,{\rm{G1}}\,{\rm{peak}}\,{\rm{mean}})/({v\;\rm{standard}}\,{\rm{G1}}\,{\rm{peak}}\,{\rm{mean}})]\\ \times \ {{\rm{standard}}\,{\rm{2C}}\,{\rm{DNA}}\,{\rm{content}}}\end{array}$$

We also measured genome size for the reference *A. suecica* accession ASS3 using the software jellyfish^161^ and findGSE^162^ using *k*-mers (21mers). The genome size estimated was 312 Mb, compared to the 305 Mb estimated using FACS (see Extended Data Fig. 1).

### Mapping of TE insertions

We used PoPoolationTE2^100^ (v.v1.10.04) to identify TE insertions. The advantage of this TE-calling software to others is that it avoids a reference bias by treating all TEs as de novo insertions. In brief, it works by using discordant read pairs to calculate the location and abundance of a TE in the genome for an accession of interest.

We mapped 100-bp Illumina DNA reads from previous studies^20,76,163^, in addition to our newly generated synthetic *A. suecica* using BWA-MEM^135^ (v.0.7.15) to a repeat-masked version of the *A. suecica* reference genome, concatenated with our annotated repeat sequences (see ‘Gene prediction and annotation of the *A. suecica* genome’), as this is the data format required by PoPoolationTE2. Reads were given an increased penalty of 15 for being unpaired. Reads were de-duplicated using SAMtools^136^ rmdup (v.1.9). The resulting bam files were then provided to PoPoolationTE2 to identify TE insertions in the genome of each of our *A. suecica*, *A. thaliana* and *A. arenosa* accessions. We used a mapping quality of 10 for the read in the discordant read pair mapping to the genome. We used the ‘separate’ mode in the ‘identify TE signatures’ step and a ‘–min-distance −200–max-distance 500’ in the ‘pairupsignatures’ step of the pipeline. TE counts within each accession were merged if they fell within 400 bp of each other and if they mapped to the same TE sequence. All TE counts (that is, the processed TE counts for each accession) were then combined to produce a population-wide count estimate. Population-wide TE insertions were merged if they mapped to the same TE sequence and fell within 400 bp of each other. Coverage of each TE insertion in the population was also calculated for each accession. The final file was a list of TE insertions present in the population and the presence or absence (or ‘NA’ if there was no coverage to support the presence or absence of a TE insertion) in each accession analysed (Supplementary Data 1).

### Assigning ancestry to TE sequences

To examine TE consensus sequences that have mobilized between the subgenomes of *A. suecica*, we first examined which of our TE consensus sequences (*n* = 1,152) have at least the potential to mobilize (that is, have full-length TE copies in the genome of *A. suecica)*. We filtered for TE consensus sequences that had TE copies in the genome of *A. suecica* that are more than 80% similar in identity for more than 80% of the consensus sequence length (*n* = 936). Of these, 188 consensus sequences were private to the *A. thaliana* subgenome, 460 were private to the *A. arenosa* subgenome and 288 TE consensus sequences were present in both subgenomes of *A. suecica*. To determine whether TEs have jumped from the *A. thaliana* subgenome to the *A. arenosa* subgenome and vice versa we next needed to assign ancestry to these 288 TE consensus sequences. To do this we used BLAST to search for these consensus sequences in the ancestral genomes of *A. suecica*, using the TAIR10 *A. thaliana* reference and our *A. arenosa* PacBio contig assembly. Using the same 80%-80% rule we assigned 55 TEs to *A. arenosa* and 15 TEs to *A. thaliana* ancestry.

### Read mapping and SNP calling

To call biallelic SNPs we mapped reads to the *A. suecica* reference genome using the same filtering parameters described in ‘Mapping of TE insertions’. Biallelic SNPs were called using HaplotypeCaller from GATK^164^ (v.3.8) using default quality thresholds. SNPs were annotated using SnpEff^165^. Biallelic SNPs on the *A. thaliana* subgenome were polarized using 38 diploid *A. lyrata* lines^76^ and biallelic SNPS on the *A. arenosa* subgenome were polarized using 30 *A. thaliana* accessions^163^ closely related to *A. suecica*^20^.

### Chromosome preparation and FISH

Whole inflorescences of *A. arenosa*, *A. suecica* and *A. thaliana* were fixed in freshly prepared ethanol:acetic acid fixative (3:1) overnight, transferred into 70% ethanol and stored at −20 °C until use. Selected inflorescences were rinsed in distilled water and citrate buffer (10 mM sodium citrate, pH 4.8), and digested by a 0.3% mix of pectolytic enzymes (cellulase, cytohelicase, pectolyase; all from Sigma-Aldrich) in citrate buffer for around 3 h. Mitotic chromosome spreads were prepared from pistils as previously described^166^ and suitable slides were pretreated by RNase (100 µg ml^−1^, AppliChem) and pepsin (0.1 mg ml^−1^, Sigma-Aldrich).

For identification of *A. thaliana* and *A. arenosa* subgenomes in the allotetraploid genome of *A. suecica*, FISH probes were made from plasmids pARR20–1 or pAaCEN containing 180 bp of *A. thaliana* (pAL) or around 250 bp of *A. arenosa* (pAa) pericentromeric repeats, respectively. The *A. thaliana* BAC clone T15P10 (AF167571) bearing 45S rRNA gene repeats was used for in situ localization of nucleolar organizer regions (NORs). Individual probes were labelled with biotin–dUTP, digoxigenin–dUTP and Cy3–dUTP by nick translation, pooled, precipitated and resuspended in 20 µl of hybridization mixture (50% formamide and 10% dextran sulfate in 2× saline sodium citrate (2× SSC)) per slide as previously described^96^.

Probes and chromosomes were denatured together on a hot plate at 80 °C for 2 min and incubated in a moist chamber at 37 °C overnight. Post-hybridization washing was performed in 20% formamide in 2× SSC at 42 °C. Fluorescent detection was as follows: biotin–dUTP was detected by avidin–Texas Red (Vector Laboratories) and amplified by goat anti-avidin–biotin (Vector Laboratories) and avidin–Texas Red; digoxigenin–dUTP was detected by mouse anti-digoxigenin (Jackson ImmunoResearch) and goat anti-mouse Alexa Fluor 488 (Molecular Probes). Chromosomes were counterstained with DAPI (4’,6-diamidino-2-phenylindole; 2 μg ml^−1^) in Vectashield (Vector Laboratories). Fluorescent signals were analysed and photographed using a Zeiss Axioimager epifluorescence microscope and a CoolCube camera (MetaSystems). Images were acquired separately for the four fluorochromes using appropriate excitation and emission filters (AHF Analysentechnik). The monochromatic images were pseudo-coloured and merged using Adobe Photoshop CS6 software (Adobe Systems).

### DAP-seq enrichment analysis for transcription factor target genes

We downloaded the target genes of transcription factors from the plant cistrome database (http://neomorph.salk.edu/dap_web/pages/index.php), which is a collection of transcription-factor-binding sites and their target genes, in *A. thaliana*, based on DAP-seq^167^. To test for enrichment of a gene set (for example, the genes in *A. thaliana* cluster 2 on Fig. 5) for target genes of a particular transcription factor, we performed a hyper-geometric test in R. As a background we used the total 14,041 genes used in our gene expression analysis. We then performed FDR correction for multiple testing to calculate an accurate *P* value of the enrichment.

### Reporting Summary

Further information on research design is available in the Nature Research Reporting Summary linked to this article.

## Supplementary information

Reporting Summary

Peer Review Information

Supplementary Tables 1 and 2 Supplementary Table 1. GO analysis for gene expression comparison between whole rosettes and floral buds in *A. suecica*. No significant GO was found for genes biased towards the *A. thaliana* subgenome of *A. suecica* for floral buds. Supplementary Table 2. List of overrepresented gene ontologies from Fig. 5e.

Supplementary Data 1 PoPoolationTE2 calls for *A. suecica* and the ancestral species for the subgenomes of *A. suecica.*

Supplementary Data 2 RNA-seq mapping statistics, DEGs, overlap with the CyMIRA and DAP-seq databases and gene evolutionary relationships in *A. suecica.*

Supplementary Data 3 Log fold change and CPM for genes on the *A. thaliana* and *A. arenosa* subgenome of *A. suecica.*

Supplementary Data 4 Gene annotation of the *A. suecica* genome.

Supplementary Data 5 TE sequences in *A. suecica* and TE hierarchy files.

## Data Availability

Genome assemblies and raw short reads can be found in the European Nucleotide Archive (ENA) (https://www.ebi.ac.uk/ena/browser/home). The genome assembly for *A. suecica* ASS3 can be found under the BioProject number PRJEB42198, assembly accession GCA_905175345. The raw reads for the *A. suecica* genome assembly generated by Pacbio RSII can be found under ERR5037702 and those from Sequel under ERR5031296. The Hi-C reads used for scaffolding the *A. suecica* assembly can be found under ERR5032369. The contig assembly for tetraploid *A. arenosa* (ssp. *arenosa*) can be found under the BioProject number PRJEB42276, assembly accession GCA_905175405. The raw reads for the *A. arenosa* Aa4 contig assembly generated by Sequel can be found under ERR5031542 and the reads generated by Nanopore under ERR5031541. Hi-C reads for the *A. arenosa* assembly can be found under ERR5032370. Hi-C sequencing data for the ancestral species, the outlier accession AS530 and synthetic *A. suecica* can be found under the BioProject number PRJEB42290. DNA resequencing data of synthetic *A. suecica* and parents generated in this study can be found under the BioProject number PRJEB42291. The RNA-seq reads are under the BioProject number PRJEB42277. TE presence or absence calls for *A. suecica* and the ancestral species can be found in Supplementary Data 1. A list of DEGs, orthologues, enriched DAP-seq transcription factors, CyMIRA gene overlaps and RNA-seq mapping statistics can be found in Supplementary Data 2. Log fold change and CPM for genes on the *A. thaliana* and *A. arenosa* subgenome can be found in Supplementary Data 3. The gene annotation (gff3 file) of the *A. suecica* genome can be found in Supplementary Data 4. TE consensus sequences and a hierarchy file of TE order for *A. suecica* can be found in Supplementary Data 5.
