# Supplementary material for: Gradual evolution of allopolyploidy in Arabidopsis suecica
Source: Nat Ecol Evol. 2021 Aug 19;5(10):1367–81. doi: 10.1038/s41559-021-01525-w (PMC8484011; doi:10.1038/s41559-021-01525-w)
Supplement: Supplementary file 1 — Reporting Summary [file 41559_2021_1525_MOESM1_ESM.pdf]

nature portfolio

Corresponding author(s): Dr. Polina Yu. Novikova, Dr. Magnus Nordborg

Last updated by author(s): Jun 18, 2021

Reporting Summary

Nature Portfolio wishes to improve the reproducibility of the work that we publish. This form provides structure for consistency and transparency in reporting. For further information on Nature Portfolio policies, see our Editorial Policies and the Editorial Policy Checklist.

Statistics

For all statistical analyses, confirm that the following items are present in the figure legend, table legend, main text, or Methods section.

n/a	Confirmed	
☐	☒	The exact sample size (n) for each experimental group/condition, given as a discrete number and unit of measurement
☐	☒	A statement on whether measurements were taken from distinct samples or whether the same sample was measured repeatedly
☐	☒	The statistical test(s) used AND whether they are one- or two-sided<br>*Only common tests should be described solely by name; describe more complex techniques in the Methods section.*
☒	☐	A description of all covariates tested
☐	☒	A description of any assumptions or corrections, such as tests of normality and adjustment for multiple comparisons
☐	☒	A full description of the statistical parameters including central tendency (e.g. means) or other basic estimates (e.g. regression coefficient) AND variation (e.g. standard deviation) or associated estimates of uncertainty (e.g. confidence intervals)
☒	☐	For null hypothesis testing, the test statistic (e.g. F, t, r) with confidence intervals, effect sizes, degrees of freedom and P value noted<br>*Give P values as exact values whenever suitable.*
☒	☐	For Bayesian analysis, information on the choice of priors and Markov chain Monte Carlo settings
☒	☐	For hierarchical and complex designs, identification of the appropriate level for tests and full reporting of outcomes
☒	☐	Estimates of effect sizes (e.g. Cohen's d, Pearson's r), indicating how they were calculated

Our web collection on statistics for biologists contains articles on many of the points above.

Software and code

Policy information about availability of computer code

Data collection	No software was used
Data analysis	FALCON (version 0.3.0), Arrow (smrtlink release 5.0.0.6792), Pilon (version 1.22), HiCUP (version 0.6.1), MUMmer (version 3.23), LACHESIS (version 1.0.0), BWA-MEM (version 0.7.15), Samtools (version 0.1.19), AUGUSTUS, GenomeThreader (version 1.7.0), RepeatModeler (version 1.0.11), RepeatMasker (version 4.0.7), STAR (version 2.7), HISAT2 (version 2.1), EAGLE, PopoolationTE2 (version v1.10.04), BLAST, R package HMM, R package TopGO, R package EdgeR, R package biomaRt, R package flowCore

For manuscripts utilizing custom algorithms or software that are central to the research but not yet described in published literature, software must be made available to editors and reviewers. We strongly encourage code deposition in a community repository (e.g. GitHub). See the Nature Portfolio guidelines for submitting code & software for further information.

Data

Policy information about availability of data

All manuscripts must include a data availability statement. This statement should provide the following information, where applicable:

- Accession codes, unique identifiers, or web links for publicly available datasets
- A description of any restrictions on data availability
- For clinical datasets or third party data, please ensure that the statement adheres to our policy

Genome assemblies and raw short reads can be found in the European Nucleotide Archive (ENA) (https://www.ebi.ac.uk/ena/browser/home).
The genome assembly for A. suecica ASS3 can be found under the BioProject number PRJEB42198, assembly accession GCA_905175345. The raw reads for the A. suecica genome assembly generated by Pacbio RSII can be found under ERR5037702 and those from Sequel under ERR5031296. The HiC reads used for scaffolding the A. suecica assembly can be found under ERR5032369.

The contig assembly for tetraploid A. arenosa (ssp. arenosa) can be found under the BioProject number PRJEB42276, assembly accession GCA_905175405. The raw reads for the A. arenosa Aa4 contig assembly generated by Sequel can be found under ERR5031542 and the reads generated by Nanopore under ERR5031541. HiC reads for the A. arenosa assembly can be found under ERR5032370.
HiC sequencing data for the ancestral species, the outlier accession AS530 and synthetic A. suecica can be found under the BioProject PRJEB42290.
DNA resequencing of synthetic A. suecica and parents generated in this study can be found under the BioProject PRJEB42291.
The RNA-seq reads are under the BioProject number PRJEB42277.
TE presence/absence calls for A. suecica and the ancestral species can be found in Supplementary Data 1.
A list of DEGs, orthologs, enriched DAP-seq transcription factors, CyMIRA gene overlaps and RNA-seq mapping statistics can be found in Supplementary Data 2.
Log fold change and CPM (counts per million) for genes on the A. thaliana and A. arenosa subgenome can be found in Supplementary Data 3.
The gene annotation (gff3 file) of the A. suecica genome can be found in Supplementary Data 4.
TE consensus sequences and a hierarchy file of TE order for A. suecica can be found in Supplementary Data 5.

Field-specific reporting

Please select the one below that is the best fit for your research. If you are not sure, read the appropriate sections before making your selection.

☒ Life sciences ☐ Behavioural & social sciences ☐ Ecological, evolutionary & environmental sciences

For a reference copy of the document with all sections, see nature.com/documents/nr-reporting-summary-flat.pdf

Life sciences study design

All studies must disclose on these points even when the disclosure is negative.

Sample size	No sample size calculation was preformed
Data exclusions	No data was excluded from the analysis
Replication	Attempts at replication were successful
Randomization	Samples were allocated to species groups
Blinding	Not relevant to study

Reporting for specific materials, systems and methods

We require information from authors about some types of materials, experimental systems and methods used in many studies. Here, indicate whether each material, system or method listed is relevant to your study. If you are not sure if a list item applies to your research, read the appropriate section before selecting a response.

Materials & experimental systems

n/a	Involved in the study	
☒	☐	Antibodies
☒	☐	Eukaryotic cell lines
☒	☐	Palaeontology and archaeology
☒	☐	Animals and other organisms
☒	☐	Human research participants
☒	☐	Clinical data
☒	☐	Dual use research of concern

Methods

n/a	Involved in the study	
☒	☐	ChIP-seq
☒	☐	Flow cytometry
☒	☐	MRI-based neuroimaging
