# Supplementary material for: Gradual evolution of allopolyploidy in Arabidopsis suecica
Source: Nat Ecol Evol. 2021 Aug 19;5(10):1367–81. doi: 10.1038/s41559-021-01525-w (PMC8484011; doi:10.1038/s41559-021-01525-w)
Supplement: Supplementary file 2 — Peer Review Information [file 41559_2021_1525_MOESM2_ESM.pdf]

Peer Review Information

Journal: Nature Ecology & Evolution
Manuscript Title: Gradual evolution of allopolyploidy in *Arabidopsis suecica*
Corresponding author name(s): Polina Yu. Novikova, Magnus Nordborg

Editorial Notes:

Reviewer Comments & Decisions:

Decision Letter, initial version:

21st October 2020

*Please ensure you delete the link to your author homepage in this e-mail if you wish to forward it to your co-authors.

Dear Magnus,

Your manuscript entitled "Gradual evolution of allopolyploidy in <i>Arabidopsis suecica</i>" has now been seen by three reviewers, whose comments are attached. The reviewers have raised a number of concerns which will need to be addressed before we can offer publication in Nature Ecology & Evolution. We will therefore need to see your responses to the criticisms raised and to some editorial concerns, along with a revised manuscript, before we can reach a final decision regarding publication.

We therefore invite you to revise your manuscript taking into account all reviewer and editor comments. Please highlight all changes in the manuscript text file in Microsoft Word format.

We are committed to providing a fair and constructive peer-review process. Do not hesitate to contact us if there are specific requests from the reviewers that you believe are technically impossible or unlikely to yield a meaningful outcome.

When revising your manuscript:

* Include a “Response to reviewers” document detailing, point-by-point, how you addressed each reviewer comment. If no action was taken to address a point, you must provide a compelling argument. This response will be sent back to the reviewers along with the revised manuscript.

* If you have not done so already please begin to revise your manuscript so that it conforms to our Article format instructions at http://www.nature.com/natecolevol/info/final-submission. Refer also to any guidelines provided in this letter.

* Include a revised version of any required reporting checklist. It will be available to referees (and, potentially, statisticians) to aid in their evaluation if the manuscript goes back for peer review. A revised checklist is essential for re-review of the paper.

Please use the link below to submit your revised manuscript and related files:

[REDACTED]

Note: This URL links to your confidential home page and associated information about manuscripts you may have submitted, or that you are reviewing for us. If you wish to forward this email to co-authors, please delete the link to your homepage.

We hope to receive your revised manuscript within four to eight weeks. If you cannot send it within this time, please let us know. We will be happy to consider your revision so long as nothing similar has been accepted for publication at Nature Ecology & Evolution or published elsewhere.

Nature Ecology & Evolution is committed to improving transparency in authorship. As part of our efforts in this direction, we are now requesting that all authors identified as 'corresponding author' on published papers create and link their Open Researcher and Contributor Identifier (ORCID) with their account on the Manuscript Tracking System (MTS), prior to acceptance. ORCID helps the scientific community achieve unambiguous attribution of all scholarly contributions. You can create and link your ORCID from the home page of the MTS by clicking on 'Modify my Springer Nature account'. For more information please visit please visit www.springernature.com/orcid.

Please do not hesitate to contact me if you have any questions or would like to discuss these revisions further.

We look forward to seeing the revised manuscript and thank you for the opportunity to review your work.

[REDACTED]

Reviewer expertise:

Reviewer #1: evolution of polyploidy in Arabidopsis, de nove genome sequencing

Reviewer #2: evolution of polyploidy in Arabidopsis

Reviewer #3: genetic mechanisms of polyploidy and crop domestication

Reviewers' comments:

Reviewer #1 (Remarks to the Author):

The authors focused on the allopolyploid Arabidopsis suecica to study the genomic and transcriptomic effect of allopolyploidization. Since around 2000, many studies reported a large scale and stochastic genomic, epigenetic and transcriptional changes following polyploidization, termed "genome shock" (originally coined by McClintock in a different context). Here the authors examined whether such changes were observed. They conducted a diverse array of analysis including genome assembly, synteny, rDNA clusters, transposable elements in population samples, test of expression bias between subgenomes, and the comparison of expression between diploid and polyploid species. After all, they did not find "genome shock" (the definition should be more explicit, though). I do not think this is a negative result, but provide a solid data to understand the evolution. Many of the results are novel. Section 3 of transposable elements using population samples is particularly interesting. The chromosome level substitutions in the section 6 was identified thanks to "unparalleled in its resolution thanks to one of the parents being a major model species" (line 479). I would suggest the manuscript can become suitable for Nature Ecology and Evolution.
However, there are major issues to be revised. In the abstract, introduction and discussions, the central theme of the authors is:
genome shock was observed in crop polyploid species, but natural species are not well studied in this aspect (e.g., lines 20, 54, 492)
This dichotomy of crop and natural species is based on a shallow coverage of previous literatures by the authors. This question needs to be refocused or different questions should be addressed. Genome shock was fashionable about 20 years ago, but most of later studies did not support it possibly because of species difference or of improved bioinformatic analysis. Unfortunately, there is a bias in the references because studies showing genome shock tend to receive attentions in high rank journals while those without genome shock may be considered negative results. Due to this reason, the genome shock may not be mentioned in the title or in abstract, and thus one may need to read the text. Below I will mention some of representative cases below.

In Abstract, Line 24 said "It is unclear, however, whether domesticated polyploids are representative of natural ones." However, using natural species, there are both types of studies with or without genome shock. For example, on the one hand, many papers on natural contemporary polyploid Tragopogon discussed transcriptomic shock and gene loss (for example, Transcriptomic shock generates evolutionary novelty in a newly formed, natural allopolyploid plant. Buggs et al. Curr Biol. 2011 Apr 12;21(7):551-6); Gene loss and silencing in Tragopogon miscellus (Asteraceae): comparison of natural and synthetic allotetraploids. Buggs et al. Heredity (Edinb). 2009 Jul;103(1):73-81.). There is a relatively recent paper on Mimulus (Subgenome Dominance in an Interspecific Hybrid, Synthetic Allopolyploid, and a 140-Year-Old Naturally Established Neo-Allopolyploid Monkeyflower. Edger et al., Plant Cell 2017 Sep;29(9):2150-2167.)
On the other hand, many natural species did not show obvious genome shock. Although the authors cited a few papers, most relevant for this manuscript would be the allopolyploid Arabidopsis kamchatica, which belongs to the same genus as the focal species A. suecica. For example, the title of the following paper explicitly mentioned the lack of stochastic changes in transcriptomes (Genome-wide quantification of homeolog expression ratio revealed nonstochastic gene regulation in synthetic allopolyploid Arabidopsis. Akama et al. Nucleic Acids Res. 2014 Apr;42(6):e46). Loss-of-function mutations were rarely fixed, suggesting that gene loss by genomic shock was not pervasive (Patterns of polymorphism and selection in the subgenomes of the allopolyploid Arabidopsis kamchatica. Paape

et al. Nat Commun. 2018 Sep 25;9(1):3909). The authors did cite a recent paper using Brachypodium in a different sentence, but it should be discussed in this context (Gradual polyploid genome evolution revealed by pan-genomic analysis of Brachypodium hybridum and its diploid progenitors. Gordon et al. Nat Commun. 2020 Jul 29;11(1):3670).

In many crop species, genome shock was not drastic or observed, although the authors did not say explicitly all/most crop species (some sentences implicitly imply). In wheat community, I would say it is a common sense that genome shock is not relevant for wheat. Although studies around 2000 suggested genome shock (e.g., Ozkan e t al. Plant Cell 13; 1735, 2001; Shaked et al. Plant Cell 13: 1749, 2001), genome-wide analysis with improved bioinformatics did not show clear evidence. A recent high-quality genome assembly paper by the International Wheat Genome Sequencing Consortium (Science 361: 661, 2018) said that "As reported (18), no evidence was found for a major burst of transposition after polyploidization. The independent evolution in the diploid lineages was reflected in differences in the specific composition of the A, B, and D subgenomes at the subfamily (variants) level". (note that this confirmed a previous Science paper in 2014, its reference 18). There are literatures with or without genome shock in crop Brassica species. I hope the authors would find such literatures further.

The current manuscript neglecting these and other relevant references is not suitable for any reasonable journal. The authors should cite these references and refocus the title, abstract and conclusions. Line 54 "However, genomic changes resulting from the hybridization of two (or more) diverged genomes have primarily been studied in crops, such as cotton5, strawberry6, wheat7 and peanut8,9, which confounds polyploidization with domestication." is clearly wrong, see references above. Yet, I strongly suggest that the data and analysis are novel and more than adequate for Nature Ecology and Evolution when rewritten.

There is a major methodological concern on transcriptomic analysis in the section 4. In the line 965 of the Material and Methods, the authors mapped the reads directly to the allopolyploid genome using STAR as if it is a diploid species. Recent bioinformatic development showed that such analysis can result in high rate of mapping errors ("cross mapping between the subgenomes" in line 968). When STAR is used, the error rate was roughly 10% in wheat and Arabidopsis (Homeolog expression quantification methods for allopolyploids. Kuo et al. Brief Bioinform. 2020 Mar 23;21(2):395-407), which would affect the estimation of subgenome bias. The effect of the bioinformatic tools on the subgenome bias was also shown recently (Homoeologous gene expression and co-expression network analyses and evolutionary inference in allopolyploids. Hu et al. Brief Bioinform. Online ahead of print 10.1093/bib/bbaa035). For A. suecica, the error rate is probably less, seeing "the substantial (~11,6%) divergence between the subgenomes" (line 84), but the bias may matter for conserved genes (histone genes of two subgenomes may be highly similar). These literatures indicate several bioinformatic workflows of subgenome-classification approaches for allopolyploid species such as EAGLE-RC, HomeoRoq, GSNAP-PolyCat. The current result should be verified by using such methods.

In the section 5, the evidence for "gradual evolution" is not strong although it is in the manuscript title. In the line 350, the authors discussed "For both subgenomes, the first principal component separates A. suecica from the ancestral species and the synthetic hybrid (Fig. 5a,b), suggesting that hybridization does not automatically result in large-scale transcriptional changes, and that altered gene expression changes in natural A. suecica have evolved over time." However, a simple other possibility is that the very diploid individual contributed to the natural allopolyploidization was different from the examined samples, and may have come close to natural hybrid in the PCA plot. Unless obtaining ancient DNA, this is very difficult to test. Other than this analysis, there does not seem direct/strong evidences of "gradual" evolution. The authors should present other evidences, or change the title as well as the abstract and the conclusion (e.g., line 481).

Abstract
Line 38 as well as Line 414. There is a big logical jump between the enrichment of plastid genes and nucleo-cytoplasmic compatibility. I do not think this possibility can be discussed without experimental evidence.

Introduction
Line 67, a space is missing after a period

Results and discussion
Line 82. "naturally inbred A. suecica" is confusing. Do you mean that it selfed in natural populations? If so, show some evidence of inbred status. I imagine the authors would like to mean that A. suecica is a natural species and selfing was conducted in the lab. The same issue in line 71.

Fig. 1a. LGM in the middle of tree is ambiguous. If 6-8 mya is shown, time range may be shown, too, because time estimates must be relative depending on calibration points.

Section 3
The text says Figure 4 repeatedly but it must be Figure 3.

The authors discussed the potential reasons of the difference in TE activities of two subgenomes. In literatures, there are several studies showing reduced number, activity or selection of TEs in A. thaliana, which may be associated with the transition to self-fertilization (e.g., The evolution of transposable elements in natural populations of self-fertilizing Arabidopsis thaliana and its outcrossing relative Arabidopsis lyrata. Lockton S, Gaut BS. BMC Evol Biol. 2010 Jan 12;10:10; The predominantly selfing plant Arabidopsis thaliana experienced a recent reduction in transposable element abundance compared to its outcrossing relative Arabidopsis lyrata. de la Chaux et al. Mob DNA. 2012 Feb 7;3(1):2). Although A. lyrata were used in these studies, the authors used A. lyrata as the closest substitute for A. arenosa, and the patterns would be similar. The discussion should include these aspects.

Lines 214 and 222. "See below" was used repeatedly, but it is far in the text, and the relevance is not explicitly explained below.

Line 230. "the distribution is more similar to that of non-synonymous SNPs than to that of synonymous SNPs (Fig 4b and c)". Seeing the figure, the frequency spectrum of TEs are very different from either non synonymous or synonymous sites (for example, at 0, non-syn 0.1, syn 0.09, TEs 0.24) and thus the sentence is confusing even if it may not be incorrect.

Line 381-2. The text does not tell the connection between nitrate and Fennoscandinavia.

Line 392. Grammatically strange: A. suecica A. thaliana

Line 400, it is too strong to say "must reflect trans-regulation". There may be other complicated scenarios.

Line 420. The finding of homeologous exchange is interesting, and this is thanks to the high quality analysis using the model species. It is important to define "genome shock" clearly if the authors decided to continue using it. Some researchers may consider that the changes observed here is a part

of genome shock.

Supplementary Figures
Line 547, Erecta should be erecta
Line 548, typo, that of and

Materials and Methods
line 791, "We extracted 64g of whole genomic DNA". The amount seems too large, and likely a mistake. As in line 767, DNA may be extracted from tens of grams of tissues.

Reviewer #2 (Remarks to the Author):

This is an interesting and useful paper describing the genome of allopolyploid Arabidopsis suecica. What is especially powerful here, is that the authors provide a comparison not only of the two sub-genomes within A. suecica, but also (1) an assessment of diversity within A. suecica extant accessions and (2) a comparison of both established and newly generated A. suecica to the parents. This is one of only few attempts to sequence the genome of a non-crop allopolyploid, and the only one I know of to this quality and with the comparison to two extant and well characterised parents, which gives this study quite a bit of power.

I like it, I think it is an important contribution also because it dispels some things that are assumed to be generalities about allopolyploids. The authors (1) generated a high quality genome for A. suecica, (2) showed that A. suecica does not have an extensive "genome shock" history, but they do show evidence of three separate homologous exchange events, (3) showed there is no sub genome dominance either in rRNA loci, or in gene expression, (4) showed the dominance does not vary by tissue as it does in some other species, (5) show there was no abrupt change in gene expression or massive transposon activation and (6) show there was longer term transcriptional adjustment, perhaps adaptive, in established suecica. To me the result that there is variation for the extent of nucleolar dominance was especially cool...

I have only some minor points for clarification - and one suggestion for an analysis.

1) Could you please provide lists of the actual DE genes?

2) It would be cool to see a PCA that includes the arenosa sub genome of suecica together with extant diploid and tetraploid A. arenosa to see if the ancestor might have been diploid or tetraploid (as this matters for the conclusion that perhaps the thaliana genome did more meiotic adaptation in suecica). I think this analysis would add to the story, though I don't see it as essential.

3) Line 137 - it is not entirely clear what is meant by "lineage-specific genes in A. suecica"

4) Lines 316-317: this is also true in cotton, should probably cite that - see: Adams et al 2014, Genetics 168:2217-2226.

Reviewer #3 (Remarks to the Author):

In this study, Burns et al. sequenced natural A. suecica genomes and compared genomic and gene expression changes in the allotetraploids with those of putative progenitors, A. thaliana (At) and A. arenosa (Aa). They concluded that the evolutionary changes in the two subgenomes are gradual with no obvious dominance of subgenomic variation or TE activities but down-played several thousands of differentially expressed genes (DEGs) identified between the At and Aa subgenomes. Moreover, they show upregulation of meiosis-related genes and the genes in the cytoplasmic-nuclear related process. The sequence data of A. suecica is of high quality and will be useful resources for the plant genomics and evolution communities. The quality of A. arenosa sequence is poor and should be improved in the future. It appears that the authors have attempted to emphasise the gradual evolution of the genomes, by mis-interpreting some data with biased views and speculative statements, ignoring many previous reports pertinent to the topic.

A major concern is over-interpretation or misinterpretation of their results. Although A. suecica is a naturally formed allotetraploids, itself alone cannot represent a wide range of natural occurring polyploids. In contrast to their claims that genomic reshuffling or dominance occurs only in polyploid crops, it also occurs in Tragopogon miscellus (Chester et al. 2012, PNAS), an allotetraploid formed in natural ecological environments in ~150 years. The authors have mis-interpreted two sets of important factual data in polyploid literature. First, the perceived "domestication" effect has little or nothing to do with evolutionary and biological consequences on polyploids. There are two kinds of allopolyploids with respect to genomic stability and exchanges. One kind, like A. suecica and cotton allotetraploids, does not show rapid genomic reshuffling; the other type, like synthetic Brassica napus, and newly formed T. miscellus in natural ecological conditions, displays rapid genomic reshuffling. Second, the age of polyploids has little or no effect on genomic exchanges. For example, A. suecica is estimated to form 16,000-300,000 years ago, and allotetraploid cotton was formed ~1.5 million years ago, while hexaploid wheat was formed 8,000-10,000 years, so was B. napus (Lu et al. 2019 Nature Communications). Those natural polyploids formed at different evolutionary time (from 16,000 years to 1.5 million years) plus newly formed allotetraploid cotton (Liu et al. 2001, Genome) are all genetically stable. However, resynthesised B. napus (Xiong et et al. 2011, PNAS) and wheat (Feldman et al. 1997, Genetics) show rapid genomic exchanges. This is probably because the exact progenitors that formed those "stable" polyploids became extinct over time. The extant species available today are not the same progenitors as they were 8,000-10,000 years ago. These misconceptions about the effects of "age" and "domestication" on genomic stability in polyploids should be completely revised to reflect a plethora of data documented in the literature. Along the same line, the quote from Charles Darwin (line 493), albeit elegant, does not support their speculation on polyploidy and should be removed.

Comments on data and technical validity

1. Lines 88-89, "Our attempt to assemble a tetraploid A. arenosa, which is also included here, led to a very fragmented assembly of 3,629 contigs with an N50 of 331 Kb." This quality score of A. arenosa genome is poor. As a result, they should tone down the statements on comparative data with A. arenosa, especially in the area of structural variations, genomic synteny, and TE activities. This should also apply to syntenic relationships between A. arenosa and A. thaliana or A. lyrata (Figure 1b, d).

2. rDNA dominance. This part of data is confusing or misleading. Data in Fig. 2a did not show a clear pattern of A. arenosa rDNA expression dominance over A. thaliana rDNA, in a phenomenon known as

nucleolar dominance, as cited in Pontes et al. (2004, PNAS), but many other reports were not cited. Instead, they seem to co-express, which is not supported by numerous other reports. It is also odd that they noted loss of the majority of A. thaliana rDNA copies in AS90a line. Based on the FISH images in Fig. 2d, 2e, the large staining area of rDNA clusters (from A. arenosa?) disappeared, compared to data in Fig. 2b, 2c. The small clusters in Fig. 2b-e should be A. thaliana (instead of A. arenosa), based on data presentation in Fib. 2b, 2c. It is misleading if the probe cross-hybridised both A. thaliana and A. arenosa rDNA, how can the Aa and At NORs be discriminated, as the centromere signals were not clearly indicative?

3. When describing TE specificity, they should use "specific" or "unique", and TEs should be specific (instead of "private") to A. thaliana or A. arenosa, etc. Because the TE annotation in A. arenosa could be over- or under-estimated due to poor genome assemblies, the difference between Aa and At subgenomes could be more than just a "bottleneck." Although the analysis is justified, the conclusion has exaggerated the similarities and ignored the differences. What is the difference between "A. arenosa in A. suecica" and "A. arenosa in A. suecica (4n) in Fig. 3a?

4. Expression bias or dominance (Fig. 4). They referenced A. thaliana, Zea mays, B. rapa, and G. raimondii (lines 282-283), all of which were "paleopolyploids" or ancient autopolyploids (though exact origin in some like Z. mays is unknown). These are very different from A. suecica, an allotetraploid. They should appropriately reference results from many other allopolyploids. A simple Google search would give them proper references needed.

4. The expression results suggest that overall the two subgenomes are expressed at similar levels, this does not preclude that some genes can exhibit expression bias between homoeologs. Indeed, they identified some homoeologs that show expression bias in Supplemental Fig. 14b, c, especially in resynthesised A. suecica. The other possibility is that heat maps and violin plots in separate subgenomes cannot identify those expression bias of individual homoeolog between the subgenomes. For example, if one set of 1,000 At genes were overexpressed, while another set of 1,000 Aa genes was upregulated, the overall plots would be similar. Indeed, the conclusion of no expression bias in this section is inconsistent with the expression evolution (3,800-4,100 DEGs in Aa or At subgenomes, a total of ~8,000 DEGs!!!) among different A. suecica lines (see below). Alternative methods such as using nonadditive expression values (different from the average of the two parents), especially in synthetic A. suecica, would help resolve this issue.

5. Expression evolution in A. suecica. The PCA plots clearly showed expression divergence between A. thaliana and A. arenosa in these analyses (Fig. 5). "The total number of DEGs was 3,854 and 4,136 genes for the A. thaliana and A. arenosa subgenomes, respectively (see Methods)." (Lines 373-374). Notably, these numbers of DEGs are similar to several previous reports in this polyploid species, which the authors did not cite. This along with a major concern and others about misinterpretation of their data, leading to their biased perception of "no difference." While A. suecica subgenomes are stable and colinear as they are, like other stable polyploids, the differences, especially in gene expression and phenotypic traits between A. suecica and A. thaliana or A. arenosa, are there and should be reported and discussed.

In summary, the genomic sequences of A. suecica have been improved from their previous work (Novikova et al. 2017 MBE) and should provide a good resource for the plant genomics and evolutionary biology communities. The quality of A. areonosa sequence is poor and should be improved. Throughout the manuscript, the data are over- or mis-interpreted with a one-sided (biased)

view of polyploid evolution, which is also reflected in their reference citations. The quote from C. Darwin (line 493) does not support their scope of study, nor is consistent with a plethora of data available in the literature and should be removed.

*******************END*******************

Author Rebuttal to Initial comments

Response to reviewers

We thank the Reviewers for their feedback, and note that, in addition to responding to the comments below, we took the opportunity to update our results using an improved genome. This resulted in minor changes throughout, although no conclusions were affected.

Reviewer #1

The authors focused on the allopolyploid Arabidopsis suecica to study the genomic and transcriptomic effect of allopolyploidization. Since around 2000, many studies reported a large scale and stochastic genomic, epigenetic and transcriptional changes following polyploidization, termed "genome shock" (originally coined by McClintock in a different context). Here the authors examined whether such changes were observed. They conducted a diverse array of analysis including genome assembly, synteny, rDNA clusters, transposable elements in population samples, test of expression bias between subgenomes, and the comparison of expression between diploid and polyploid species. After all, they did not find "genome shock" (the definition should be more explicit, though). I do not think this is a negative result, but provide a solid data to understand the evolution. Many of the results are novel. Section 3 of transposable elements using population samples is particularly interesting. The chromosome level substitutions in the section 6 was identified thanks to "unparalleled in its resolution thanks to one of the parents being a major model species" (line 479). I would suggest the manuscript can become suitable for Nature Ecology and Evolution.

We thank the Reviewer for these encouraging remarks and respond to the issues alluded to in what follows.

However, there are major issues to be revised. In the abstract, introduction and discussions, the central theme of the authors is:
genome shock was observed in crop polyploid species, but natural species are not well studied in this aspect (e.g., lines 20, 54, 492)
This dichotomy of crop and natural species is based on a shallow coverage of previous literatures by the authors. This question needs to be refocused or different questions should be addressed. Genome shock was fashionable about 20 years ago, but most of later studies did not support it possibly because of species difference or of improved bioinformatic analysis. Unfortunately, there is a bias in the references because studies showing genome shock tend to receive attentions in high rank journals while those without genome shock may be considered negative results. Due to this reason, the genome shock may not be mentioned in the title or in abstract, and thus one may need to read the text. Below I will mention some of representative cases below.

In Abstract, Line 24 said "It is unclear, however, whether domesticated polyploids are representative of natural ones." However, using natural species, there are both types of studies with or without genome shock. For example, on the one hand, many papers on natural contemporary polyploid **Tragopogon** discussed transcriptomic shock and gene loss (for example, Transcriptomic shock generates evolutionary novelty in a newly formed, natural

allopolyploid plant. Buggs et al. Curr Biol. 2011 Apr 12;21(7):551-6); Gene loss and silencing in Tragopogon miscellus (Asteraceae): comparison of natural and synthetic allotetraploids. Buggs et al. Heredity (Edinb). 2009 Jul;103(1):73-81.). There is a relatively recent paper on Mimulus (Subgenome Dominance in an Interspecific Hybrid, Synthetic Allopolyploid, and a 140-Year-Old Naturally Established Neo-Allopolyploid Monkeyflower. Edger et al., Plant Cell 2017 Sep;29(9):2150-2167.)

On the other hand, many natural species did not show obvious genome shock. Although the authors cited a few papers, most relevant for this manuscript would be the allopolyploid **Arabidopsis kamchatica**, which belongs to the same genus as the focal species A. suecica. For example, the title of the following paper explicitly mentioned the lack of stochastic changes in transcriptomes (Genome-wide quantification of homeolog expression ratio revealed nonstochastic gene regulation in synthetic allopolyploid Arabidopsis. Akama et al. Nucleic Acids Res. 2014 Apr;42(6):e46). Loss-of-function mutations were rarely fixed, suggesting that gene loss by genomic shock was not pervasive (Patterns of polymorphism and selection in the subgenomes of the allopolyploid Arabidopsis kamchatica. Paape et al. Nat Commun. 2018 Sep 25;9(1):3909). The authors did cite a recent paper using **Brachypodium** in a different sentence, but it should be discussed in this context (Gradual polyploid genome evolution
revealed by pan-genomic analysis of Brachypodium hybridum and its diploid progenitors. Gordon et al. Nat Commun. 2020 Jul 29;11(1):3670).

In many crop species, genome shock was not drastic or observed, although the authors did not say explicitly all/most crop species (some sentences implicitly imply). In wheat community, I would say it is a common sense that genome shock is not relevant for wheat. Although studies around 2000 suggested genome shock (e.g., Ozkan e t al. Plant Cell 13; 1735, 2001; Shaked et al. Plant Cell 13: 1749, 2001), genome-wide analysis with improved bioinformatics did not show clear evidence. A recent high-quality genome assembly paper by the International Wheat Genome Sequencing Consortium (Science 361: 661, 2018) said that "As reported (18), no evidence was found for a major burst of transposition after polyploidization. The independent evolution in the diploid lineages was reflected in differences in the specific composition of the A, B, and D subgenomes at the subfamily (variants) level". (note that this confirmed a previous Science paper in 2014, its reference 18). There are literatures with or without genome shock in crop **Brassica** species. I hope the authors would find such literatures further.

The current manuscript neglecting these and other relevant references is not suitable for any reasonable journal. The authors should cite these references and refocus the title, abstract and conclusions. Line 54 "However, genomic changes resulting from the hybridization of two (or more) diverged genomes have primarily been studied in crops, such as cotton5, strawberry6, wheat7 and peanut8,9, which confounds polyploidization with domestication." is clearly wrong, see references above. Yet, I strongly suggest that the data and analysis are novel and more than adequate for Nature Ecology and Evolution when rewritten.

We agree that our overview of the existing literature was insufficient and thank the Reviewer for pointing this out. We have extensively revised our Introduction and added many more references, including all the ones suggested. There are surely more that could and should be added, but the literature on polyploidy is huge, as the Reviewer is well aware, and at 164 references, we are probably already at the limit of what the journal will tolerate. Furthermore,

as also noted by the Reviewer, many reported results are of dubious quality because of technical limitations.

We also agree with the reviewer that the literature is probably biased towards results that show some kind of “genomic shock” — which is indeed a poorly defined concept in the literature. Please note that we use it accordingly, as a grab-bag narrative device, to put our results in context of various things that have been said and claimed about polyploid evolution. We do not mean to imply that the field in general believes all these things, nor do we claim that we are the first to find stasis rather than shock (indeed we cite several examples). We have tried to make this clear in our writing throughout.

That said, we still believe there is a certain pattern to these observations: natural polyploids showing signatures of genomic shock tend to be very recent, mostly originating within the last 200 years and may therefore not be completely adapted. Older natural polyploids which have been studied in detail, generally seem to be stable — but there is no doubt that more studies are needed, as we now emphasize in the manuscript.

There is a major methodological concern on transcriptomic analysis in the section 4. In the line 965 of the Material and Methods, the authors mapped the reads directly to the allopolyploid genome using STAR as if it is a diploid species. Recent bioinformatic development showed that such analysis can result in high rate of mapping errors ("cross mapping between the subgenomes" in line 968). When STAR is used, the error rate was roughly 10% in wheat and Arabidopsis (Homeolog expression quantification methods for allopolyploids. Kuo et al. Brief Bioinform. 2020 Mar 23;21(2):395-407), which would affect the estimation of subgenome bias. The effect of the bioinformatic tools on the subgenome bias was also shown recently (Homoeologous gene expression and co-expression network analyses and evolutionary inference in allopolyploids. Hu et al. Brief Bioinform. Online ahead of print 10.1093/bib/bbaa035). For A. suecica, the error rate is probably less, seeing "the substantial (~11,6%)
divergence between the subgenomes" (line 84), but the bias may matter for conserved genes (histone genes of two subgenomes may be highly similar). These literatures indicate several bioinformatic workflows of subgenome-classification approaches for allopolyploid species such as EAGLE-RC, HomeoRoq, GSNAP-PolyCat. The current result should be verified by using such methods.

We agree, and have carefully investigated this issue along the lines suggested. For details, please see Supplementary Figure 13 and the updated Methods section “RNA seq mapping and gene expression analysis”. In brief, RNA-seq reads of A. thaliana *and* A. arenosa *individuals were mixed* in silico *to measure cross-mapping, which was estimated to be ~1% for* A. thaliana *and ~6% for* A. arenosa, *regardless of which pipeline was used (STAR vs HiSAT2 vs Eagle). These values are much lower than when mapping reads to the combined references of* A. thaliana *and* A. lyrata *(~5% and ~11% cross-mapping, respectively).*

The relatively high cross-mapping for A. arenosa *may seem concerning, but is an artifact due to the high level of polymorphism in this outcrossing species, which means that some exons in a particular* A. arenosa *individual are, in fact, closer to their homeologs on the* A.

thaliana *subgenome than to those on the* A. arenosa *subgenome of* A. suecica*. However, diversity within* A. suecica *is massively lower, which means transcripts from the* A. arenosa *subgenome will almost certainly map back to the correct subgenome.*

Additionally, the difference in gene expression for A. arenosa *individuals mapped to just the* A. arenosa *or both subgenomes of* A. suecica *is small, which strengthens our belief that cross-mapping does not significantly affect our results.*

In the section 5, the evidence for "gradual evolution" is not strong although it is in the manuscript title. In the line 350, the authors discussed "For both subgenomes, the first principal component separates A. suecica from the ancestral species and the synthetic hybrid (Fig. 5a,b), suggesting that hybridization does not automatically result in large-scale transcriptional changes, and that altered gene expression changes in natural A. suecica have evolved over time." However, a simple other possibility is that the very diploid individual contributed to the natural allopolyploidization was different from the examined samples, and may have come close to natural hybrid in the PCA plot. Unless obtaining ancient DNA, this is very difficult to test. Other than this analysis, there does not seem direct/strong evidences of "gradual" evolution. The authors should present other evidences, or change the title as well as the abstract and the conclusion (e.g., line 481).

Although we agree that demonstrating selection is in general extremely difficult, and have accordingly tried to be cautious in our claims (saying no more than that our results "suggest" adaptation), we do not fully agree with the main criticism here. In particular there was *no single ancestral individual that served as parent, but rather a population. It is of course possible that we use the wrong comparison nonetheless, but we cannot go much further than comparing multiple individuals of both species (and we note that we contribute to setting a new standard for polyploidy research by doing so — it clearly matters, as our results on "nucleolar dominance" demonstrate). Identifying the particular genes under selection will be very difficult due to extensive linkage disequilibrium in* A. suecica*.*

We also note that our use of "gradual evolution" in the title does not only refer to this section, but to the overall evidence that there has not been dramatic change.

Abstract
Line 38 as well as Line 414. There is a big logical jump between the enrichment of plastid genes and nucleo-cytoplasmic compatibility. I do not think this possibility can be discussed without experimental evidence.

We agree that this is highly speculative, and mention it merely as an interesting hypothesis. We hope that further studies may shed light on the evolution of cyto-nuclear interactions. Based on discussions with an expert, we added an analysis showing that a high fraction of the genes in question are known to be involved in nuclear interactions with the chloroplast based on structural data from protein complexes.

Introduction
Line 67, a space is missing after a period

Fixed.

Results and discussion
Line 82. "naturally inbred A. suecica" is confusing. Do you mean that it selfed in natural populations? If so, show some evidence of inbred status. I imagine the authors would like to mean that A. suecica is a natural species and selfing was conducted in the lab. The same issue in line 71.

Yes, A. suecica *is known to be highly selfing. We note this and have added the relevant citations.*

Fig. 1a. LGM in the middle of tree is ambiguous. If 6-8 mya is shown, time range may be shown, too, because time estimates must be relative depending on calibration points.

Fixed.

Section 3
The text says Figure 4 repeatedly but it must be Figure 3.

Fixed.

The authors discussed the potential reasons of the difference in TE activities of two subgenomes. In literatures, there are several studies showing reduced number, activity or selection of TEs in A. thaliana, which may be associated with the transition to self-fertilization (e.g., The evolution of transposable elements in natural populations of self-fertilizing Arabidopsis thaliana and its outcrossing relative Arabidopsis lyrata. Lockton S, Gaut BS. BMC Evol Biol. 2010 Jan 12;10:10; The predominantly selfing plant Arabidopsis thaliana experienced a recent reduction in transposable element abundance compared to its outcrossing relative Arabidopsis lyrata. de la Chaux et al. Mob DNA. 2012 Feb 7;3(1):2). Although A. lyrata were used in these studies, the authors used A. lyrata as the closest substitute for A. arenosa, and the patterns would be similar. The discussion should include these aspects.

Thank you: we now mention this, and have added the reference.

Lines 214 and 222. "See below" was used repeatedly, but it is far in the text, and the relevance is not explicitly explained below.

Fixed.

Line 230. "the distribution is more similar to that of non-synonymous SNPs than to that of synonymous SNPs (Fig 4b and c)". Seeing the figure, the frequency spectrum of TEs are very different from either non synonymous or synonymous sites (for example, at 0, non-syn 0.1, syn 0.09, TEs 0.24) and thus the sentence is confusing even if it may not be incorrect.

What we are saying is, in fact, correct. We now note that the frequency distribution is "heavily" skewed towards zero — hopefully this makes the comparison clearer.

Line 381-2. The text does not tell the connection between nitrate and Fennoscandinavia.

This was a very speculative idea that nitrate signaling could be altered in the A. thaliana *lineage (and not* A. suecica*) as these species are associated with disturbed agricultural sites, however, we decided to remove this from the manuscript..*

Line 392. Grammatically strange: A. suecica A. thaliana

Fixed.

Line 400, it is too strong to say "must reflect trans-regulation". There may be other complicated scenarios.

Of course: the statement has been weakened.

Line 420. The finding of homeologous exchange is interesting, and this is thanks to the high quality analysis using the model species. It is important to define "genome shock" clearly if the authors decided to continue using it. Some researchers may consider that the changes observed here is a part of genome shock.

We agree that it is interesting, and also that some people would consider the instantaneous loss of ~100 genes shocking. However, as noted above, this is up to them: we are really only using "genome shock" as a narrative device. Our results are what they are.

Supplementary Figures
Line 547, Erecta should be erecta
Line 548, typo, that of and

Fixed.

Materials and Methods
line 791, "We extracted 64g of whole genomic DNA". The amount seems too large, and likely a mistake. As in line 767, DNA may be extracted from tens of grams of tissues.

Yes, thank you! (64g was the weight of the tissue for the DNA extraction)

Reviewer #2 (Remarks to the Author):

This is an interesting and useful paper describing the genome of allopolyploid Arabidopsis suecica. What is especially powerful here, is that the authors provide a comparison not only of the two sub-genomes within A. suecica, but also (1) an assessment of diversity within A. suecica extant accessions and (2) a comparison of both established and newly generated A.

suecica to the parents. This is one of only few attempts to sequence the genome of a non-crop allopolyploid, and the only one I know of to this quality and with the comparison to two extant and well characterised parents, which gives this study quite a bit of power.

I like it, I think it is an important contribution also because it dispels some things that are assumed to be generalities about allopolyploids. The authors (1) generated a high quality genome for A. suecica, (2) showed that A. suecica does not have an extensive "genome shock" history, but they do show evidence of three separate homologous exchange events, (3) showed there is no sub genome dominance either in rRNA loci, or in gene expression, (4) showed the dominance does not vary by tissue as it does in some other species, (5) show there was no abrupt change in gene expression or massive transposon activation and (6) show there was longer term transcriptional adjustment, perhaps adaptive, in established suecica. To me the result that there is variation for the extent of nucleolar dominance was especially cool…

We thank the reviewer for this positive evaluation and encouragement.

I have only some minor points for clarification - and one suggestion for an analysis.

1) Could you please provide lists of the actual DE genes?

Yes, included now as Supplementary dataset 2. We also include the CPM counts for expression in Supplementary Data 3.

2) It would be cool to see a PCA that includes the arenosa sub genome of suecica together with extant diploid and tetraploid A. arenosa to see if the ancestor might have been diploid or tetraploid (as this matters for the conclusion that perhaps the thaliana genome did more meiotic adaptation in suecica). I think this analysis would add to the story, though I don't see it as essential.

We agree that this could be a very interesting analysis, especially given the vast resequencing dataset on A. arenosa *(Monnahan et al. 2019). However, we are aware of an ongoing study from a different group focusing on exactly this question. Given this and the fact that it is not essential to our story, we decided to leave it out. Also, there is evidence obtained from smaller datasets (Novikova et al. 2016; Novikova et al. 2017) that* A. suecica *likely originated from the unreduced gamete of* A. thaliana *and a diploid gamete of a tetraploid* A. arenosa*.*

3) Line 137 - it is not entirely clear what is meant by "lineage-specific genes in A. suecica"

Genes in A. suecica *without a reciprocal best blast hit to* A. thaliana *or* A. lyrata *— now noted in text.*

4) Lines 316-317: this is also true in cotton, should probably cite that - see: Adams et al 2014, Genetics 168:2217-2226.

Done.

Reviewer #3 (Remarks to the Author):

In this study, Burns et al. sequenced natural A. suecica genomes and compared genomic and gene expression changes in the allotetraploids with those of putative progenitors, A. thaliana (At) and A. arenosa (Aa). They concluded that the evolutionary changes in the two subgenomes are gradual with no obvious dominance of subgenomic variation or TE activities but down-played several thousands of differentially expressed genes (DEGs) identified between the At and Aa subgenomes.

We have two separate sections describing transcriptomic patterns in A. suecica*. In Section 4 we compare the expression of homeologous genes from* A. thaliana *and* A. arenosa *subgenomes of* A. suecica*, showing that there is no global dominance of one subgenome over the other — contrary to previous claims. In Section 5 we discuss thousands of DEGs between the orthologous genes in A. thaliana subgenome and A. thaliana itself, as well as between A. arenosa subgenome and A. arenosa. These DEGs show patterns that are suggestive of adaptation.*

Needless to say, there are also many genes that are differentially expressed between the two sub-genomes — as expected given that the parental species diverged ~6 million years ago.

Moreover, they show upregulation of meiosis-related genes and the genes in the cytoplasmic-nuclear related process. The sequence data of A. suecica is of high quality and will be useful resources for the plant genomics and evolution communities.

Thanks: we very much hope this will be the case.

The quality of A. arenosa sequence is poor and should be improved in the future.

Indeed.

It appears that the authors have attempted to emphasise the gradual evolution of the genomes, by mis-interpreting some data with biased views and speculative statements, ignoring many previous reports pertinent to the topic.

We obviously have an opinion, but our results are clearly presented, all the data are available, and readers are welcome to draw their own conclusions. As noted by Reviewer 1, our literature overview left much to be desired, and we have attempted to remedy this.

A major concern is over-interpretation or misinterpretation of their results. Although A. suecica is a naturally formed allotetraploids, itself alone cannot represent a wide range of natural occurring polyploids. In contrast to their claims that genomic reshuffling or dominance occurs only in polyploid crops, it also occurs in Tragopogon miscellus (Chester et al. 2012, PNAS), an allotetraploid formed in natural ecological environments in ~150 years.

The authors have mis-interpreted two sets of important factual data in polyploid literature. First, the perceived “domestication” effect has little or nothing to do with evolutionary and biological consequences on polyploids. There are two kinds of allopolyploids with respect to genomic stability and exchanges. One kind, like A. suecica and cotton allotetraploids, does not show rapid genomic reshuffling; the other type, like synthetic Brassica napus, and newly formed T. miscellus in natural ecological conditions, displays rapid genomic reshuffling. Second, the age of polyploids has little or no effect on genomic exchanges. For example, A. suecica is estimated to form 16,000-300,000 years ago, and allotetraploid cotton was formed ~1.5 million years ago, while hexaploid wheat was formed 8,000-10,000 years, so was B. napus (Lu et al. 2019 Nature Communications). Those natural polyploids formed at different evolutionary time (from 16,000 years to 1.5 million years) plus newly formed allotetraploid cotton (Liu et al. 2001, Genome) are all genetically stable. However, resynthesised B. napus (Xiong et et al. 2011, PNAS) and wheat (Feldman et al. 1997, Genetics) show rapid genomic exchanges. This is probably because the exact progenitors that formed those “stable” polyploids became extinct over time. The extant species available today are not the same progenitors as they were 8,000-10,000 years ago. These misconceptions about the effects of “age” and “domestication” on genomic stability in polyploids should be completely revised to reflect a plethora of data documented in the literature.

We agree that we missed many important citations as was also pointed out by Reviewer 1. We have incorporated all the suggested ones and many more into our manuscript. However, we do not share the Reviewer’s conviction about what is known about polyploid evolution. We agree that the data suggest a correlation between age and stability, but do not think this rules out that domestication plays a role as well. However, all this is rather beside the point: our data and results are what they are, and we make it perfectly clear that you cannot generalize from a single species.

Along the same line, the quote from Charles Darwin (line 493), albeit elegant, does not support their speculation on polyploidy and should be removed.

We softened the statement but left the quote, because we, as authors, think it illustrates our conclusions quite nicely.

Comments on data and technical validity

1. Lines 88-89, “Our attempt to assemble a tetraploid A. arenosa, which is also included here, led to a very fragmented assembly of 3,629 contigs with an N50 of 331 Kb.” This quality score of A. arenosa genome is poor. As a result, they should tone down the statements on comparative data with A. arenosa, especially in the area of structural variations, genomic synteny, and TE activities. This should also apply to syntenic relationships between A. arenosa and A. thaliana or A. lyrata (Figure 1b, d).

Almost all analyses in this manuscript are based on the A. arenosa *subgenome of* A. suecica*, which is high quality. The* A. arenosa *(species) assembly (which is indeed very poor) was only used in: (1) the assembly of* A. suecica *to help to differentiate between the contigs of the* A.

thaliana and the A. arenosa subgenomes(but not the scaffolding process);, (2) the identification of species-specific rDNA (section 2), and; (3) assignment of the ancestry of the TEs for the cross-jumping analysis (section 3).

2. rDNA dominance. This part of data is confusing or misleading. Data in Fig. 2a did not show a clear pattern of A. arenosa rDNA expression dominance over A. thaliana rDNA, in a phenomenon known as nucleolar dominance, as cited in Pontes et al. (2004, PNAS), but many other reports were not cited. Instead, they seem to co-express, which is not supported by numerous other reports. It is also odd that they noted loss of the majority of A. thaliana rDNA copies in AS90a line. Based on the FISH images in Fig. 2d, 2e, the large staining area of rDNA clusters (from A. arenosa?) disappeared, compared to data in Fig. 2b, 2c. The small clusters in Fig. 2b-e should be A. thaliana (instead of A. arenosa), based on data presentation in Fib. 2b, 2c. It is misleading if the probe cross-hybridised both A. thaliana and A. arenosa rDNA, how can the Aa and At NORs be discriminated, as the centromere signals were not clearly indicative?

We have tried to make the figure clearer, and have added more citations. We do indeed see co-expression in most individuals, as discussed in the text. The FISH images have been improved, and we note that they are meant to complement the sequencing results. We have now improved the layout of the figure to make it more clear. FISH figures 2d and 2e clearly show that the large clusters of 45S rDNA in ASS3 are located on A. thaliana chromosomes (marked by the A. thaliana-specific centromeric satellite). In contrast, FISH figures 2b and 2c demonstrate that the large rDNA clusters on A. thaliana chromosomes are missing in AS90a.

3. When describing TE specificity, they should use “specific” or “unique”, and TEs should be specific (instead of “private”) to A. thaliana or A. arenosa, etc. Because the TE annotation in A. arenosa could be over- or under-estimated due to poor genome assemblies, the difference between Aa and At subgenomes could be more than just a “bottleneck.” Although the analysis is justified, the conclusion has exaggerated the similarities and ignored the differences. What is the difference between “A. arenosa in A. suecica” and “A. arenosa in A. suecica (4n) in Fig. 3a?

We changed “private” to “unique”, as suggested. The TE annotation was not based on the (poor) A. arenosa genome assembly (see above), but we mention that repetitive parts are under-represented nonetheless, and hence TEs will have been missed. The “A. arenosa in A. suecica (4n)” entry is explained in both text and figure caption.

4. Expression bias or dominance (Fig. 4). They referenced A. thaliana, Zea mays, B. rapa, and G. raimondii (lines 282-283), all of which were “paleopolyploids” or ancient autopolyploids (though exact origin in some like Z. mays is unknown). These are very different from A. suecica, an allotetraploid. They should appropriately reference results from many other allopolyploids. A simple Google search would give them proper references needed.

Done.

4. The expression results suggest that overall the two subgenomes are expressed at similar levels, this does not preclude that some genes can exhibit expression bias between homoeologs. Indeed, they identified some homoeologs that show expression bias in Supplemental Fig. 14b, c, especially in resynthesised A. suecica. The other possibility is that heat maps and violin plots in separate subgenomes cannot identify those expression bias of individual homoeolog between the subgenomes. For example, if one set of 1,000 At genes were overexpressed, while another set of 1,000 Aa genes was upregulated, the overall plots would be similar. Indeed, the conclusion of no expression bias in this section is inconsistent with the expression evolution (3,800-4,100 DEGs in Aa or At subgenomes, a total of ~8,000 DEGs!!!) among different A. suecica lines (see below). Alternative methods such as using nonadditive expression values (different from the average of the two parents), especially in synthetic A. suecica, would help resolve this issue.

As already noted above, section 4 is about testing the hypothesis of an overall bias, motivated by published results that such a bias exists. Section 5 is about expression evolution in A. suecica*, and we identify over 8k DEGs.*

Certainly other kinds of analysis are possible, but we note that this is already a very long paper. Our paper and data will hopefully enable others to do their own analyses.

5. Expression evolution in A. suecica. The PCA plots clearly showed expression divergence between A. thaliana and A. arenosa in these analyses (Fig. 5). “The total number of DEGs was 3,854 and 4,136 genes for the A. thaliana and A. arenosa subgenomes, respectively (see Methods).” (Lines 373-374). Notably, these numbers of DEGs are similar to several previous reports in this polyploid species, which the authors did not cite. This along with a major concern and others about misinterpretation of their data, leading to their biased perception of “no difference.” While A. suecica subgenomes are stable and colinear as they are, like other stable polyploids, the differences, especially in gene expression and phenotypic traits between A. suecica and A. thaliana or A. arenosa, are there and should be reported and discussed.

We are not aware of any other papers that have done an analysis comparable to what we did in this section. If the Reviewer can provide references, we will of course happily cite them. We also do not understand the concern about “differences”. The section in question reports over 8k genes that appear to have changed expression in A. suecica*! Of course there are also expression differences between* A. thaliana *and* A. arenosa*, but that is not what this paper is about.*

In summary, the genomic sequences of A. suecica have been improved from their previous work (Novikova et al. 2017 MBE) and should provide a good resource for the plant genomics and evolutionary biology communities. The quality of A. areonosa sequence is poor and should be improved. Throughout the manuscript, the data are over- or mis-interpreted with a one-sided (biased) view of polyploid evolution, which is also reflected in their reference citations. The quote from C. Darwin (line 493) does not support their scope of study, nor is consistent with a plethora of data available in the literature and should be removed.

The quote in question is not meant to "support [our] scope of study", nor is it meant to be "consistent with a plethora of data available in the literature". It is meant to summarize our conclusions based on our data. No more, no less.

Decision Letter, first revision:

4th March 2021

*Please ensure you delete the link to your author homepage in this e-mail if you wish to forward it to your co-authors.

Dear Magnus,

Your revised manuscript entitled "Gradual evolution of allopolyploidy in <i>Arabidopsis suecica</i>" has now been seen by two of our original reviewers, whose comments are attached. Reviewer 3 was unavailable to look at the revision but Reviewer 1 has generously commented on your responses to their comments too.

The reviewers agree that the revision is improved but Reviewer 1 still has a number of concerns which will need to be addressed before we can offer publication in Nature Ecology & Evolution. We will therefore need to see your responses to the criticisms raised and to some editorial concerns, along with a revised manuscript, before we can reach a final decision regarding publication.

Please see the attached document with comments from Reviewer 1 (the new comments are in red colour).

I should point out three main concerns that we share with the reviewer: 1) the need to work on the introduction to better contextualize and set up the questions addressed. 2) the need to clearly define the terms used (for example 'overall bias','genome shock' and 'gradual evolution'so the results and conclusions are clear. 3) we generally ask authors to refrain from using quotes and in this case, we agree with both reviewers that the Darwin quote is unwarranted.

We therefore invite you to revise your manuscript taking into account all reviewer and editor comments. Please highlight all changes in the manuscript text file.

We are committed to providing a fair and constructive peer-review process. Do not hesitate to contact us if there are specific requests from the reviewers that you believe are technically impossible or unlikely to yield a meaningful outcome.

When revising your manuscript:

* Include a "Response to reviewers" document detailing, point-by-point, how you addressed each reviewer comment. If no action was taken to address a point, you must provide a compelling argument. This response will be sent back to the reviewers along with the revised manuscript.

* If you have not done so already please begin to revise your manuscript so that it conforms to our Article format instructions at http://www.nature.com/natecolevol/info/final-submission. Refer also to any guidelines provided in this letter.

* Include a revised version of any required reporting checklist. It will be available to referees (and,

potentially, statisticians) to aid in their evaluation if the manuscript goes back for peer review. A revised checklist is essential for re-review of the paper.

Please use the link below to submit your revised manuscript and related files:

[REDACTED]

Note: This URL links to your confidential home page and associated information about manuscripts you may have submitted, or that you are reviewing for us. If you wish to forward this email to co-authors, please delete the link to your homepage.

We hope to receive your revised manuscript within four to eight weeks. If you cannot send it within this time, please let us know. We will be happy to consider your revision so long as nothing similar has been accepted for publication at Nature Ecology & Evolution or published elsewhere.

Nature Ecology & Evolution is committed to improving transparency in authorship. As part of our efforts in this direction, we are now requesting that all authors identified as 'corresponding author' on published papers create and link their Open Researcher and Contributor Identifier (ORCID) with their account on the Manuscript Tracking System (MTS), prior to acceptance. ORCID helps the scientific community achieve unambiguous attribution of all scholarly contributions. You can create and link your ORCID from the home page of the MTS by clicking on 'Modify my Springer Nature account'. For more information please visit please visit www.springernature.com/orcid.

Please do not hesitate to contact me if you have any questions or would like to discuss these revisions further.

We look forward to seeing the revised manuscript and thank you for the opportunity to review your work.

[REDACTED]

Reviewers' comments:

Reviewer #1 (Remarks to the Author):

I got a challenging task to check the responses to the criticisms by the reviewer 3 in addition to mine, and would try below. This resulted in a fairly long document, and I wish this would be helpful. New comments are shown following "Reviewer 1".

Reviewer #1
The authors focused on the allopolyploid Arabidopsis suecica to study the genomic and transcriptomic effect of allopolyploidization. Since around 2000, many studies reported a large scale and stochastic genomic, epigenetic and transcriptional changes following polyploidization, termed "genome shock" (originally coined by McClintock in a different context). Here the authors examined whether such changes were observed. They conducted a diverse array of analysis including genome assembly, synteny, rDNA clusters, transposable elements in population samples, test of expression bias between

subgenomes, and the comparison of expression between diploid and polyploid species. After all, they did not find "genome shock" (the definition should be more explicit, though). I do not think this is a negative result, but provide a solid data to understand the evolution. Many of the results are novel. Section 3 of transposable elements using population samples is particularly interesting. The chromosome level substitutions in the section 6 was identified thanks to "unparalleled in its resolution thanks to one of the parents being a major model species" (line 479). I would suggest the manuscript can become suitable for Nature Ecology and Evolution.
We thank the Reviewer for these encouraging remarks and respond to the issues alluded to in what follows.
However, there are major issues to be revised. In the abstract, introduction and discussions, the central theme of the authors is:
genome shock was observed in crop polyploid species, but natural species are not well studied in this aspect (e.g., lines 20, 54, 492)
This dichotomy of crop and natural species is based on a shallow coverage of previous literatures by the authors. This question needs to be refocused or different questions should be addressed. Genome shock was fashionable about 20 years ago, but most of later studies did not support it possibly because of species difference or of improved bioinformatic analysis. Unfortunately, there is a bias in the references because studies showing genome shock tend to receive attentions in high rank journals while those without genome shock may be considered negative results. Due to this reason, the genome shock may not be mentioned in the title or in abstract, and thus one may need to read the text. Below I will mention some of representative cases below.
In Abstract, Line 24 said "It is unclear, however, whether domesticated polyploids are representative of natural ones." However, using natural species, there are both types of studies with or without genome shock. For example, on the one hand, many papers on natural contemporary polyploid Tragopogon discussed transcriptomic shock and gene loss (for example, Transcriptomic shock generates evolutionary novelty in a newly formed, natural allopolyploid plant. Buggs et al. Curr Biol. 2011 Apr 12;21(7):551-6); Gene loss and silencing in Tragopogon miscellus (Asteraceae): comparison of natural and synthetic allotetraploids. Buggs et al. Heredity (Edinb). 2009 Jul;103(1):73-81.). There is a relatively recent paper on Mimulus (Subgenome Dominance in an Interspecific Hybrid, Synthetic Allopolyploid, and a 140-Year-Old Naturally Established Neo-Allopolyploid Monkeyflower. Edger et al., Plant Cell 2017 Sep;29(9):2150-2167.)
On the other hand, many natural species did not show obvious genome shock. Although the authors cited a few papers, most relevant for this manuscript would be the allopolyploid Arabidopsis kamchatica, which belongs to the same genus as the focal species A. suecica. For example, the title of the following paper explicitly mentioned the lack of stochastic changes in transcriptomes (Genome-wide quantification of homeolog expression ratio revealed nonstochastic gene regulation in synthetic allopolyploid Arabidopsis. Akama et al. Nucleic Acids Res. 2014 Apr;42(6):e46). Loss-of-function mutations were rarely fixed, suggesting that gene loss by genomic shock was not pervasive (Patterns of polymorphism and selection in the subgenomes of the allopolyploid Arabidopsis kamchatica. Paape et al. Nat Commun. 2018 Sep 25;9(1):3909). The authors did cite a recent paper using Brachypodium in a different sentence, but it should be discussed in this context (Gradual polyploid genome evolution revealed by pan-genomic analysis of Brachypodium hybridum and its diploid progenitors. Gordon et al. Nat Commun. 2020 Jul 29;11(1):3670).
In many crop species, genome shock was not drastic or observed, although the authors did not say explicitly all/most crop species (some sentences implicitly imply). In wheat community, I would say it is a common sense that genome shock is not relevant for wheat. Although studies around 2000 suggested genome shock (e.g., Ozkan et al. Plant Cell 13; 1735, 2001; Shaked et al. Plant Cell 13: 1749, 2001), genome-wide analysis with improved bioinformatics did not show clear evidence. A

recent high-quality genome assembly paper by the International Wheat Genome Sequencing Consortium (Science 361: 661, 2018) said that "As reported (18), no evidence was found for a major burst of transposition after polyploidization. The independent evolution in the diploid lineages was reflected in differences in the specific composition of the A, B, and D subgenomes at the subfamily (variants) level". (note that this confirmed a previous Science paper in 2014, its reference 18). There are literatures with or without genome shock in crop Brassica species. I hope the authors would find such literatures further.
The current manuscript neglecting these and other relevant references is not suitable for any reasonable journal. The authors should cite these references and refocus the title, abstract and conclusions. Line 54 "However, genomic changes resulting from the hybridization of two (or more) diverged genomes have primarily been studied in crops, such as cotton5, strawberry6, wheat7 and peanut8,9, which confounds polyploidization with domestication." is clearly wrong, see references above. Yet, I strongly suggest that the data and analysis are novel and more than adequate for Nature Ecology and Evolution when rewritten.
We agree that our overview of the existing literature was insufficient and thank the Reviewer for pointing this out. We have extensively revised our Introduction and added many more references, including all the ones suggested. There are surely more that could and should be added, but the literature on polyploidy is huge, as the Reviewer is well aware, and at 164 references, we are probably already at the limit of what the journal will tolerate. Furthermore, as also noted by the Reviewer, many reported results are of dubious quality because of technical limitations.
We also agree with the reviewer that the literature is probably biased towards results that show some kind of "genomic shock" — which is indeed a poorly defined concept in the literature. Please note that we use it accordingly, as a grab-bag narrative device, to put our results in context of various things that have been said and claimed about polyploid evolution. We do not mean to imply that the field in general believes all these things, nor do we claim that we are the first to find stasis rather than shock (indeed we cite several examples). We have tried to make this clear in our writing throughout.
That said, we still believe there is a certain pattern to these observations: natural polyploids showing signatures of genomic shock tend to be very recent, mostly originating within the last 200 years and may therefore not be completely adapted. Older natural polyploids which have been studied in detail, generally seem to be stable — but there is no doubt that more studies are needed, as we now emphasize in the manuscript.

Reviewer 1: A similar point was raised by the reviewer 3, and so will be commented there.

There is a major methodological concern on transcriptomic analysis in the section 4. In the line 965 of the Material and Methods, the authors mapped the reads directly to the allopolyploid genome using STAR as if it is a diploid species. Recent bioinformatic development showed that such analysis can result in high rate of mapping errors ("cross mapping between the subgenomes" in line 968). When STAR is used, the error rate was roughly 10% in wheat and Arabidopsis (Homeolog expression quantification methods for allopolyploids. Kuo et al. Brief Bioinform. 2020 Mar 23;21(2):395-407), which would affect the estimation of subgenome bias. The effect of the bioinformatic tools on the subgenome bias was also shown recently (Homoeologous gene expression and co-expression network analyses and evolutionary inference in allopolyploids. Hu et al. Brief Bioinform. Online ahead of print 10.1093/bib/bbaa035). For A. suecica, the error rate is probably less, seeing "the substantial (~11,6%) divergence between the subgenomes" (line 84), but the bias may matter for conserved genes (histone genes of two subgenomes may be highly similar). These literatures indicate several bioinformatic workflows of subgenome-classification approaches for allopolyploid species such as EAGLE-RC, HomeoRoq, GSNAP-PolyCat. The current result should be verified by using such methods.

We agree, and have carefully investigated this issue along the lines suggested. For details, please see Supplementary Figure 13 and the updated Methods section "RNA seq mapping and gene expression analysis". In brief, RNA-seq reads of A. thaliana and A. arenosa individuals were mixed in silico to measure cross-mapping, which was estimated to be ~1% for A . thaliana and ~6% for A. arenosa, regardless of which pipeline was used (STAR vs HiSAT2 vs Eagle). These values are much lower than when mapping reads to the combined references of A . thaliana and A. lyrata (~5% and ~11% cross-mapping, respectively).
The relatively high cross-mapping for A. arenosa may seem concerning, but is an artifact due to the high level of polymorphism in this outcrossing species, which means that some exons in a particular A. arenosa individual are, in fact, closer to their homeologs on the A. thaliana subgenome than to those on the A. arenosa subgenome of A. suecica. However, diversity within A . suecica is massively lower, which means transcripts from the A. arenosa subgenome will almost certainly map back to the correct subgenome.
Additionally, the difference in gene expression for A. arenosa individuals mapped to just the A. arenosa or both subgenomes of A . suecica is small, which strengthens our belief that cross-mapping does not significantly affect our results.

Reviewer 1: The additional analysis provided a quantitative assessment of this issue. The analysis is fine. The authors just need to describe the adequate details in the Method section to increase reproducibility. For example, the pipeline names and references are not found in the methods, although briefly mentioned in this reply letter. There should not be a strict restriction in the number of references in the Method section. This should be also mentioned briefly in the result (current mention in the result is in a different context). The 6% error of the A. arenosa may not be negligible and would be a topic for future studies, but I think that it is fine in this manuscript as far as it is explicitly quantified.

In the section 5, the evidence for "gradual evolution" is not strong although it is in the manuscript title. In the line 350, the authors discussed "For both subgenomes, the first principal component separates A. suecica from the ancestral species and the synthetic hybrid (Fig. 5a,b), suggesting that hybridization does not automatically result in large-scale transcriptional changes, and that altered gene expression changes in natural A. suecica have evolved over time." However, a simple other possibility is that the very diploid individual contributed to the natural allopolyploidization was different from the examined samples, and may have come close to natural hybrid in the PCA plot. Unless obtaining ancient DNA, this is very difficult to test. Other than this analysis, there does not seem direct/strong evidences of "gradual" evolution. The authors should present other evidences, or change the title as well as the abstract and the conclusion (e.g., line 481).
Although we agree that demonstrating selection is in general extremely difficult, and have accordingly tried to be cautious in our claims (saying no more than that our results "suggest" adaptation), we do not fully agree with the main criticism here. In particular there was no single ancestral individual that served as parent, but rather a population. It is of course possible that we use the wrong comparison nonetheless, but we cannot go much further than comparing multiple individuals of both species (and we note that we contribute to setting a new standard for polyploidy research by doing so — it clearly matters, as our results on "nucleolar dominance" demonstrate). Identifying the particular genes under selection will be very difficult due to extensive linkage disequilibrium in A . suecica.
We also note that our use of "gradual evolution" in the title does not only refer to this section, but to the overall evidence that there has not been dramatic change.

Reviewer 1: A similar point was raised by reviewer 3. I am afraid that the authors did not understand the point and instead addressed something different. The point was not about selection/adaptation at all. I totally agree that "there was no single ancestral individual that served as parent, but rather a population." "but we cannot go much further than comparing multiple individuals of both species". I agree that the authors did the best with available samples. However, there is an inherent difficulty in comparing a subgenome and its diploid counterpart, and the data cannot provide a direct evidence. The reviewer 3 raised a relevant point and thus will be continued below.
In my opinion, these points do not need additional experiments and are ready to be addressed.

Abstract
Line 38 as well as Line 414. There is a big logical jump between the enrichment of plastid genes and nucleo-cytoplasmic compatibility. I do not think this possibility can be discussed without experimental evidence.
We agree that this is highly speculative, and mention it merely as an interesting hypothesis. We hope that further studies may shed light on the evolution of cyto-nuclear interactions. Based on discussions with an expert, we added an analysis showing that a high fraction of the genes in question are known to be involved in nuclear interactions with the chloroplast based on structural data from protein complexes.

Reviewer 1: I do not think this strengthens the manuscript, but it is fine if the authors would like to keep it.

Introduction
Line 67, a space is missing after a period
Fixed.

Reviewer 1: fine

Results and discussion
Line 82. "naturally inbred A. suecica" is confusing. Do you mean that it selfed in natural populations? If so, show some evidence of inbred status. I imagine the authors would like to mean that A. suecica is a natural species and selfing was conducted in the lab. The same issue in line 71.
Yes, A. suecica is known to be highly selfing. We note this and have added the relevant citations.

Reviewer 1: The authors addressed the point adequately.

Fig. 1a. LGM in the middle of tree is ambiguous. If 6-8 mya is shown, time range may be shown, too, because time estimates must be relative depending on calibration points.
Fixed.

Reviewer 1: fine

Section 3
The text says Figure 4 repeatedly but it must be Figure 3.
Fixed.

Reviewer 1: fine

The authors discussed the potential reasons of the difference in TE activities of two subgenomes. In literatures, there are several studies showing reduced number, activity or selection of TEs in A. thaliana, which may be associated with the transition to self-fertilization (e.g., The evolution of transposable elements in natural populations of self-fertilizing Arabidopsis thaliana and its outcrossing relative Arabidopsis lyrata. Lockton S, Gaut BS. BMC Evol Biol. 2010 Jan 12;10:10; The predominantly selfing plant Arabidopsis thaliana experienced a recent reduction in transposable element abundance compared to its outcrossing relative Arabidopsis lyrata. de la Chaux et al. Mob DNA. 2012 Feb 7;3(1):2). Although A. lyrata were used in these studies, the authors used A. lyrata as the closest substitute for A. arenosa, and the patterns would be similar. The discussion should include these aspects.
Thank you: we now mention this, and have added the reference.

Reviewer 1: The authors addressed the point adequately.

Lines 214 and 222. "See below" was used repeatedly, but it is far in the text, and the relevance is not explicitly explained below.
Fixed.

Reviewer 1: fine

Line 230. "the distribution is more similar to that of non-synonymous SNPs than to that of synonymous SNPs (Fig 4b and c)". Seeing the figure, the frequency spectrum of TEs are very different from either non synonymous or synonymous sites (for example, at 0, non-syn 0.1, syn 0.09, TEs 0.24) and thus the sentence is confusing even if it may not be incorrect.
What we are saying is, in fact, correct. We now note that the frequency distribution is "heavily" skewed towards zero — hopefully this makes the comparison clearer.

Reviewer 1: fine

Line 381-2. The text does not tell the connection between nitrate and Fennoscandinavia.
This was a very speculative idea that nitrate signaling could be altered in the A. thaliana lineage (and not A . suecica) as these species are associated with disturbed agricultural sites, however, we decided to remove this from the manuscript..

Reviewer 1: fine

Line 392. Grammatically strange: A. suecica A. thaliana
Fixed.

Reviewer 1: fine

Line 400, it is too strong to say "must reflect trans-regulation". There may be other complicated scenarios.
Of course: the statement has been weakened.

Reviewer 1: fine

Line 420. The finding of homeologous exchange is interesting, and this is thanks to the high quality analysis using the model species. It is important to define "genome shock" clearly if the authors decided to continue using it. Some researchers may consider that the changes observed here is a part of genome shock.
We agree that it is interesting, and also that some people would consider the instantaneous loss of ~100 genes shocking. However, as noted above, this is up to them: we are really only using "genome shock" as a narrative device. Our results are what they are.

Reviewer 1: This is relevant to a comment by reviewer 3 and so will be discussed below.

Supplementary Figures
Line 547, Erecta should be erecta Line 548, typo, that of and
Fixed.

Reviewer 1: fine

Materials and Methods
line 791, "We extracted 64g of whole genomic DNA". The amount seems too large, and likely a mistake. As in line 767, DNA may be extracted from tens of grams of tissues.
Yes, thank you! (64g was the weight of the tissue for the DNA extraction)

Reviewer 1: fine

Reviewer #3 (Remarks to the Author):
In this study, Burns et al. sequenced natural A. suecica genomes and compared genomic and gene expression changes in the allotetraploids with those of putative progenitors, A. thaliana (At) and A. arenosa (Aa). They concluded that the evolutionary changes in the two subgenomes are gradual with no obvious dominance of subgenomic variation or TE activities but down-played several thousands of differentially expressed genes (DEGs) identified between the At and Aa subgenomes.
We have two separate sections describing transcriptomic patterns in A. suecica. In Section 4 we compare the expression of homeologous genes from A . thaliana and A . arenosa subgenomes of A. suecica, showing that there is no global dominance of one subgenome over the other — contrary to previous claims. In Section 5 we discuss thousands of DEGs between the orthologous genes in A. thaliana subgenome and A. thaliana itself, as well as between A. arenosa subgenome and A. arenosa. These DEGs show patterns that are suggestive of adaptation.
Needless to say, there are also many genes that are differentially expressed between the two sub-genomes — as expected given that the parental species diverged ~6 million years ago.
Moreover, they show upregulation of meiosis-related genes and the genes in the cytoplasmic-nuclear related process. The sequence data of A. suecica is of high quality and will be useful resources for the plant genomics and evolution communities.
Thanks: we very much hope this will be the case.
The quality of A. arenosa sequence is poor and should be improved in the future.
Indeed.
It appears that the authors have attempted to emphasise the gradual evolution of the genomes, by

mis-interpreting some data with biased views and speculative statements, ignoring many previous reports pertinent to the topic.
We obviously have an opinion, but our results are clearly presented, all the data are available, and readers are welcome to draw their own conclusions. As noted by Reviewer 1, our literature overview left much to be desired, and we have attempted to remedy this.

Reviewer 1: Text until here are general comments and thus I will comment specific comments below.

A major concern is over-interpretation or misinterpretation of their results. Although A. suecica is a naturally formed allotetraploids, itself alone cannot represent a wide range of natural occurring polyploids. In contrast to their claims that genomic reshuffling or dominance occurs only in polyploid crops, it also occurs in Tragopogon miscellus (Chester et al. 2012, PNAS), an allotetraploid formed in natural ecological environments in ~150 years.
The authors have mis-interpreted two sets of important factual data in polyploid literature. First, the perceived "domestication" effect has little or nothing to do with evolutionary and biological consequences on polyploids. There are two kinds of allopolyploids with respect to genomic stability and exchanges. One kind, like A. suecica and cotton allotetraploids, does not show rapid genomic reshuffling; the other type, like synthetic Brassica napus, and newly formed T. miscellus in natural ecological conditions, displays rapid
genomic reshuffling. Second, the age of polyploids has little or no effect on genomic exchanges. For example, A. suecica is estimated to form 16,000-300,000 years ago, and allotetraploid cotton was formed ~1.5 million years ago, while hexaploid wheat was formed 8,000-10,000 years, so was B. napus (Lu et al. 2019 Nature Communications). Those natural polyploids formed at different evolutionary time (from 16,000 years to 1.5 million years) plus newly formed allotetraploid cotton (Liu et al. 2001, Genome) are all genetically stable. However, resynthesised B. napus (Xiong et et al. 2011, PNAS) and wheat (Feldman et al. 1997, Genetics) show rapid genomic exchanges. This is probably because the exact progenitors that formed those "stable" polyploids became extinct over time. The extant species available today are not the same progenitors as they were 8,000-10,000 years ago. These misconceptions about the effects of "age" and "domestication" on genomic stability in polyploids should be completely revised to reflect a plethora of data documented in the literature.
We agree that we missed many important citations as was also pointed out by Reviewer 1. We have incorporated all the suggested ones and many more into our manuscript. However, we do not share the Reviewer's conviction about what is known about polyploid evolution. We agree that the data suggest a correlation between age and stability, but do not think this rules out that domestication plays a role as well. However, all this is rather beside the point: our data and results are what they are, and we make it perfectly clear that you cannot generalize from a single species.

Reviewer 1: Here the reviewer 3 raised a few critical points but I am afraid the authors did not answer some of them. I will discuss one by one.
Point 1 by the reviewer 3: In contrast to their claims that genomic reshuffling or dominance occurs only in polyploid crops, it also occurs in Tragopogon miscellus (Chester et al. 2012, PNAS), an allotetraploid formed in natural ecological environments in ~150 years.
Reviewer 1: This point was also raised by me. The authors added the relevant literatures accordingly.

Point 2 by the reviewer 3: First, the perceived "domestication" effect has little or nothing to do with evolutionary and biological consequences on polyploids.
Authors: but do not think this rules out that domestication plays a role as well.

Reviewer 1: The authors' quote above is true. However, it is not the point as far as I read the comment by the reviewer 3. First, crop polyploid species did not always show a large genomic changes, and natural polyploid species were not always stable. There may be a tendency, but there are clearly many exceptions to the authors' statements. A similar point was also raised by me. Second, the authors did not present data of domesticated species in this manuscript. The authors showed that A. suecica did not show the large changes observed in crop species, thus so any conclusions on crop species would be indirect. In the current manuscript, large genomic changes in crop species is the central issue of the introduction (as well as of the abstract and conclusion to a less extent). Nothing ruled out the role of domestication, and I myself agree that it has a role (although not the single large factor). However, this would not be a suitable framework of the presented data. The concern was not about number of works cited, but rather the lack of connection of the existing literature to the study. The presentation of the body of literature, while full of great examples, does not set the reader up for the what study is about nor does it represent the existing literature in a clear in a way that shows the authors' point of view of the literature.

Point 3 by the reviewer 3: The time issue, including the fact "This is probably because the exact progenitors that formed those "stable" polyploids became extinct over time. The extant species available today are not the same progenitors as they were 8,000-10,000 years ago."
Authors: there is no clear response to this point.

Reviewer 1: See the comment in the section of reviewer 1.

Reviewer 3:
Along the same line, the quote from Charles Darwin (line 493), albeit elegant, does not support their speculation on polyploidy and should be removed.
We softened the statement but left the quote, because we, as authors, think it illustrates our conclusions quite nicely.

Reviewer 1: To my taste, I do not feel that the Darwin's quote is suitable, but this is in the range of the authors' decision.

Reviewer 3:
Comments on data and technical validity
1. Lines 88-89, "Our attempt to assemble a tetraploid A. arenosa, which is also included here, led to a very fragmented assembly of 3,629 contigs with an N50 of 331 Kb." This quality score of A. arenosa genome is poor. As a result, they should tone down the statements on comparative data with A. arenosa, especially in the area of structural variations, genomic synteny, and TE activities. This should also apply to syntenic relationships between A. arenosa and A. thaliana or A. lyrata (Figure 1b, d).
Almost all analyses in this manuscript are based on the A . arenosa subgenome of A. suecica, which is high quality. The A. arenosa (species) assembly (which is indeed very poor) was only used in: (1) the assembly of A. suecica to help to differentiate between the contigs of the A. thaliana and the A . arenosa subgenomes(but not the scaffolding process);, (2) the identification of species-specific rDNA (section 2), and; (3) assignment of the ancestry of the TEs for the cross-jumping analysis (section 3).

Reviewer 1: The authors' response is valid. It may be good idea to explain more clearly.

Reviewer 3:
2. rDNA dominance. This part of data is confusing or misleading. Data in Fig. 2a did not show a clear pattern of A. arenosa rDNA expression dominance over A. thaliana rDNA, in a phenomenon known as nucleolar dominance, as cited in Pontes et al. (2004, PNAS), but many other reports were not cited. Instead, they seem to co-express, which is not supported by numerous other reports. It is also odd that they noted loss of the majority of A. thaliana rDNA copies in AS90a line. Based on the FISH images in Fig. 2d, 2e, the large staining area of rDNA clusters (from A. arenosa?) disappeared, compared to data in Fig. 2b, 2c. The small clusters in Fig. 2b-e should be A. thaliana (instead of A. arenosa), based on data presentation in Fib. 2b, 2c. It is misleading if the probe cross-hybridised both A. thaliana and A. arenosa rDNA, how can the Aa and At NORs be discriminated, as the centromere signals were not clearly indicative?
We have tried to make the figure clearer, and have added more citations. We do indeed see co-expression in most individuals, as discussed in the text. The FISH images have been improved, and we note that they are meant to complement the sequencing results. We have now improved the layout of the figure to make it more clear. FISH figures 2d and 2e clearly show that the large clusters of 45S rDNA in ASS3 are located on A . thaliana chromosomes (marked by the A. thaliana-specific centromeric satellite). In contrast, FISH figures 2b and 2c demonstrate that the large rDNA clusters on A. thaliana chromosomes are missing in AS90a.

Reviewer 1: I think that the authors addressed the point.

Reviewer 3:
3. When describing TE specificity, they should use "specific" or "unique", and TEs should be specific (instead of "private") to A. thaliana or A. arenosa, etc. Because the TE annotation in A. arenosa could be over- or under-estimated due to poor genome assemblies, the difference between Aa and At subgenomes could be more than just a "bottleneck." Although the analysis is justified, the conclusion has exaggerated the similarities and ignored the differences. What is the difference between "A. arenosa in A. suecica" and "A. arenosa in A. suecica (4n) in Fig. 3a?
We changed "private" to "unique", as suggested. The TE annotation was not based on the (poor) A . arenosa genome assembly (see above), but we mention that repetitive parts are under-represented nonetheless, and hence TEs will have been missed. The "A. arenosa in A. suecica (4n)" entry is explained in both text and figure caption.

Reviewer 1: The authors addressed the point.

Reviewer 3:
4. Expression bias or dominance (Fig. 4). They referenced A. thaliana, Zea mays, B. rapa, and G. raimondii (lines 282-283), all of which were "paleopolyploids" or ancient autopolyploids (though exact origin in some like Z. mays is unknown). These are very different from A. suecica, an allotetraploid. They should appropriately reference results from many other allopolyploids. A simple Google search would give them proper references needed.
Done.

Reviewer 1: The authors addressed the point.

Reviewer 3:
4. The expression results suggest that overall the two subgenomes are expressed at similar levels, this does not preclude that some genes can exhibit expression bias between homoeologs. Indeed, they identified some homoeologs that show expression bias in Supplemental Fig. 14b, c, especially in resynthesised A. suecica. The other possibility is that heat maps and violin plots in separate subgenomes cannot identify those expression bias of individual homoeolog between the subgenomes. For example, if one set of 1,000 At genes were overexpressed, while another set of 1,000 Aa genes was upregulated, the overall plots would be similar. Indeed, the conclusion of no expression bias in this section is inconsistent with the expression evolution (3,800-4,100 DEGs in Aa or At subgenomes, a total of ~8,000 DEGs!!!) among different A. suecica lines (see below). Alternative methods such as using nonadditive expression values (different from the average of the two parents), especially in synthetic A. suecica, would help resolve this issue.
As already noted above, section 4 is about testing the hypothesis of an overall bias, motivated by published results that such a bias exists. Section 5 is about expression evolution in A . suecica, and we identify over 8k DEGs.
Certainly other kinds of analysis are possible, but we note that this is already a very long paper. Our paper and data will hopefully enable others to do their own analyses.

Reviewer 1: I found that the point raised by the reviewer 3 and the reply by the authors are not matching. The authors need to define "overall bias" explicitly. I see the implicit definition by the authors. If I did not misunderstand, no deviation from zero in the Figure 4 would mean no bias for the authors. However, it is not the only possible definition. The reviewer 3 asked "For example, if one set of 1,000 At genes were overexpressed, while another set of 1,000 Aa genes was upregulated, the overall plots would be similar". This is defined as a bias for the reviewer 3. A simple term "overall bias" can mean both. From my point of view, either definition is fine as far as defined clearly, and the results of the Figure 4 is interesting and would be enough for a conclusion. However, the text should be clarified.
This is a fundamental point of this manuscript. The definitions of key concepts are obscure at least in the text. Thus, the contents are ambiguous and misleading. The authors might feel that the data were quantitatively shown. However, if the sentences are obscure, it is not understood and even wrong conclusions will spread. I would recommend authors to think and write critically/quantitatively the definition of key technical terms.
In this sense, this point is yet to be addressed by the authors.

Reviewer 3:
5. Expression evolution in A. suecica. The PCA plots clearly showed expression divergence between A. thaliana and A. arenosa in these analyses (Fig. 5). "The total number of DEGs was 3,854 and 4,136 genes for the A. thaliana and A. arenosa subgenomes, respectively (see Methods)." (Lines 373-374). Notably, these numbers of DEGs are similar to several previous reports in this polyploid species, which the authors did not cite. This along with a major concern and others about misinterpretation of their data, leading to their biased perception of "no difference." While A. suecica subgenomes are stable and colinear as they are, like other stable polyploids, the differences, especially in gene expression and phenotypic traits between A. suecica and A. thaliana or A. arenosa, are there and should be reported and discussed.
We are not aware of any other papers that have done an analysis comparable to what we did in this

section. If the Reviewer can provide references, we will of course happily cite them. We also do not understand the concern about “differences”. The section in question reports over 8k genes that appear to have changed expression in A. suecica! Of course there are also expression differences between A . thaliana and A . arenosa, but that is not what this paper is about.

Reviewer 1: For the first part of the comment, I cannot raise the actual example instead of the reviewer 3. However, even if this first part is not explained in detail by the reviewer 3, the authors should have addressed the latter part. I can understand easily the concern about "differences", or I would be surprised why the authors do not understand to respond.
This is possibly the most fundamental issue of this manuscript. Let me summarize the authors findings.
1. synteny at the chromosomal level - no change
(Here, the section title "1. The genome is conserved" is highly misleading. It can mean that gene orders are conserved, coding sequences are conserved, etc. It must be specific.).
2. rDNAs - highly variable
3. transposable element - no major change
4. subgenome-wide expression bias (i.e., Fig. 4) - no change
5.1. Expression levels between diploid and allotetraploid, trans-regulation - changes (468 genes)
note: such trans-regulation may have happened instantaneously at the time of polyploidization so probably not gradual. Some of the references the authors cited already showed this point.
5.2. Expression levels between diploid and allotetraploid, meiotic and plastid-nuclear related genes - changes
6. Mb-scale homeologous exchange - changes
note: see my comment on line 420
From these results, the abstract and the conclusion had the following sentences.
Abstract line 29 "we see no evidence of a genome shock",
Conclusion line 825, " we also find no evidence for "genome shock" (i.e. major genomic changes linked to structural and functional changes)

Here, I understand that the reviewer 3 are not persuaded by the conclusion "no major changes". I wish the authors see this point clearly now.
In the revised version, the authors defined genome shock vaguely at the end of the manuscript (conclusion, line 825, “genome shock” (i.e. major genomic changes linked to structural and functional changes). This definition of "genome shock" by the authors are limited to what they did not find difference, and is not commonly shared, or objective.
This is to some extent a semantic issue. It may not be productive to summarize all this point into "genome shock" vs. "no major change". Nor gradual vs. instantaneous.
The authors said in the reply:
We agree that it is interesting, and also that some people would consider the instantaneous loss of ~100 genes shocking. However, as noted above, this is up to them: we are really only using “genome shock” as a narrative device. Our results are what they are.
That is what I wish the authors did. Unfortunately, in the current version, for example, the two sentences in the abstract and conclusion (copied above) are not at all a "narrative device" but highly confusing. I wish the authors can revise accordingly.

The list of the six results above is also relevant to consider the issue of "gradual" evolution, which the reviewer 3 also mentioned. In the current structure, I cannot see much evidence. Results 1, 3, 4

showed no changes in essence. Result 5.1 was probably instantaneous. Results 2 and 6 can be either instantaneous or gradual (more likely former). Regarding Result 5.2, there is an inherent difficulty in obtaining the very diploid ancestral samples, and the authors' reply was "We also note that our use of "gradual evolution" in the title does not only refer to this section, but to the overall evidence that there has not been dramatic change."
The authors reply to reviewer 1 implies the problem ("We also note that our use of "gradual evolution" in the title does not only refer to this section, but to the overall evidence that there has not been dramatic change."). The lack of dramatic change does not automatically mean gradual changes. At most, it can indirectly serve as an elimination method. Gradual would mean there were multiple steps, but the presented data cannot provide direct data of intermediate steps. Some of supporting data (for example, site frequency spectrum) may be a better evidence of gradual evolution, but it is not explicitly explained in the current manuscript. To keep this term in the title, abstract and conclusions, the authors should define gradual evolution quantitatively, and make summary sentences in the conclusion. My suggestion is rather not stick to gradual evolution because there are adequate interesting results.
By the way, I noticed that the first submission said 3854 and 4136 genes, but the revised version said 4186 and 4571 genes (line 561). Why? I could not find the explicit reason in this reply letter. It must be explained (error in the first analysis?).

Reviewer 3:
In summary, the genomic sequences of A. suecica have been improved from their previous work (Novikova et al. 2017 MBE) and should provide a good resource for the plant genomics and evolutionary biology communities. The quality of A. areonosa sequence is poor and should be improved. Throughout the manuscript, the data are over- or mis-interpreted with a one-sided (biased) view of polyploid evolution, which is also reflected in their reference citations. The quote from C. Darwin (line 493) does not support their scope of study, nor is consistent with a plethora of data available in the literature and should be removed.
The quote in question is not meant to "support [our] scope of study", nor is it meant to be "consistent with a plethora of data available in the literature". It is meant to summarize our conclusions based on our data. No more, no less.

Reviewer 1: The same as a point above.

Reviewer #2 (Remarks to the Author):

This paper looks fine to me. That authors addressed all my comments.

********************END********************

Author Rebuttal, first revision:

Response to Reviewer(s)

Reviewer #1 (Remarks to the Author):

I got a challenging task to check the responses to the criticisms by the reviewer 3 in addition to mine, and would try below. This resulted in a fairly long document, and I wish this would be helpful. New comments are shown following "Reviewer 1".

Many thanks for taking the time to look at this again, and trying to provide constructive comments. We feel the remaining issues are mostly semantic, and have tried to clarify them. Your new comments are in blue, and our response in red.

Reviewer #1
The authors focused on the allopolyploid Arabidopsis suecica to study the genomic and transcriptomic effect of allopolyploidization. Since around 2000, many studies reported a large scale and stochastic genomic, epigenetic and transcriptional changes following polyploidization, termed "genome shock" (originally coined by McClintock in a different context). Here the authors examined whether such changes were observed. They conducted a diverse array of analysis including genome assembly, synteny, rDNA clusters, transposable elements in population samples, test of expression bias between subgenomes, and the comparison of expression between diploid and polyploid species. After all, they did not find "genome shock" (the definition should be more explicit, though). I do not think this is a negative result, but provide a solid data to understand the evolution. Many of the results are novel. Section 3 of transposable elements using population samples is particularly interesting. The
chromosome level substitutions in the section 6 was identified thanks to "unparalleled in its resolution thanks to one of the parents being a major model species" (line 479). I would suggest the manuscript can become suitable for Nature Ecology and Evolution.
We thank the Reviewer for these encouraging remarks and respond to the issues alluded to in what follows.
However, there are major issues to be revised. In the abstract, introduction and discussions, the central theme of the authors is:
genome shock was observed in crop polyploid species, but natural species are not well studied in this aspect (e.g., lines 20, 54, 492)
This dichotomy of crop and natural species is based on a shallow coverage of previous literatures by the authors. This question needs to be refocused or different questions should be addressed. Genome shock was fashionable about 20 years ago, but most of later studies did not support it possibly because of species difference or of improved bioinformatic analysis. Unfortunately, there is a bias in the references because studies

showing genome shock tend to receive attentions in high rank journals while those without genome shock may be considered negative results. Due to this reason, the genome shock may not be mentioned in the title or in abstract, and thus one may need to read the text. Below I will mention some of representative cases below.
In Abstract, Line 24 said "It is unclear, however, whether domesticated polyploids are representative of natural ones." However, using natural species, there are both types of studies with or without genome shock. For example, on the one hand, many papers on natural contemporary polyploid Tragopogon discussed transcriptomic shock and gene loss (for example, Transcriptomic shock generates evolutionary novelty in a newly formed, natural allopolyploid plant. Buggs et al. Curr Biol. 2011 Apr 12;21(7):551-6); Gene loss and silencing in Tragopogon miscellus (Asteraceae): comparison of natural and synthetic allotetraploids. Buggs et al. Heredity (Edinb). 2009 Jul;103(1):73-81.). There is a relatively recent paper on Mimulus (Subgenome Dominance in an Interspecific Hybrid, Synthetic Allopolyploid, and a 140-Year-Old Naturally Established Neo-Allopolyploid Monkeyflower. Edger et al., Plant Cell 2017 Sep;29(9):2150-2167.)
On the other hand, many natural species did not show obvious genome shock. Although the authors cited a few papers, most relevant for this manuscript would be the allopolyploid Arabidopsis kamchatica, which belongs to the same genus as the focal species A. suecica. For example, the title of the following paper explicitly mentioned the lack of stochastic changes in transcriptomes (Genome-wide quantification of homeolog expression ratio revealed nonstochastic gene regulation in synthetic allopolyploid Arabidopsis. Akama et al. Nucleic Acids Res. 2014 Apr;42(6):e46). Loss-of-function mutations were rarely fixed, suggesting that gene loss by genomic shock was not pervasive (Patterns of polymorphism and selection in the subgenomes of the allopolyploid Arabidopsis kamchatica. Paape et al. Nat Commun. 2018 Sep 25;9(1):3909). The authors did cite a recent paper using Brachypodium in a different sentence, but it should be discussed in this context (Gradual polyploid genome evolution
revealed by pan-genomic analysis of Brachypodium hybridum and its diploid progenitors. Gordon et al. Nat Commun. 2020 Jul 29;11(1):3670).
In many crop species, genome shock was not drastic or observed, although the authors did not say explicitly all/most crop species (some sentences implicitly imply). In wheat community, I would say it is a common sense that genome shock is not relevant for wheat. Although studies around 2000 suggested genome shock (e.g., Ozkan e t al. Plant Cell 13; 1735, 2001; Shaked et al. Plant Cell 13: 1749, 2001), genome-wide analysis with improved bioinformatics did not show clear evidence. A recent high-quality genome assembly paper by the International Wheat Genome Sequencing Consortium (Science 361: 661, 2018) said that "As reported (18), no evidence was found for a major burst of transposition after polyploidization. The independent evolution in the diploid lineages was reflected in differences in the specific composition of the A, B, and D

subgenomes at the subfamily (variants) level". (note that this confirmed a previous Science paper in 2014, its reference 18). There are literatures
with or without genome shock in crop Brassica species. I hope the authors would find such literatures further.
The current manuscript neglecting these and other relevant references is not suitable for any reasonable journal. The authors should cite these references and refocus the title, abstract and conclusions. Line 54 "However, genomic changes resulting from the hybridization of two (or more) diverged genomes have primarily been studied in crops, such as cotton5, strawberry6, wheat7 and peanut8,9, which confounds polyploidization with domestication." is clearly wrong, see references above. Yet, I strongly suggest that the data and analysis are novel and more than adequate for Nature Ecology and Evolution when rewritten.
We agree that our overview of the existing literature was insufficient and thank the Reviewer for pointing this out. We have extensively revised our Introduction and added many more references, including all the ones suggested. There are surely more that could and should be added, but the literature on polyploidy is huge, as the Reviewer is well aware, and at 164 references, we are probably already at the limit of what the journal will tolerate. Furthermore, as also noted by the Reviewer, many reported results are of dubious quality because of technical limitations.
We also agree with the reviewer that the literature is probably biased towards results that show some kind of "genomic shock" — which is indeed a poorly defined concept in the literature. Please note that we use it accordingly, as a grab-bag narrative device, to put our results in context of various things that have been said and claimed about polyploid evolution. We do not mean to imply that the field in general believes all these things, nor do we claim that we are the first to find stasis rather than shock (indeed we cite several examples). We have tried to make this clear in our writing throughout.
That said, we still believe there is a certain pattern to these observations: natural polyploids showing signatures of genomic shock tend to be very recent, mostly originating within the last 200 years and may therefore not be completely adapted. Older natural polyploids which have been studied in detail, generally seem to be stable — but there is no doubt that more studies are needed, as we now emphasize in the manuscript.

Reviewer 1: A similar point was raised by the reviewer 3, and so will be commented there.

There is a major methodological concern on transcriptomic analysis in the section 4. In the line 965 of the Material and Methods, the authors mapped the reads directly to the allopolyploid genome using STAR as if it is a diploid species. Recent bioinformatic development showed that such analysis can result in high rate of mapping errors ("cross

mapping between the subgenomes" in line 968). When STAR is used, the error rate was roughly 10% in wheat and Arabidopsis (Homeolog expression quantification methods for allopolyploids. Kuo et al. Brief Bioinform. 2020 Mar 23;21(2):395-407), which would affect the estimation of subgenome bias. The effect of the bioinformatic tools on the subgenome bias was also shown recently (Homoeologous gene expression and co-expression network analyses and evolutionary inference in allopolyploids. Hu et al. Brief Bioinform. Online ahead of print 10.1093/bib/bbaa035). For A. suecica, the error rate is probably less, seeing "the substantial (~11,6%)
divergence between the subgenomes" (line 84), but the bias may matter for conserved genes (histone genes of two subgenomes may be highly similar). These literatures indicate several bioinformatic workflows of subgenome-classification approaches for allopolyploid species such as EAGLE-RC, HomeoRoq, GSNAP-PolyCat. The current result should be verified by using such methods.
We agree, and have carefully investigated this issue along the lines suggested. For details, please see Supplementary Figure 13 and the updated Methods section "RNA seq mapping and gene expression analysis". In brief, RNA-seq reads of A. thaliana and A. arenosa individuals were mixed in silico to measure cross-mapping, which was estimated to be ~1% for A . thaliana and ~6% for A. arenosa, regardless of which pipeline was used (STAR vs HiSAT2 vs Eagle). These values are much lower than when mapping reads to the combined references of A . thaliana and A. lyrata (~5% and ~11% cross-mapping, respectively).
The relatively high cross-mapping for A. arenosa may seem concerning, but is an artifact due to the high level of polymorphism in this outcrossing species, which means that some exons in a particular A. arenosa individual are, in fact, closer to their homeologs on the A. thaliana subgenome than to those on the A. arenosa subgenome of A. suecica. However, diversity within A . suecica is massively lower, which means transcripts from the A. arenosa subgenome will almost certainly map back to the correct subgenome.
Additionally, the difference in gene expression for A. arenosa individuals mapped to just the A. arenosa or both subgenomes of A . suecica is small, which strengthens our belief that cross-mapping does not significantly affect our results.

Reviewer 1: The additional analysis provided a quantitative assessment of this issue. The analysis is fine. The authors just need to describe the adequate details in the Method section to increase reproducibility. For example, the pipeline names and references are not found in the methods, although briefly mentioned in this reply letter. There should not be a strict restriction in the number of references in the Method section. This should be also mentioned briefly in the result (current mention in the result is in a different context). The 6% error of the A. arenosa may not be negligible and

would be a topic for future studies, but I think that it is fine in this manuscript as far as it is explicitly quantified.

We agree. All this has been clarified in the Methods section.

In the section 5, the evidence for "gradual evolution" is not strong although it is in the manuscript title. In the line 350, the authors discussed "For both subgenomes, the first principal component separates A. suecica from the ancestral species and the synthetic hybrid (Fig. 5a,b), suggesting that hybridization does not automatically result in large-scale transcriptional changes, and that altered gene expression changes in natural A. suecica have evolved over time." However, a simple other possibility is that the very diploid individual contributed to the natural allopolyploidization was different from the examined samples, and may have come close to natural hybrid in the PCA plot. Unless obtaining ancient DNA, this is very difficult to test. Other than this analysis, there does not seem direct/strong evidences of "gradual" evolution. The authors should present other evidences, or change the title as well as the abstract and the conclusion (e.g., line 481).

Although we agree that demonstrating selection is in general extremely difficult, and have accordingly tried to be cautious in our claims (saying no more than that our results "suggest" adaptation), we do not fully agree with the main criticism here. In particular there was no single ancestral individual that served as parent, but rather a population. It is of course possible that we use the wrong comparison nonetheless, but we cannot go much further than comparing multiple individuals of both species (and we note that we contribute to setting a new standard for polyploidy research by doing so — it clearly matters, as our results on "nucleolar dominance" demonstrate). Identifying the particular genes under selection will be very difficult due to extensive linkage disequilibrium in A . suecica.

We also note that our use of "gradual evolution" in the title does not only refer to this section, but to the overall evidence that there has not been dramatic change.

Reviewer 1: A similar point was raised by reviewer 3. I am afraid that the authors did not understand the point and instead addressed something different. The point was not about selection/adaptation at all. I totally agree that "there was no single ancestral individual that served as parent, but rather a population." "but we cannot go much further than comparing multiple individuals of both species". I agree that the authors did the best with available samples. However, there is an inherent difficulty in comparing a subgenome and its diploid counterpart, and the data cannot provide a direct evidence. The reviewer 3 raised a relevant point and thus will be continued below.

In my opinion, these points do not need additional experiments and are ready to be addressed.

We agree. We simply report what the available data show.

Line 420. The finding of homeologous exchange is interesting, and this is thanks to the high quality analysis using the model species. It is important to define "genome shock" clearly if the authors decided to continue using it. Some researchers may consider that the changes observed here is a part of genome shock.
We agree that it is interesting, and also that some people would consider the instantaneous loss of ~100 genes shocking. However, as noted above, this is up to them: we are really only using "genome shock" as a narrative device. Our results are what they are.

Reviewer 1: This is relevant to a comment by reviewer 3 and so will be discussed below.

Reviewer #3 (Remarks to the Author):
In this study, Burns et al. sequenced natural A. suecica genomes and compared genomic and gene expression changes in the allotetraploids with those of putative progenitors, A. thaliana (At) and A. arenosa (Aa). They concluded that the evolutionary changes in the two subgenomes are gradual with no obvious dominance of subgenomic variation or TE activities but down-played several thousands of differentially expressed genes (DEGs) identified between the At and Aa subgenomes.
We have two separate sections describing transcriptomic patterns in A. suecica. In Section 4 we compare the expression of homeologous genes from A . thaliana and A . arenosa subgenomes of A. suecica, showing that there is no global dominance of one subgenome over the other — contrary to previous claims. In Section 5 we discuss thousands of DEGs between the orthologous genes in A. thaliana subgenome and A. thaliana itself, as well as between A. arenosa subgenome and A. arenosa. These DEGs show patterns that are suggestive of adaptation.
Needless to say, there are also many genes that are differentially expressed between the two sub-genomes — as expected given that the parental species diverged ~6 million years ago.
Moreover, they show upregulation of meiosis-related genes and the genes in the cytoplasmic-nuclear related process. The sequence data of A. suecica is of high quality and will be useful resources for the plant genomics and evolution communities.
Thanks: we very much hope this will be the case.
The quality of A. arenosa sequence is poor and should be improved in the future.
Indeed.

It appears that the authors have attempted to emphasise the gradual evolution of the genomes, by mis-interpreting some data with biased views and speculative statements, ignoring many previous reports pertinent to the topic.
We obviously have an opinion, but our results are clearly presented, all the data are available, and readers are welcome to draw their own conclusions. As noted by Reviewer 1, our literature overview left much to be desired, and we have attempted to remedy this.

Reviewer 1: Text until here are general comments and thus I will comment specific comments below.

A major concern is over-interpretation or misinterpretation of their results. Although A. suecica is a naturally formed allotetraploids, itself alone cannot represent a wide range of natural occurring polyploids. In contrast to their claims that genomic reshuffling or dominance occurs only in polyploid crops, it also occurs in Tragopogon miscellus (Chester et al. 2012, PNAS), an allotetraploid formed in natural ecological environments in ~150 years.
The authors have mis-interpreted two sets of important factual data in polyploid literature. First, the perceived "domestication" effect has little or nothing to do with evolutionary and biological consequences on polyploids. There are two kinds of allopolyploids with respect to genomic stability and exchanges. One kind, like A. suecica and cotton allotetraploids, does not show rapid genomic reshuffling; the other type, like synthetic Brassica napus, and newly formed T. miscellus in natural ecological conditions, displays rapid
genomic reshuffling. Second, the age of polyploids has little or no effect on genomic exchanges. For example, A. suecica is estimated to form 16,000-300,000 years ago, and allotetraploid cotton was formed ~1.5 million years ago, while hexaploid wheat was formed 8,000-10,000 years, so was B. napus (Lu et al. 2019 Nature Communications). Those natural polyploids formed at different evolutionary time (from 16,000 years to 1.5 million years) plus newly formed allotetraploid cotton (Liu et al. 2001, Genome) are all genetically stable. However, resynthesised B. napus (Xiong et et al. 2011, PNAS) and wheat (Feldman et al. 1997, Genetics) show rapid genomic exchanges. This is probably because the exact progenitors that formed those "stable" polyploids became extinct over time. The extant species available today are not the same progenitors as they were 8,000-10,000 years ago. These misconceptions about the effects of "age" and "domestication" on genomic stability in polyploids
should be completely revised to reflect a plethora of data documented in the literature.
We agree that we missed many important citations as was also pointed out by Reviewer 1. We have incorporated all the suggested ones and many more into our manuscript. However, we do not share the Reviewer's conviction about what is known about

polyploid evolution. We agree that the data suggest a correlation between age and stability, but do not think this rules out that domestication plays a role as well. However, all this is rather beside the point: our data and results are what they are, and we make it perfectly clear that you cannot generalize from a single species.

Reviewer 1: Here the reviewer 3 raised a few critical points but I am afraid the authors did not answer some of them. I will discuss one by one.
Point 1 by the reviewer 3: In contrast to their claims that genomic reshuffling or dominance occurs only in polyploid crops, it also occurs in Tragopogon miscellus (Chester et al. 2012, PNAS), an allotetraploid formed in natural ecological environments in ~150 years.
Reviewer 1: This point was also raised by me. The authors added the relevant literatures accordingly.

Point 2 by the reviewer 3: First, the perceived "domestication" effect has little or nothing to do with evolutionary and biological consequences on polyploids.
Authors: but do not think this rules out that domestication plays a role as well.
Reviewer 1: The authors' quote above is true. However, it is not the point as far as I read the comment by the reviewer 3. First, crop polyploid species did not always show a large genomic changes, and natural polyploid species were not always stable. There may be a tendency, but there are clearly many exceptions to the authors' statements. A similar point was also raised by me. Second, the authors did not present data of domesticated species in this manuscript. The authors showed that A. suecica did not show the large changes observed in crop species, thus so any conclusions on crop species would be indirect. In the current manuscript, large genomic changes in crop species is the central issue of the introduction (as well as of the abstract and conclusion to a less extent). Nothing ruled out the role of domestication, and I myself agree that it has a role (although not the single large factor). However, this would not be a suitable framework of the presented data.
The concern was not about number of works cited, but rather the lack of connection of the existing literature to the study. The presentation of the body of literature, while full of great examples, does not set the reader up for the what study is about nor does it represent the existing literature in a clear in a way that shows the authors' point of view of the literature.

This study is about genomic change in *A. suecica*, which is *one* example of polyploid evolution, and may or may not be representative of polyploid evolution. We feel we have been crystal clear about this throughout, but we added one more sentence to this effect to the Introduction. We cite lots of other examples of polyploid evolution, and list what

these studies have found. We do not believe it serves any purpose to get into arguments about general principles of evolution based on the data available.

Point 3 by the reviewer 3: The time issue, including the fact "This is probably because the exact progenitors that formed those “stable” polyploids became extinct over time. The extant species available today are not the same progenitors as they were 8,000-10,000 years ago."
Authors: there is no clear response to this point.

Reviewer 1: See the comment in the section of reviewer 1.

Reviewer 3:
Along the same line, the quote from Charles Darwin (line 493), albeit elegant, does not support their speculation on polyploidy and should be removed.
We softened the statement but left the quote, because we, as authors, think it illustrates our conclusions quite nicely.

Reviewer 1: To my taste, I do not feel that the Darwin's quote is suitable, but this is in the range of the authors' decision.

We agree. But the editors didn’t like it either, so we removed it.

Reviewer 3:
Comments on data and technical validity
1. Lines 88-89, “Our attempt to assemble a tetraploid A. arenosa, which is also included here, led to a very fragmented assembly of 3,629 contigs with an N50 of 331 Kb.” This quality score of A. arenosa genome is poor. As a result, they should tone down the statements on comparative data with A. arenosa, especially in the area of structural variations, genomic synteny, and TE activities. This should also apply to syntenic relationships between A. arenosa and A. thaliana or A. lyrata (Figure 1b, d).
Almost all analyses in this manuscript are based on the A . arenosa subgenome of A. suecica, which is high quality. The A. arenosa (species) assembly (which is indeed very poor) was only used in: (1) the assembly of A. suecica to help to differentiate between the contigs of the A. thaliana and the A . arenosa subgenomes(but not the scaffolding process);, (2) the identification of species-specific rDNA (section 2), and; (3) assignment of the ancestry of the TEs for the cross-jumping analysis (section 3).

Reviewer 1: The authors' response is valid. It may be good idea to explain more clearly.

Reviewer 3:
4. The expression results suggest that overall the two subgenomes are expressed at similar levels, this does not preclude that some genes can exhibit expression bias between homoeologs. Indeed, they identified some homoeologs that show expression bias in Supplemental Fig. 14b, c, especially in resynthesised A. suecica. The other possibility is that heat maps and violin plots in separate subgenomes cannot identify those expression bias of individual homoeolog between the subgenomes. For example, if one set of 1,000 At genes were overexpressed, while another set of 1,000 Aa genes was upregulated, the overall plots would be similar. Indeed, the conclusion of no expression bias in this section is inconsistent with the expression evolution (3,800-4,100 DEGs in Aa or At subgenomes, a total of ~8,000 DEGs!!!) among different A. suecica lines (see below). Alternative methods such as using nonadditive expression values (different from the average of the two parents), especially in synthetic A. suecica, would help resolve this issue.
As already noted above, section 4 is about testing the hypothesis of an overall bias, motivated by published results that such a bias exists. Section 5 is about expression evolution in A . suecica, and we identify over 8k DEGs.
Certainly other kinds of analysis are possible, but we note that this is already a very long paper. Our paper and data will hopefully enable others to do their own analyses.

Reviewer 1: I found that the point raised by the reviewer 3 and the reply by the authors are not matching. The authors need to define "overall bias" explicitly. I see the implicit definition by the authors. If I did not misunderstand, no deviation from zero in the Figure 4 would mean no bias for the authors. However, it is not the only possible definition. The reviewer 3 asked "For example, if one set of 1,000 At genes were overexpressed, while another set of 1,000 Aa genes was upregulated, the overall plots would be similar". This is defined as a bias for the reviewer 3. A simple term "overall bias" can mean both. From my point of view, either definition is fine as far as defined clearly, and the results of the Figure 4 is interesting and would be enough for a conclusion. However, the text should be clarified.

We respectfully disagree. We use “bias” in the standard statistical sense, and it is absolutely clear what we are talking about. The usage is the same as in previous papers about subgenome dominance. Reviewer 3 objects that we don’t show that there aren’t *some* genes with biased expression, which is of course true, but completely beside the point. The section in question is about subgenome dominance, which has a clear definition and has been the topic of multiple papers.

It should also be noted that previous authors *have* reported evidence for subgenome dominance, which is the reason we address it. We strongly believe this was due to poor data, and say so in the paper.

This is a fundamental point of this manuscript. The definitions of key concepts are obscure at least in the text. Thus, the contents are ambiguous and misleading. The authors might feel that the data were quantitatively shown. However, if the sentences are obscure, it is not understood and even wrong conclusions will spread. I would recommend authors to think and write critically/quantitatively the definition of key technical terms.
In this sense, this point is yet to be addressed by the authors.

We agree that clarity is important and have gone through the paper multiple times with this in mind.

Reviewer 3:
5. Expression evolution in A. suecica. The PCA plots clearly showed expression divergence between A. thaliana and A. arenosa in these analyses (Fig. 5). “The total number of DEGs was 3,854 and 4,136 genes for the A. thaliana and A. arenosa subgenomes, respectively (see Methods).” (Lines 373-374). Notably, these numbers of DEGs are similar to several previous reports in this polyploid species, which the authors did not cite. This along with a major concern and others about misinterpretation of their data, leading to their biased perception of “no difference.” While A. suecica subgenomes are stable and colinear as they are, like other stable polyploids, the differences, especially in gene expression and phenotypic traits between A. suecica and A. thaliana or A. arenosa, are there and should be reported and discussed.
We are not aware of any other papers that have done an analysis comparable to what we did in this section. If the Reviewer can provide references, we will of course happily cite them. We also do not understand the concern about “differences”. The section in question reports over 8k genes that appear to have changed expression in A. suecica! Of course there are also expression differences between A . thaliana and A . arenosa, but that is not what this paper is about.

Reviewer 1: For the first part of the comment, I cannot raise the actual example instead of the reviewer 3. However, even if this first part is not explained in detail by the reviewer 3, the authors should have addressed the latter part. I can understand easily the concern about "differences", or I would be surprised why the authors do not understand to respond.

This is possibly the most fundamental issue of this manuscript. Let me summarize the authors findings.
1. synteny at the chromosomal level - no change
(Here, the section title "1. The genome is conserved" is highly misleading. It can mean that gene orders are conserved, coding sequences are conserved, etc. It must be specific.).

It is a title, by necessity brief. It is not misleading, certainly not “highly misleading”: as the corresponding section makes clear, the genome is conserved in all the senses listed. At the sequence level, the subgenomes appear indistinguishable from the parental genomes.

2. rDNAs - highly variable
3. transposable element - no major change
4. subgenome-wide expression bias (i.e., Fig. 4) - no change
5.1. Expression levels between diploid and allotetraploid, trans-regulation - changes (468 genes)
note: such trans-regulation may have happened instantaneously at the time of polyploidization so probably not gradual. Some of the references the authors cited already showed this point.
5.2. Expression levels between diploid and allotetraploid, meiotic and plastid-nuclear related genes - changes
6. Mb-scale homeologous exchange - changes
note: see my comment on line 420
From these results, the abstract and the conclusion had the following sentences.
Abstract line 29 "we see no evidence of a genome shock",
Conclusion line 825, " we also find no evidence for "genome shock" (i.e. major genomic changes linked to structural and functional changes)

Here, I understand that the reviewer 3 are not persuaded by the conclusion "no major changes". I wish the authors see this point clearly now.
In the revised version, the authors defined genome shock vaguely at the end of the manuscript (conclusion, line 825, “genome shock” (i.e. major genomic changes linked to structural and functional changes). This definition of "genome shock" by the authors are limited to what they did not find difference, and is not commonly shared, or objective. This is to some extent a semantic issue. It may not be productive to summarize all this point into "genome shock" vs. "no major change". Nor gradual vs. instantaneous.
The authors said in the reply:
We agree that it is interesting, and also that some people would consider the instantaneous loss of ~100 genes shocking. However, as noted above, this is up to

,

them: we are really only using “genome shock” as a narrative device. Our results are what they are.
That is what I wish the authors did. Unfortunately, in the current version, for example, the two sentences in the abstract and conclusion (copied above) are not at all a "narrative device" but highly confusing. I wish the authors can revise accordingly.

Again we respectfully disagree. The quote from the Abstract is out of context. There is no sentence “we see no evidence of a genome shock” — there is a sentence with a clause that ends this way, *followed by a colon and list that makes it clear what we mean by this*. The sentence in the Conclusions also defines what we mean (as you already note). The phrase “genome shock” is widely used, and poorly defined, which is precisely why we define it when we use it. The alternative would be avoiding it altogether, but we feel this would really be ahistorical.

The list of the six results above is also relevant to consider the issue of "gradual" evolution, which the reviewer 3 also mentioned. In the current structure, I cannot see much evidence. Results 1, 3, 4 showed no changes in essence. Result 5.1 was probably instantaneous. Results 2 and 6 can be either instantaneous or gradual (more likely former). Regarding Result 5.2, there is an inherent difficulty in obtaining the very diploid ancestral samples, and the authors' reply was "We also note that our use of “gradual evolution” in the title does not only refer to this section, but to the overall evidence that there has not been dramatic change."
The authors reply to reviewer 1 implies the problem ("We also note that our use of “gradual evolution” in the title does not only refer to this section, but to the overall evidence that there has not been dramatic change."). The lack of dramatic change does not automatically mean gradual changes. At most, it can indirectly serve as an elimination method. Gradual would mean there were multiple steps, but the presented data cannot provide direct data of intermediate steps. Some of supporting data (for example, site frequency spectrum) may be a better evidence of gradual evolution, but it is not explicitly explained in the current manuscript. To keep this term in the title, abstract and conclusions, the authors should define gradual evolution quantitatively, and make summary sentences in the conclusion. My suggestion is rather not stick to gradual evolution because there are adequate interesting results.

We would argue that since evolution in general is gradual (in the sense of Darwin), it is, in fact, the default, and it is rather instantaneous evolution that would require positive proof. We show that there is no evidence of sudden change in *A. suecica*. But there is no reason to go into philosophy here. As with genome shock, it is clear why we talk of gradual evolution in the Discussion. We explain what we mean, and present the

evidence. We hope this stimulates further discussion — hopefully soon at some meeting over beer!

By the way, I noticed that the first submission said 3854 and 4136 genes, but the revised version said 4186 and 4571 genes (line 561). Why? I could not find the explicit reason in this reply letter. It must be explained (error in the first analysis?).

Yes, we used an updated version of the genome in the revision. This was noted: no qualitative changes resulted.

Reviewer 3:
In summary, the genomic sequences of A. suecica have been improved from their previous work (Novikova et al. 2017 MBE) and should provide a good resource for the plant genomics and evolutionary biology communities. The quality of A. areonosa sequence is poor and should be improved. Throughout the manuscript, the data are over- or mis-interpreted with a one-sided (biased) view of polyploid evolution, which is also reflected in their reference citations. The quote from C. Darwin (line 493) does not support their scope of study, nor is consistent with a plethora of data available in the literature and should be removed.
The quote in question is not meant to “support [our] scope of study”, nor is it meant to be “consistent with a plethora of data available in the literature”. It is meant to summarize our conclusions based on our data. No more, no less.

Reviewer 1: The same as a point above.

Decision Letter, second revision:

29th April 2021

*Please ensure you delete the link to your author homepage in this e-mail if you wish to forward it to your co-authors.

Dear Magnus,

We have now discussed your revised manuscript entitled "Gradual evolution of allopolyploidy in <i>Arabidopsis suecica</i>" as well as your responses to Reviewer #1. We see that you disagree on some points and after careful consideration we agreed that the manuscript needs a few revisions:

1. We believe that 'gradual evolution' in the title is vague and uninformative since it's the default mode of evolution. We suggest a descriptive title: 'Genomic consequences of allopolyploidization in Arabidopsis suecica'.

2. Both referees take issue with claims of no 'genome shock' and 'gradual evolution'. While we see that part of the problem is semantic, we also think that you should give equal emphasis to the results that show differences between different ploidies. We believe this issue needs to be addressed with the following changes:

a) define 'genome shock' in the Abstract and Introduction just like you do in the Discussion.

b) In the Abstract, include the results that show changes between ploidies as listed by the reviewer: rDNAs, Expression levels between diploid and allotetraploid, trans-regulation, Expression levels between diploid and allotetraploid, meiotic and plastid-nuclear related genes, Mb-scale homeologous exchange. In the Discussion, give emphasis to these results as well.
When rewriting the Abstract, please keep in mind that there is a 200 word limit.

3. We agree that it is clear that you talk about bias in the context of subgenome dominance but for clarity, please include a brief definition of overall bias, even if just in terms of referring to the literature.

We therefore invite you to revise your manuscript taking into account these comments. Please highlight all changes in the manuscript text file using track changes in Microsoft Word.

When revising your manuscript:

* Include a response to the editorial requests.

* If you have not done so already please begin to revise your manuscript so that it conforms to our Article format instructions at http://www.nature.com/natecolevol/info/final-submission. Refer also to

any guidelines provided in this letter.

* Include a revised version of any required reporting checklist. It will be available to referees (and, potentially, statisticians) to aid in their evaluation if the manuscript goes back for peer review. A revised checklist is essential for re-review of the paper.

Please use the link below to submit your revised manuscript and related files:

[REDACTED]

Note: This URL links to your confidential home page and associated information about manuscripts you may have submitted, or that you are reviewing for us. If you wish to forward this email to co-authors, please delete the link to your homepage.

We hope to receive your revised manuscript within four to eight weeks. If you cannot send it within this time, please let us know. We will be happy to consider your revision so long as nothing similar has been accepted for publication at Nature Ecology & Evolution or published elsewhere.

Nature Ecology & Evolution is committed to improving transparency in authorship. As part of our efforts in this direction, we are now requesting that all authors identified as 'corresponding author' on published papers create and link their Open Researcher and Contributor Identifier (ORCID) with their account on the Manuscript Tracking System (MTS), prior to acceptance. ORCID helps the scientific community achieve unambiguous attribution of all scholarly contributions. You can create and link your ORCID from the home page of the MTS by clicking on 'Modify my Springer Nature account'. For more information please visit please visit www.springernature.com/orcid.

Please do not hesitate to contact me if you have any questions or would like to discuss these revisions further.

We look forward to seeing the revised manuscript and thank you for the opportunity to review your work.

[REDACTED]

Decision Letter, third revision:

13th May 2021

Dear Magnus,

Thank you for submitting your revised manuscript "Gradual evolution of allopolyploidy in <i>Arabidopsis suecica</i>" (NATECOLEVOL-200911576C).

I should note that your reluctance to accommodate any suggestions is disappointing. Two reviewers had valid criticisms about presentation of results, which likely reflect other views in the community. Our editorial suggestions were a good compromise and would likely improve how the paper will be

received by the community. Nevertheless, we don't think it is productive to insist on changes and given that there are no remaining technical issues, we agree in principle to publish the paper in Nature Ecology & Evolution, pending minor revisions to comply with formatting guidelines.

If the current version of your manuscript is in a PDF format, please email us a copy of the file in an editable format (Microsoft Word or LaTex)-- we can not proceed with PDFs at this stage.

We are now performing detailed checks on your paper and will send you a checklist detailing our editorial and formatting requirements in about a week. Please do not upload the final materials and make any revisions until you receive this additional information from us.

Thank you again for your interest in Nature Ecology & Evolution. Please do not hesitate to contact me if you have any questions.

[REDACTED]

Our ref: NATECOLEVOL-200911576C

25th May 2021

Dear Dr. Nordborg,

Thank you for your patience as we've prepared the guidelines for final submission of your Nature Ecology & Evolution manuscript, "Gradual evolution of allopolyploidy in <i>Arabidopsis suecica</i>" (NATECOLEVOL-200911576C). Please carefully follow the step-by-step instructions provided in the attached file, and add a response in each row of the table to indicate the changes that you have made. Please also check and comment on any additional marked-up edits we have proposed within the text. Ensuring that each point is addressed will help to ensure that your revised manuscript can be swiftly handed over to our production team.

We would like to start working on your revised paper, with all of the requested files and forms, as soon as possible (preferably within two weeks). Please get in contact with us immediately if you anticipate it taking more than two weeks to submit these revised files.

When you upload your final materials, please include a point-by-point response to any remaining reviewer comments.

If you have not done so already, please alert us to any related manuscripts from your group that are under consideration or in press at other journals, or are being written up for submission to other journals (see: https://www.nature.com/nature-research/editorial-policies/plagiarism#policy-on-duplicate-publication for details).

In recognition of the time and expertise our reviewers provide to Nature Ecology & Evolution's editorial process, we would like to formally acknowledge their contribution to the external peer review of your manuscript entitled "Gradual evolution of allopolyploidy in <i>Arabidopsis suecica</i>". For those reviewers who give their assent, we will be publishing their names alongside the published article.

Nature Ecology & Evolution offers a Transparent Peer Review option for new original research manuscripts submitted after December 1st, 2019. As part of this initiative, we encourage our authors to support increased transparency into the peer review process by agreeing to have the reviewer comments, author rebuttal letters, and editorial decision letters published as a Supplementary item. When you submit your final files please clearly state in your cover letter whether or not you would like to participate in this initiative. Please note that failure to state your preference will result in delays in accepting your manuscript for publication.

Cover suggestions

As you prepare your final files we encourage you to consider whether you have any images or illustrations that may be appropriate for use on the cover of Nature Ecology & Evolution.

Covers should be both aesthetically appealing and scientifically relevant, and should be supplied at the best quality available. Due to the prominence of these images, we do not generally select images featuring faces, children, text, graphs, schematic drawings, or collages on our covers.

We accept TIFF, JPEG, PNG or PSD file formats (a layered PSD file would be ideal), and the image should be at least 300ppi resolution (preferably 600-1200 ppi), in CMYK colour mode.

If your image is selected, we may also use it on the journal website as a banner image, and may need to make artistic alterations to fit our journal style.

Please submit your suggestions, clearly labeled, along with your final files. We'll be in touch if more information is needed.

Nature Ecology & Evolution has now transitioned to a unified Rights Collection system which will allow our Author Services team to quickly and easily collect the rights and permissions required to publish your work. Approximately 10 days after your paper is formally accepted, you will receive an email in providing you with a link to complete the grant of rights. If your paper is eligible for Open Access, our Author Services team will also be in touch regarding any additional information that may be required to arrange payment for your article.

Please note that <i>Nature Ecology & Evolution</i> is a Transformative Journal (TJ). Authors may publish their research with us through the traditional subscription access route or make their paper immediately open access through payment of an article-processing charge (APC). Authors will not be required to make a final decision about access to their article until it has been accepted. Find out more about Transformative Journals

<B>Authors may need to take specific actions to achieve compliance with funder and institutional open access mandates. For submissions from January 2021, if your research is supported by a funder that requires immediate open access (e.g. according to

Plan S principles) then you should select the gold OA route, and we will direct you to the compliant route where possible. For authors selecting the subscription publication route our standard licensing terms will need to be accepted, including our self-archiving policies. Those standard licensing terms will supersede any other terms that the author or any third party may assert apply to any version of the manuscript.

Please note that you will not receive your proofs until the publishing agreement has been received through our system.

For information regarding our different publishing models please see our Transformative Journals page. If you have any questions about costs, Open Access requirements, or our legal forms, please contact ASJournals@springernature.com.

Please use the following link for uploading these materials:

[REDACTED]

If you have any further questions, please feel free to contact me.

[REDACTED]

Final Decision Letter:

1st July 2021

Dear Dr Nordborg,

We are pleased to inform you that your Article entitled "Gradual evolution of allopolyploidy in <i>Arabidopsis suecica</i>", has now been accepted for publication in Nature Ecology & Evolution.

Before your manuscript is typeset, we will edit the text to ensure it is intelligible to our wide readership and conforms to house style. We look particularly carefully at the titles of all papers to ensure that they are relatively brief and understandable.

The subeditor may send you the edited text for your approval. Once your manuscript is typeset you will receive a link to your electronic proof via email, with a request to make any corrections within 48 hours. If you have queries at any point during the production process then please contact the production team at rjsproduction@springernature.com. Once your paper has been scheduled for online publication, the Nature press office will be in touch to confirm the details.

Acceptance of your manuscript is conditional on all authors' agreement with our publication policies (see www.nature.com/authors/policies/index.html). In particular your manuscript must not be published elsewhere and there must be no announcement of the work to any media outlet until the publication date (the day on which it is uploaded onto our web site).

Please note that <i>Nature Ecology & Evolution</i> is a Transformative Journal (TJ). Authors may publish their research with us through the traditional subscription access route or make their paper immediately open access through payment of an article-processing charge (APC). Authors will not be required to make a final decision about access to their article until it has been accepted. Find out more about Transformative Journals

<B>Authors may need to take specific actions to achieve compliance with funder and institutional open access mandates. For submissions from January 2021, if your research is supported by a funder that requires immediate open access (e.g. according to Plan S principles) then you should select the gold OA route, and we will direct you to the compliant route where possible. For authors selecting the subscription publication route our standard licensing terms will need to be accepted, including our self-archiving policies. Those standard licensing terms will supersede any other terms that the author or any third party may assert apply to any version of the manuscript.

In approximately 10 business days you will receive an email with a link to choose the appropriate publishing options for your paper and our Author Services team will be in touch regarding any additional information that may be required.

You will not receive your proofs until the publishing agreement has been received through our system.

If you have any questions about our publishing options, costs, Open Access requirements, or our legal forms, please contact ASJournals@springernature.com

An online order form for reprints of your paper is available at <a

href="https://www.nature.com/reprints/author-reprints.html">https://www.nature.com/reprints/author-reprints.html. All co-authors, authors' institutions and authors' funding agencies can order reprints using the form appropriate to their geographical region.

We welcome the submission of potential cover material (including a short caption of around 40 words) related to your manuscript; suggestions should be sent to Nature Ecology & Evolution as electronic files (the image should be 300 dpi at 210 x 297 mm in either TIFF or JPEG format). Please note that such pictures should be selected more for their aesthetic appeal than for their scientific content, and that colour images work better than black and white or grayscale images. Please do not try to design a cover with the Nature Ecology & Evolution logo etc., and please do not submit composites of images related to your work. I am sure you will understand that we cannot make any promise as to whether any of your suggestions might be selected for the cover of the journal.

You can now use a single sign-on for all your accounts, view the status of all your manuscript submissions and reviews, access usage statistics for your published articles and download a record of your refereeing activity for the Nature journals.

To assist our authors in disseminating their research to the broader community, our SharedIt initiative provides you with a unique shareable link that will allow anyone (with or without a subscription) to read the published article. Recipients of the link with a subscription will also be able to download and print the PDF.

You can generate the link yourself when you receive your article DOI by entering it here: <a href="http://authors.springernature.com/share">http://authors.springernature.com/share<a>.

[REDACTED]

P.S. Click on the following link if you would like to recommend Nature Ecology & Evolution to your librarian http://www.nature.com/subscriptions/recommend.html#forms

** Visit the Springer Nature Editorial and Publishing website at www.springernature.com/editorial-and-publishing-jobs for more information about our career opportunities. If you have any questions please click here.**
